# Phosphine Catalyzed Michael-Type Additions: The Synthesis of Glutamic Acid Derivatives from Arylidene-α-amino Esters †

**DOI:** 10.3390/molecules29020342

**Published:** 2024-01-10

**Authors:** Lesly V. Rodríguez-Flórez, María González-Marcos, Eduardo García-Mingüens, María de Gracia Retamosa, Misa Kawase, Elisabet Selva, José M. Sansano

**Affiliations:** 1 Departamento de Química Orgánica, Centro de Innovación en Química Avanzada (ORFEO-CINQA) and Instituto de Síntesis Orgánica, Universidad de Alicante, Ctra. Alicante-San Vicente s/n, 03080 Alicante, Spain; 2 Medalchemy, S. L. Ancha de Castelar, 46-48, entlo. A. San Vicente del Raspeig, 03690 Alicante, Spain

**Keywords:** organocatalysis, phosphines, imino esters, Michael addition, glutamates, pyroglutamates

## Abstract

The reaction of arylidene-α-amino esters with electrophilic alkenes to yield Michael-type addition compounds is optimized using several phosphines as organocatalysts. The transformation is very complicated due to the generation of several final compounds, including those derived from the 1,3-dipolar cycloadditions. For this reason, the selection of the reaction conditions is a very complex task and the slow addition of the acrylic system is very important to complete the process. The study of the variation in the structural components of the starting imino ester is performed as well as the expansion of other electron-poor alkenes. The crude products have a purity higher than 90% in most cases without any purification. A plausible mechanism is detailed based on the bibliography and the experimental results. The synthesis of pyroglutamate entities, after the reduction of the imino group and cyclization, is performed in high yields. In addition, the hydrolysis of the imino group, under acidic media, represents a direct access to glutamate surrogates.

## 1. Introduction

**Scheme 1:**
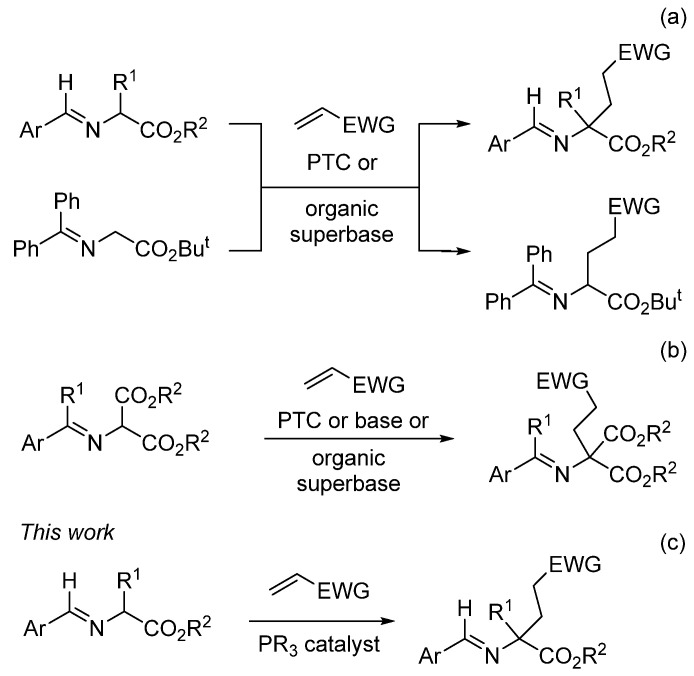
(**a**–**c**) Strategies for the preparation of glutamate derivatives.

Proteinogenic and non-proteinogenic α-amino acids (AAs) constitute one of the five most important families of essential molecules in many scientific areas. The synthesis of these compounds [1,2,3] obeys several general patterns such as the following [4,5,6,7,8,9,10,11,12,13]: (a) the introduction of the hydrogen atom in the appropriate carbon-carbon or carbon-nitrogen double bond structures; (b) the employment of a methodology able to insert the nitrogen atom at the α-position to the ester group (electrophilic nitrogen source); (c) reactions involving the incorporation of a carboxy group and (d) the coupling of the α-side chain to the AA template. Considering this last approach, α-substituted glutamates have been mainly obtained via the Michael-type additions of glycine derivatives (glycine templates) onto the corresponding α,β-unsaturated reagents. This reliable strategy employs *N*-arylidene-α-amino acid esters [14,15,16] or *tert*-butyl *N*-benzylidieneamino glycinate [17,18,19,20,21,22] (Scheme 1a) and even activated *N*-arylideneaminomalonates [23,24,25,26,27,28,29,30] (Scheme 1b) as starting materials. In all cases, phase transfer catalysis (PTC) conditions or the employment of organic superbases are the most common trends to complete the reaction. An important drawback detected in the reactions regarding glycine templates is the double alkylation process at the α-position.

Having in mind the natural impact [31] and usefulness of glutamates (and their pyroglutamate surrogates) [32] as synthetic key building blocks and their presence in many biologically active molecules [33,34,35,36,37,38,39,40,41,42,43], we have studied a novel Michael-type approach to their preparation. This methodology consists of the base-free reaction of alkyl *N*-arylidene-α-amino acid esters with conjugated alkenes [44]. Here, the transformation operates in the presence of a substoichiometric amount of a phosphine, which acts as an organocatalyst (Scheme 1c) [45].

## 2. Results and Discussion

**Scheme 2:**
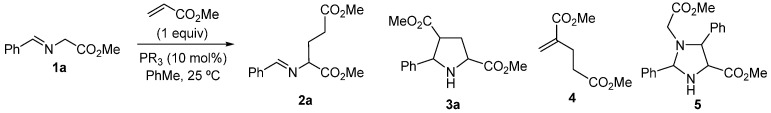
Optimization of the Michael-type addition of imino ester **1a** onto methyl acrylate (MA).

The reaction between imino esters **1** and Michael acceptors can be controlled to afford the pure conjugated product or the corresponding pyrrolidine via 1,3-dipolar cycloaddition (1,3-DC) [46,47,48,49,50,51,52,53]. These two products are found in the final crude mixture in some cycloadditions. With the aim of selecting the formation of the glutamate structure, we consider the ability of phosphines to catalyze this process. Thus, the reaction of imino ester **1a** (1 equiv) with methyl acrylate (1 equiv) was treated with the corresponding phosphine (10 mol% loading), using toluene as a solvent, at 25 °C (Scheme 2 and Table 1). The optimization of this reaction was a very complicated task due to the presence of three identified secondary compounds (**3a**, **4** and **5**, see experimental part and Supplementary Information). Initially, the nature of the phosphine was tested. The nucleophilicity of a triarylphosphine, such as Ph_3_P, was not enough to promote the desired reaction (Table 1, entry 1). 1,2-Bis(diphenylphophino)ethane (dppe) did not complete the reaction after 15 h, affording **2a** impurified with cycloadducts **3a** and **5** (Table 1, entry 2). The consumption of the starting material **1a** was achieved after 72 h of reaction, but these two impurities were detected as shown in entries 3 and 4 of Table 1. The slow addition (60 min) of imino ester **1a** to the reaction mixture avoided the 1,3-DC, although it promoted the generation of the diester **4** as a consequence of the presence of an excess of the alkene (Table 1, entry 5). Byproduct **4** was suppressed with the slow addition of the methyl acrylate (60 min), but imidazolidine **5** was observed instead after 72 h of reaction (Table 1, entry 6). A shorter reaction time avoided the completion of the reaction and obtained significant amounts of starting imino ester **1a** (Table 1, entry 7). Both tri-*n*-butyl and tri-*tert*-butyl phosphines exclusively afforded compound **5** or the diester **4**, even in the process involving a slow addition of the acrylate (Table 1, entries 8–10). Perhaps, the nucleophilicity of trialkyl phosphines is excessive for controlling the desired process. So, the modulation of this property via combining an aryl group with two alkyl substituents bonded to the phosphorous atom, was next attempted. Then, Me_2_PhP was used as a catalyst, demonstrating a rapid conversion (2 h) but generating large amounts of compounds **4** and **5** (Table 1, entries 11 and 12). Longer addition times (60 min) of the methyl acrylate favored the formation of the cycloaddition products **3a** and **5**. However, lower addition times contributed to an increase in the presence of compound **4**. The optimal addition time of methyl acrylate was 30 min (Table 1, entries 11–16) furnishing a very clean reaction crude product using ^1^H NMR. In fact, compound **2a** did not require any additional purification after the work-up (see experimental section). The lowering of the catalyst loading to 5 mol% did not efficiently promote the reaction (Table 1, entry 17). The effect of the solvent was not significant, obtaining similar results when the reactions were performed in dichloromethane, THF or acetonitrile. Solvents such as MTBE, EtOAc, acetone and water were not suitable. No reaction product **2a** was identified in the ^1^H NMR spectra when dimethylphenylphosphine was substituted with the same loading of triethylamine, DABCO or DBU as catalysts. All the product ratios detailed in Table 1 were accurately analyzed using ^1^H NMR integrals of these crude materials.

**Scheme 3:**
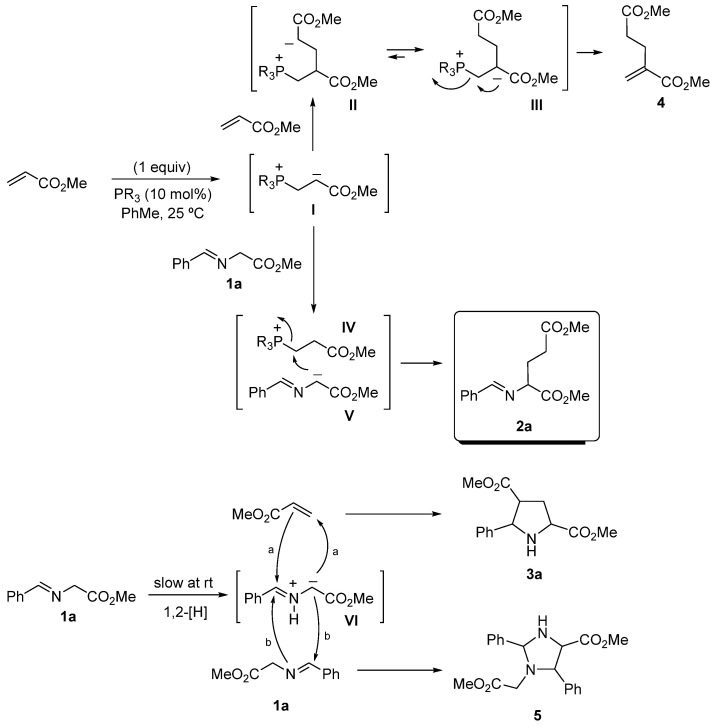
Mechanism of the formation of products **2a**, **3a**, **4** and **5**.

The plausible mechanism of all these processes is described in Scheme 3. The excess of methyl acrylate in the reaction media causes the 1,4-attack of the intermediate **I** (generated by the Michael-type addition of the phosphine and the acrylate) on another equivalent of methyl acrylate. After a prototropy shift caused by the stabilization of the negative charge in intermediate **III**, the dimer **4** is formed with the regeneration of the catalyst. The low amounts of the intermediate **I**, obtained after the slow addition of methyl acrylate, are surrounded by a large excess of imino ester **1a**, which can be deprotonated by enolate **I**, furnishing the stabilized carbanion **V**. The direct attack of **V** on **IV** gives the desired Michael-type adduct **2a** with the elimination of the active catalyst. The fine-tuning of the nucleophilicity of the phosphine in the last step is crucial and the overall mechanism is very sensitive to this feature. On the other hand, when the addition of the phosphine is very slow, or simply does not occur, the excess of **1a** can give the fleeting azomethine ylide **VI** after a 1,2-prototropy shift at room temperature. This process is very slow, but the ylide **VI** is trapped immediately by methyl acrylate (which does not undergo the transformation to the corresponding intermediate **I**), giving access to cycloadduct **3a**. This is a very fast reaction compared with the analogous Mannich type-cyclization to yield product **5**. In consequence, entry 15 of Table 1 employs the optimal phosphine. This phosphine is able to generate intermediate **I**, which has a preference for abstracting the α-H of the imino ester **1a** rather than other different reactions. The slow addition of the acrylate inhibits the route to yield dimer **4**, but favors the route to generate the expected compound **2a**. There is a paramount detail in this last step. The phosphine does not activate the imino group, neither the imino ester **1a** nor other different intermediate species, such as **V** or even **VI**. The absence of the route **I** → **IV** → **V** favors the presence of **4**, allowing the generation of the ylide **VI** and giving rise to pyrrolidine **3a** (after reaction with methyl acrylate) or imidazolidine **5** (via the self-addition of **1a**). Products **3a** and **5** are formed when the addition took 60 min and they are minimized when performing the addition in 30 min (Table 1, entries 14 and 15). However, an alternative base-propagation mechanism where the enolate **V** promotes a Michael-type addition, and not a S_N_2 onto the phosphonium intermediate, cannot be discarded [54].

**Scheme 4:**
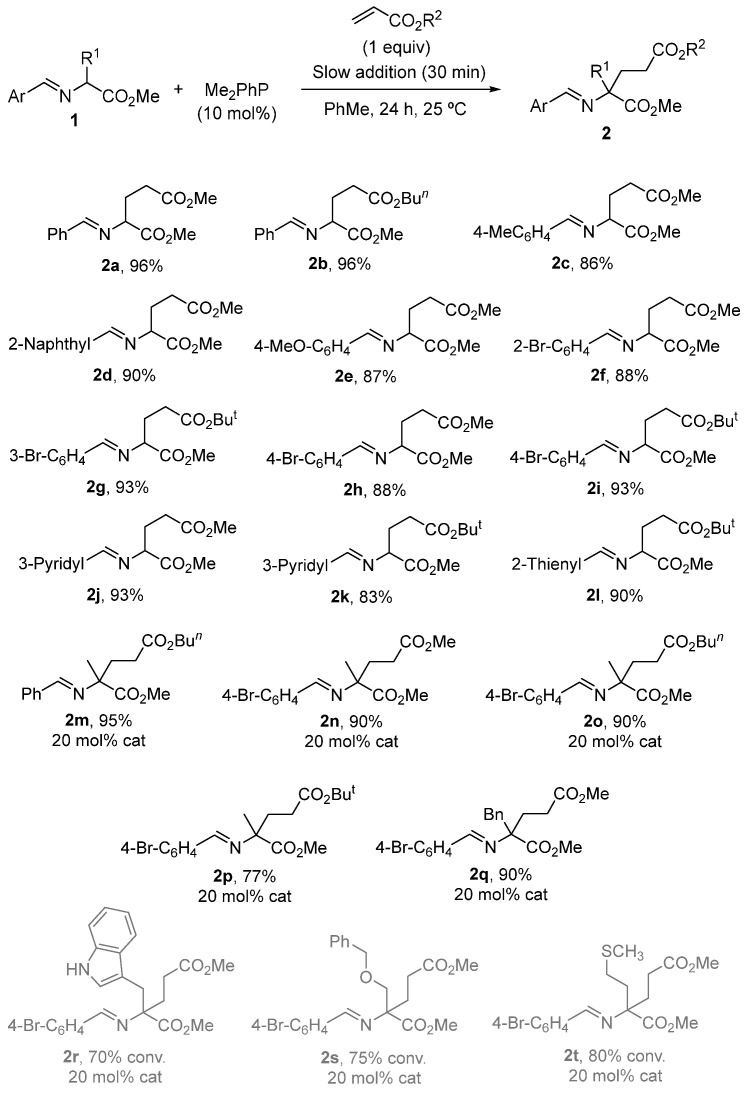
Scope of the Michael-type addition of imino esters **1** onto alkyl acrylates.

With the best reaction conditions established in entry 15 of Table 1, the scope of imino esters **1** and alkyl acrylates was investigated. The results of the crude yields, determined by ^1^H NMR spectra, using dimethyl terephthalate as an internal standard [55], are depicted in Scheme 3 [56]. The variation in the aromatic moiety in glycinate-derived imino esters was well tolerated (**2a**–**l**, Scheme 4), with even starters containing heterocyclic units as 2-thienyl or 3-pyridyl (**2j**–**l**, Scheme 4). Methyl, *n*-butyl and *tert*-butyl acrylates were randomly employed, giving satisfactory results (Scheme 4) [57]. However, when imino esters with a substituent at the α-position were tested, a 20 mol% of the catalyst loading was required to complete the transformations. Also, different slow addition times and an excess of the acrylate component were modified accordingly in these examples to obtain the best yields and purities (see experimental part). Thus, glutamate derivatives **2m**–**q** were obtained (and characterized without purification) in very good yields (Scheme 4). However, with functionalized α-side chain α-amino acid-derived imino esters **1**, such as tryptophan, *O*-benzylserine and methionine, the conversions were good but the crude compounds **2r**–**t** were not pure and could not be characterized (grey color in Scheme 4). These three last examples were immediately transformed into the corresponding pyroglutamate surrogates **8** (see below in Scheme 6b). Despite the large quantity of secondary products expected, the final compounds were obtained as crude materials with purities higher than 90% in most cases (see experimental part) without any purification. Chromatographic separation was not possible for those of lower purity due to the formation of amines and aldehydes from the imines on SiO_2_.

**Scheme 5:**
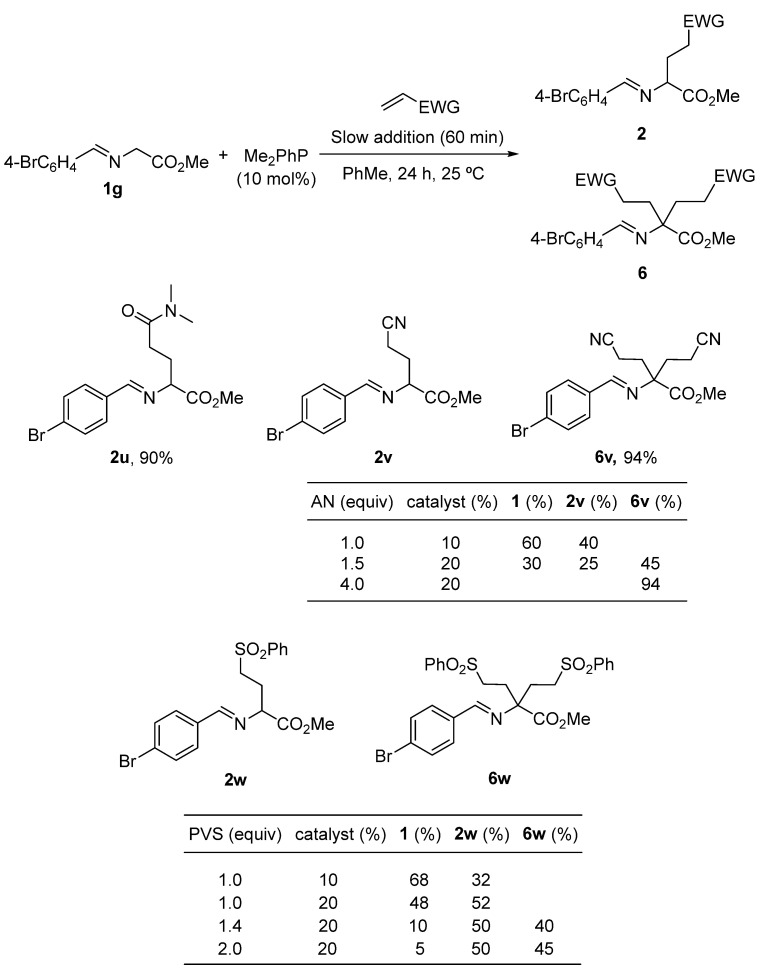
Michael-type addition of imino ester **1g** onto AN and PVS.

Other acrylic systems like *N,N*-dimethylacrylamide reacted satisfactorily under these conditions, affording the glutamine derivative **2u** with a 90% yield. In the presence of acrylonitrile or phenyl vinyl sulfone (1 equiv), the corresponding molecules **6**, originating from a double addition of the alkene, were detected as byproducts. The full conversion of the reaction performed with acrylonitrile was achieved using a 20 mol% of the catalyst and 4 equiv of the Michael-type acceptor. After that, the α,α-disubstituted imino ester **6v** was obtained with a 94% yield (by ^1^H NMR, Scheme 5). Bulkier phenyl vinyl sulfone did not afford pure and clean compound **6w** due to the presence of tantamount quantities of monoalkylated substance **2w**, even working in the presence of an excess of alkene and using 20 mol% of the catalyst (Scheme 5). This preference for the phenyl vinyl sulfone and acrylonitrile to generate double alkylation products **6**, unlike the acrylic esters and acrylamide, is due to the existence of a lower energy LUMO. LUMO’s energies of phenyl vinyl sulfone and acrylonitrile are −1.891 [58] and −2.52 eV [59], respectively, whilst the LUMO’s energy of the methyl acrylate is −0.08 eV [60].

**Scheme 6:**
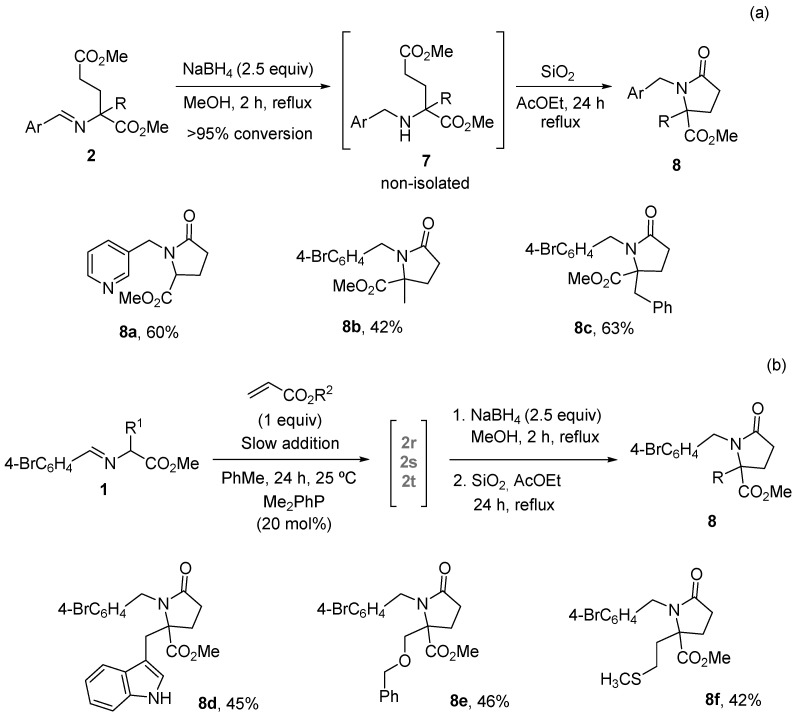
Synthesis of pyroglutamate derivatives **8**. (**a**) From compounds **2**. (**b**) From compounds **1** without isolating intermediate molecules **2**.

**Scheme 7:**
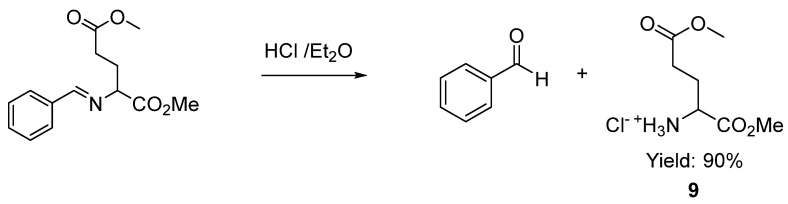
Synthesis of glutamate derivative **9**.

A straightforward access to pyroglutamates, which are key units in biotechnology, in biomedicine and for the treatment of neurodegenerative illnesses [32,61,62,63,64], is easily envisaged. Employing conventional transformations, such as reduction with sodium borohydride followed by mild cyclization conditions using silica gel in refluxing ethyl acetate, substituted pyroglutamates **8** were isolated in moderate to good yields after flash chromatography (Scheme 6a). Non-isolated adducts **2r**–**t**, described in Scheme 4, were directly submitted to these sequential reduction-cyclization conditions, obtaining the pyroglutamates **8d**, **8e** and **8f** in 45, 46 and 42% overall yields, respectively (from imino ester **1**, Scheme 6b). In addition, the rapid access to glutamic ester derivative **9** was achieved with a 90% yield via treatment with 2M HCl/Et_2_O (Scheme 7).

## 3. Materials and Methods

### 3.1. General

All commercially available reagents and solvents were used without further purification, only aldehydes were also distilled prior to use. Analytical TLC was performed on Schleicher & Schuell F1400/LS 254 (Schleicher & Schuell BioScience GmbH, Dassel, Germany) silica gel plates, and the spots were visualized under UV light (λ = 254 nm). Flash chromatography was carried out on hand-packed columns of Merck silica gel 60 (0.040–0.063 mm). Melting points were determined with a Reichert Thermovar hot plate apparatus and are uncorrected. The structurally most important peaks of the IR spectra (recorded using a Nicolet 510 P-FT (Thermo-Fisher Scientific, Waltham, MA, USA) are listed and wavenumbers are given in cm^−1^. NMR spectra were obtained using a Bruker AC-300 (Bruker Corporation, Billerica, MA, USA) or AC-400 (Bruker Corporation, Billerica, MA, USA) and were recorded at 300 or 400 MHz for ^1^H NMR and 75 or 100 MHz for ^13^C{1H} NMR, using CDCl_3_ as the solvent and TMS as the internal standard (0.00 ppm) unless otherwise stated. The following abbreviations are used to describe peak patterns where appropriate: s = singlet, d = doublet, t = triplet, q = quartet, m = multiplet or unresolved and br s = broad signal. All coupling constants (*J*) are given in Hz and chemical shifts in ppm. ^13^C{1H} NMR spectra were referenced to CDCl_3_ at 77.16 ppm. Chemical yields and purities of compounds **2** and **6** were calculated by the integration of ^1^H NMR spectra using dimethyl terephthalate as the internal standard [17]. Low-resolution electron impact (EI) mass spectra were obtained at 70 eV using a Shimadzu QP-5000 via injection or DIP; fragment ions in *m*/*z* are given with relative intensities (%) in parentheses. High-resolution mass spectra (HRMS) were measured on an instrument using a quadrupole time-of-flight mass spectrometer (QTOF) and also through the electron impact mode (EI) at 70 eV using a Finnigan VG Platform or a Finnigan MAT 95S. 

The synthesis of the starting α-imino esters **1** was performed following the described procedure [65,66]. Thus, the corresponding α-amino acid alkyl ester hydrochloride (3.0 mmol), the corresponding aldehyde (2.3 mmol) and MgSO_4_ were dissolved in dry dichloromethane (5 mL). Then, triethylamine (3.0 mmol) was slowly added and the mixture was then stirred for 18 h. Later, the reaction mixture was filtered, extracted with dichloromethane (3 × 10 mL), dried over MgSO_4_ and evaporated under reduced pressure, obtaining **1**, which was employed without further purification.

Compounds **3a** [66,67], **4** [68] and **5** [69] were obtained using procedures described in the literature just to compare the signals for the determination of the proportions depicted in Table 1 (see Supplementary Materials).

### 3.2. General Experimental Procedure for the Synthesis of Michael Type Addition Products 2

In a round-button flask and under argon, the corresponding iminoester **1** (0.5 mmol) in toluene (1.5 mL) and the catalyst dimethylphenylphosphine (0.05 mmol) were added. Then a solution of electrophilic alkene (1 mmol) in toluene (1 mL) was added dropwise over a 30 min period using an addition pump. Then, the mixture was stirred at room temperature for 24 h. The solvent was removed under reduced pressure, to afford the corresponding final product **2**.

Dimethyl (*E*)-2-(benzylideneamino)pentanedioate (**2a**): Pale yellow oil (118.2 mg, 96%, 92% purity). IR (neat) ν_max_: 1735, 1643, 1438, 1253, 1199, 1164, 755, 694 cm^−1^. ^1^H NMR (400 MHz) δ 8.29 (s, 1H, *H*C=N), 7.82–7.72 (m, 2H, ArH), 7.48–7.37 (m, 3H, ArH), 4.07 (dd, *J* = 8.1, 5.0 Hz, 1H, NC*H*CO_2_Me), 3.74 (s, 3H, CO_2_CH_3_), 3.65 (s, 3H, CO_2_CH_3_), 2.43–2.38 (m, 2H, CH_2_), 2.36–2.23 (m, 2H, CH_2_). ^13^C NMR (101 MHz) δ 173.3, 171.9 (C=O), 164.3 (C=N), 135.5(*C*Ar), 131.3(*C*HAr), 128.6 (4x *C*HAr), 71.8 (*C*H), 52.2, 51.6 (*C*H_3_), 30.2, 28.3 (*C*H_2_). MS (EI) *m*/*z*: 263 (M^+^, 11%), 204 (74), 203 (38), 190 (43), 144 (100), 130 (29), 117 (31), 104 (39), 90 (21). HRMS (ESI): *m*/*z* calcd for C_14_H_17_NO_4_ [M^+^] 263.1158; found: 263.1159.

5-Butyl 1-methyl (*E*)-2-(benzylideneamino)pentanedioate (**2b**): Pale yellow oil (146 mg, 96%, 91% purity). IR (neat) v_max_: 1731, 1643, 1438, 1390, 1168, 1068, 732, 694 cm^−1^. ^1^H NMR (400 MHz) δ 8.26 (s, 1H, *H*C=N), 7.90–7.66 (m, 2H, ArH), 7.51–7.21 (m, 3H, ArH), 4.37–3.92 (m, 3H, NC*H*CO_2_Me, CO_2_C*H*_2_CH_2_CH_2_CH_3_), 3.86–3.55 (m, 3H, CO_2_CH_3_), 2.34 (d, *J* = 2.0 Hz, 2H, NCHCH_2_C*H*_2_), 2.33–1.88 (m, 2H, NCHC*H*_2_CH_2_), 1.70–1.41 (m, 2H, CO_2_CH_2_C*H*_2_CH_2_CH_3_), 1.41–1.19 (m, 2H, CO_2_CH_2_CH_2_C*H*_2_CH_3_), 1.07–0.68 (m, 3H, CO_2_CH_2_CH_2_CH_2_C*H*_3_). ^13^C NMR (101 MHz) δ 172.88 (C=O), 171.90 (C=O), 164.25 (C=N), 135.53 (*C*Ar), 131.26 (*C*HAr), 128.59 (*C*HAr x4), 71.81 (*C*H), 64.34 (*C*H_2_), 52.19 (*C*H_3_), 30.61 (*C*H_2_), 30.41 (*C*H_2_), 28.33 (*C*H_2_), 19.09 (*C*H_2_), 13.66 (*C*H_3_). MS (EI) *m*/*z*: 305 (M+, 17%), 246 (66), 232 (38), 190 (81), 144 (100). HRMS (ESI): *m*/*z* calcd for C_17_H_23_NO_4_ [M^+^] 305.1627; found: 305.1622.

Dimethyl (*E*)-2-((4-methylbenzylidene)amino)pentanedioate (**2c**): Pale yellow oil (119.4 mg, 86%, 95% purity). IR (neat) ν_max_: 1735, 1643, 1438, 1253, 1199, 1168, 813 cm^−1^. ^1^H NMR (300 MHz) δ 8.25 (s, 1H, *H*C=N), 7.66 (d, *J* = 8.1 Hz, 2H, ArH), 7.22 (d, *J* = 7.7 Hz, 2H, ArH), 4.04 (dd, *J* = 8.0, 4.9 Hz, 1H, NC*H*CO_2_Me), 3.74 (s, 3H, CO_2_CH_3_), 3.64 (s, 3H, CO_2_CH_3_), 2.39 (s, 3H, C*H*_3_Ar), 2.38–2.22 (m, 4H, C*H*_2_-C*H*_2_). ^13^C NMR (101 MHz) δ 173.5 (*C*=O), 172.3 (*C*=O), 164.2 (*C*=N), 141.7 (*C*Ar), 132.9 (CH_3_*C*Ar), 129.3 (*C*HAr), 128.6 (*C*HAr), 71.8 (*C*H), 52.2, 51.6 (*C*H_3_), 30.1, 28.3 (*C*H_2_), 21.5 (*C*H_3_CAr). MS (EI) *m*/*z*: 277 (M^+^, 17%), 218 (64), 217 (32) 204 (48), 158 (100), 144 (34), 131 (28), 130 (24), 118 (40). HRMS (ESI): *m*/*z* calcd for C_15_H_19_NO_4_ [M^+^] 277.1314; found: 277.1322.

Dimethyl (*E*)-2-[(naphth-2-ylmethylene)amino)]pentanedioate (**2d**): Pale yellow prisms (147.2 mg, 90%, 92% purity). mp: 78–79 °C (n-hexane:AcOEt). IR (neat) ν_max_: 1727, 1639, 1434, 1176, 1095, 829, 752 cm^−1^. ^1^H NMR (300 MHz) δ 8.46 (s, 1H, *H*C=N), 8.10 (s, 1H, ArH), 8.03 (dd, *J* = 8.6, 1.4 Hz, 1H, ArH), 7.94–7.81 (m, 3H, ArH), 7.59–7.47 (m, 2H, ArH), 4.15 (dd, *J* = 7.7, 5.0 Hz, 1H, NC*H*CO_2_Me), 3.78 (s, 3H, CO_2_CH_3_), 3.66 (s, 3H, CO_2_CH_3_), 2.49–2.42 (m, 2H, C*H*_2_), 2.41–2.28 (m, 2H, C*H*_2_). ^13^C NMR (101 MHz) δ 173.3 (*C*=O), 171.9 (*C*=O), 164.3 (*C*=N), 134.9 (C=*C*CH=N), 133.2, 133.0 (Ph*C*), 130.7, 128.7, 128.5, 127.9, 127.6, 126.6, 124.0 (Ph*C*H), 71.8 (N*C*H), 52.2, 51.6 (*C*H_3_), 30.2, 28.4 (*C*O_2_CH_3_). MS (EI) *m*/*z*: 313 (M^+^, 31%), 254 (100), 240 (61), 222 (28), 194 (99), 180 (69), 167 (66), 154 (57), 140 (30), 139 (51), 127 (24). HRMS (ESI): *m*/*z* calcd for C_18_H_19_NO_4_ [M^+^] 313.1314; found: 313.1331.

Dimethyl (*E*)-2-[(4-methoxybenzylidene)amino]pentanedioate (**2e**): yellow oil (141.9 mg, 87%, 92% purity). IR (neat) ν_max_: 1735, 1249, 1164, 1025, 833 cm^−1^. ^1^H NMR (400 MHz) δ 8.21 (s, 1H, *H*C=N), 7.75–7.69 (m, 2H, ArH), 6.95–6.90 (m, 2H, ArH), 4.02 (dd, *J* = 8.1, 5.0 Hz, 1H, NC*H*CO_2_Me), 3.84 (s, 3H, CO_2_CH_3_), 3.74 (s, 3H, CO_2_CH_3_), 3.64 (s, 3H, C*H*_3_OAr), 2.45–2.15 (m, 4H, C*H*_2_-C*H*_2_). ^13^C NMR (75 MHz) δ 173.34 (*C*=O), 172.13 (*C*=O), 163.54 (*C*=N), 162.15 (OCH_3_*C*Ar), 130.26 (2x*C*HAr), 128.52 (*C*Ar), 114.00 (2x*C*HAr), 71.75(*C*H), 55.38 (O*C*H_3_CAr), 52.19, 51.58 (CO_2_*C*H_3_), 30.22, 28.39 (*C*H_2_). MS (EI) *m*/*z*: 293 (M^+^, 25%), 262 (21), 234 (69), 233 (41), 220 (62), 174 (100), 160 (29), 147 (23), 134 (40). HRMS (ESI): *m*/*z* calcd for C_15_H_19_NO_4_ [M^+^] 293.1263; found: 293.1262.

5-(*Tert*-butyl) 1-methyl (*E*)-2-[(2-bromobenzylidene)amino]pentanedioate (**2f**): Pale yellow oil (196.6 mg, 88%, 91% purity). IR (neat) ν_max_: 1727, 1149, 752, 686 cm^−1^. ^1^H NMR (300 MHz) δ 8.65 (s, 1H, *H*C=N), 8.08 (dd, *J* = 7.6, 2.0 Hz, 1H, ArH), 7.60–7.55 (m, 1H, ArH), 7.38–7.28 (m, 2H, ArH), 4.19–4.08 (m, 1H, NC*H*CO_2_Me), 3.76 (s, 3H, C*H*_3_), 2.37–2.22 (m, 4H, C*H*_2_-C*H*_2_), 1.44 [s, 9H, (CH_3_)_3_]. ^13^C NMR (101 MHz) δ 172.02 (*C*=O_2_^t^Bu), 171.85 (*C*O_2_Me), 163.16 (*C*H=N), 134.01 (*C*Ar), 133.03 (*C*HAr), 132.37 (*C*HAr), 129.27 (*C*HAr), 127.63(*C*HAr), 125.36 (Br*C*Ar), 80.54 (*C*(CH_3_)_3_), 71.83(N*C*HCO_2_Me), 52.29 (*C*H_3_), 31.49 (*C*H_2_), 28.45 (*C*H_2_), 28.11 (C(*C*H_3_)_3_). MS (EI) *m*/*z*: 384 (M^+^, 1%), 329 (47), 327 (48), 312 (38), 310 (38), 276 (93), 269 (44), 268 (100), 224 (41), 222 (37), 184 (41), 89 (50), 57 (66). HRMS (ESI): *m*/*z* calc for C_17_H_22_BrNO_4_ [M^+^] 383.073; found: 385.0694.

5-(*Tert*-butyl) 1-methyl (*E*)-2-[(3-bromobenzylidene)amino]pentanedioate (**2g**): Pale yellow oil (215.3 mg, 93%, 92% purity). IR (neat) ν_max_: 1727, 1369, 1149, 752, 682 cm^−1^. ^1^H NMR (400 MHz) δ 8.22 (s, 1H, *H*C=N), 7.98 (t, *J* = 1.8 Hz, 1H, ArH), 7.66–7.62 (m, 1H, ArH), 7.58–7.54 (m, 1H, ArH), 7.28 (t, *J* = 7.8 Hz, 1H, ArH), 4.09–4.04 (m, 1H, NC*H*CO_2_Me), 3.74 (s, 3H, CO_2_C*H*_3_), 2.30–2.15 (m, 4H, C*H*_2_-C*H*_2_), 1.43 [s, 9H, CO_2_(CH_3_)_3_]. ^13^C NMR (101 MHz) δ 172.0 (*C*=O), 171.8 (*C*=O), 162.5 (*C*H=N), 137.4 (*C*Ar), 134.1 (*C*HAr), 131.0 (*C*HAr), 130.1 (*C*HAr), 127.5 (*C*HAr), 122.9 (Br*C*Ar), 80.5 (*C*(CH_3_)_3_), 71.7 (N*C*HCO_2_Me), 52.3 (CO_2_*C*H_3_), 31.5 (*C*H_2_), 28.4 (*C*H_2_), 28.1 [(*C*H_3_)_3_]. MS (EI) *m*/*z*: 384 (M^+^, 7%), 329 (43), 327 (45), 310 (38, 268 (100), 224 (43), 222 (40), 184 (42), 116 (36), 89 (50), 57 (93). HRMS (ESI): *m*/*z* calc for C_17_H_22_BrNO_4_ [M^+^] 383.073; found: 383.0737.

Dimethyl (*E*)-2-[(4-bromobenzylidene)amino]pentanedioate (**2h**): Pale yellow oil (151.3 mg, 88%, 93% purity). IR (neat) ν_max_: 1731, 2116, 1068, 1010, 821, 732 cm^−1^. ^1^H NMR (300 MHz) δ 8.24 (s, 1H, *H*C=N), 7.68–7.61 (m, 2H, ArH), 7.59–7.52 (m, 2H, ArH), 4.15–3.98 (m, 1H, NC*H*CO_2_Me), 3.75 (s, 3H, CO_2_CH_3_), 3.65 (s, 3H, CO_2_CH_3_), 2.47–2.14 (m, 4H, C*H*_2_-C*H*_2_). ^13^C NMR (101 MHz) δ 173.24 (*C=*O), 171.69 (*C=*O), 163.07 (C=N), 134.39 (Br*C*Ar), 131.9, 129.9 (*C*HAr), 125.8 (*C*Ar), 71.6 (*C*H), 52.3, 51.6 (*C*H_3_), 30.1, 28.2 (*C*H_2_). MS (EI) *m*/*z*: 342 (M^+^, 4%), 282 (87), 268 (55), 222 (100), 184 (54), 143 (42), 116 (40), 89 (88). HRMS (ESI): *m*/*z* calcd for C_14_H_16_BrNO_4_ [M^+^] 341.0263; found: 341.0265.

5-(*Tert*-butyl) 1-methyl (*E*)-2-[(4-bromobenzylidene)amino]pentanedioate (**2i**): Pale yellow oil (139.1 mg, 93%, 93% purity). IR (neat) ν_max_: 1727, 1369, 1149, 825 cm^−1^. ^1^H NMR (300 MHz) δ 8.23 (s, 1H, *H*C=N), 7.65 (d, *J* = 8.5 Hz, 2H, ArH), 7.56 (d, *J* = 8.5 Hz, 2H, ArH), 4.10–4.02 (m, 1H, NC*H*CO_2_Me), 3.75 (s, 3H, CO_2_CH_3_), 2.34–2.15 (m, 4H, C*H*_2_-C*H*_2_), 1.43 (s, 9H, C(C*H*_3_)_3_). ^13^C NMR (101 MHz) δ 172.2 (*C=*O), 172.0 (C=O), 163.0 (*C*=N), 134.5 (Br*C*Ar), 132.0 (*C*HAr), 130.1 (*C*HAr), 125.9 (*C*Ar), 80.6 [*C*(CH_3_)_3_], 71.9 (*C*H), 52.4 (CO_2_*C*H_3_), 31.7 (CH_2_), 28.6 (CH_2_), 28.2 [CO_2_C(*C*H_3_)_3_]. MS (EI) *m*/*z*: 383(M^+^, 3%), 327 (49), 312 (42), 268 (100), 224 (48), 184 (40), 89 (63), 57(92). HRMS (ESI): *m*/*z* calc for C_17_H_22_BrNO_4_ [M^+^] 383.0730; found: 383.0720.

Dimethyl (*E*)-2-[(pyridin-3-ylmethylene)amino]pentanedioate (**2j**): Pale yellow oil (214.7 mg, 93%, 90% purity). IR (neat) ν_max_: 1731, 1434, 1172, 806, 709 cm^−1^. ^1^H NMR (300 MHz) δ 8.86 (dd, *J* = 2.1, 0.7 Hz, 1H, ArH), 8.65 (dd, *J* = 4.8, 1.7 Hz, 1H, ArH), 8.32 (s, 1H, *H*C=N), 8.15 (dt, *J* = 7.9, 1.9 Hz, 1H, ArH), 7.34 (dd, *J* = 7.9, 4.8 Hz, 1H, ArH), 4.10 (dd, *J* = 7.7, 5.0 Hz, 1H, N*H*CO_2_Me), 3.73 (s, 3H, C*H*_3_), 3.63 (s, 3H, C*H*_3_), 2.42–2.19 (m, 4H, C*H*_2_-C*H*_2_). ^13^C NMR (101 MHz) δ 173.2 (*C*=O), 171.5 (*C*=O), 161.6 (*C*=N), 152.1 (*C*HAr), 150.6 (*C*HAr), 134.9 (*C*HAr), 131.1 (*C*Ar), 123.6 (*C*HAr), 71.7 (*C*H), 52.3 (*C*H_3_), 51.7 (*C*H_3_), 30.1 (*C*H_2_), 28.2 (*C*H_2_). MS (EI) *m*/*z*: 264 (M^+^, 7%), 205 (74), 204 (43), 191 (26), 145 (100), 118 (27), 105 (38). HRMS (ESI): *m*/*z* calcd for C_13_H_16_N_2_O_4_ [M^+^] 264.111; found: 264.1108.

5-(*Tert*-butyl) 1-methyl (*E*)-2-[(pyridin-3-ylmethylene)amino]pentanedioate (**2k**): Pale yellow oil (241.7 mg, 83%, 89% purity). IR (neat) ν_max_: 1727, 1369, 1253, 1153, 802, 709 cm^−1^. ^1^H NMR (300 MHz) δ 8.88–8.86 (m, 1H, ArH), 8.66 (dd, *J* = 4.8, 1.7 Hz, 1H, ArH), 8.32 (s, 1H, *H*C=N), 8.16 (dt, *J* = 7.9, 1.9 Hz, 1H, ArH), 7.34 (dd, *J* = 7.7, 4.8 Hz, 1H, ArH), 4.13–4. 06 (m, 1H, N*H*CO_2_Me), 3.74 (s, 3H, CH_3_), 2.38–2.10 (m, 4H, C*H*_2_-C*H*_2_), 1.42 (s, 9H, [(CH_3_)_3_]. ^13^C NMR (75 MHz) δ 171.9 (*C*=O), 171.67(*C*=O), 161.3 (*C*=N), 152.0 (*C*HAr), 150.6 (*C*HAr), 134.9 (*C*HAr), 131.1 (*C*Ar), 123.6 (*C*HAr), 80.5 [*C*(CH_3_)_3_], 71.8 (*C*H), 52.2 (CO_2_*C*H_3_), 31.4 (*C*H_2_), 28.3 (*C*H_2_), 28.0 [C(*C*H_3_)_3_]. MS (EI) *m*/*z*: 306 (M^+^, 1%), 250 (69), 233 (38), 191 (100), 145 (54), 118 (28), 105 (22), 57 (33). HRMS (ESI): *m*/*z* calcd for C_16_H_22_N_2_O_4_ [M^+^] 306.158; found: 306.1579.

5-(*Tert*-butyl) 1-methyl (*E*)-2-[(thien-2-ylmethylene)amino]pentanedioate (**2l**): Pale yellow oil (147.2 mg, 90%, 91% purity). IR (neat) ν_max_: 1727, 1627, 1249, 1211, 1153, 713 cm^−1^. ^1^H NMR (400 MHz) δ 8.39 (s, 1H, *H*C=N), 7.46 (d, *J* = 5.0 Hz, 1H, Thienyl-*H*), 7.38 (dd, *J* = 3.6, 1.0 Hz, 1H, Thienyl-*H*), 7.10 (dd, *J* = 4.9, 3.7 Hz, 1H, Thienyl-*H*), 4.04 (dd, *J* = 8.5, 4.1 Hz, 1H, NC*H*CO_2_Me), 3.75 (s, 3H, CO_2_C*H*_3_), 2.43–2.16 (m, 4H, C*H*_2_-C*H*_2_), 1.45 [s, 9H, C(C*H*_3_)_3_]. ^13^C NMR (101 MHz) δ 172.1, 171.9 (C=O), 157.1 (*C*=N), 141.6 (S*C*C=N), 131.6, 129.8, 127.4 (Ar*C*), 80.4 [*C*(CH_3_)_3_], 71.4 (N*C*H), 52.2 (*C*H_3_), 31.6, 28.3 (*C*H_2_), 28.1 [C(*C*H_3_)_3_]. MS (EI) *m*/*z*: 311 (M^+^, 6%), 255 (93), 238 (64), 195 (100), 194 (95), 150 (71), 123 (35), 112 (44), 110 (29), 96 (44), 57 (51), 43 (36), 41 (23). HRMS (ESI): *m*/*z* calcd for C_15_H_21_NO_4_S[M^+^] 311.1196; found: 311.119.

5-Butyl 1-methyl (*E*)-2-(benzylideneamino)-2-methylpentanedioate (**2m**): Pale yellow oil (152 mg, 95%, 86% purity). IR (neat) v_max_: 1729, 1643, 1452, 1378, 1174, 1114, 730, 694 cm^−1^. ^1^H NMR (400 MHz) δ 8.24 (s, 1H, *H*C=N), 7.76–7.71 (m, 2H, ArH), 7.40–7.36 (m, 3H ArH), 4.02 (m, *J* = 6.7, 0.8 Hz, 2H, CO_2_C*H*_2_CH_2_CH_2_CH_3_), 3.71 (s, 3H, CO_2_CH_3_), 2.45 (m, *J* = 9.6, 7.9, 5.7 Hz, 2H, NCH_3_CH_2_C*H*_2_), 2.33 (m, *J* = 13.7, 10.1, 5.6 Hz, 1H, NCH_3_C*H*_2_CH_2_), 2.19–2.10 (m, 1H, NCH_3_C*H*_2_CH_2_), 1.55 (m, *J* = 8.2, 7.0, 6.0 Hz, 2H, CO_2_CH_2_C*H*_2_CH_2_CH_3_), 1.49 (s, 3H, NCC*H*_3_), 1.39–1.26 (m, 2H, CO_2_CH_2_CH_2_C*H*_2_CH_3_), 0.89 (t, *J* = 7.4 Hz, 3H, CO_2_CH_2_CH_2_CH_2_C*H*_3_). ^13^C NMR (101 MHz) δ 174.14 (*C*=O), 173.53 (C=O), 159.81 (*C*=N), 136.27 (*C*Ar), 130.97 (*C*HAr), 128.53 (*C*HAr x2), 128.35 (*C*HAr x2), 67.52 (*C*H), 64.32 (*C*H_2_), 52.21 (*C*H_3_), 35.06 (*C*H_2_), 30.64 (*C*H_2_), 29.64 (*C*H_2_), 23.40 (*C*H_3_), 19.11 (*C*H_2_), 13.69(*C*H_3_). MS (EI) *m*/*z*: 319 (M+, >1%), 260 (100), 158 (38). HRMS (ESI): *m*/*z* calcd for C_17_H_22_NO_4_ [M^+^–CH_3_] 304.1549; found: 304.1545.

Dimethyl (*E*)-2-[(4-bromobenzylidene)amino]-2-methylpentanedioate (**2n**): Pale yellow oil (110.2 mg, 90%, 90% purity). IR (neat) ν_max_: 1731, 1438, 1245, 1172, 1114, 821 cm^−1^. ^1^H NMR (300 MHz) δ 8.22 (s, 1H, *H*C=N), 7.65–7.59 (m, 2H, ArH), 7.57–7.51 (m, 2H, ArH), 3.74 (s, 3H, CH_3_), 3.64 (s, 3H, CH_3_), 2.58–2.26 (m, 4H, C*H*_2_-C*H*_2_), 1.50 (s, 3H, C*H*_3_). ^13^C NMR (101 MHz) δ 173.9 (*C*=O), 173.8 (*C*=O), 158.7 (*C*=N), 135.1 (*C*Ar), 131.8 (2x*C*HAr), 129.78 (2x*C*HAr), 125.4 (Br*C*Ar), 67.5 (N*C*CO_2_Me), 52.3 (*C*H_3_), 51.6 (*C*H_3_), 35.0 (*C*H_2_), 29.4 (*C*H_2_), 23.3 (*C*H_3_CN). MS (EI) *m*/*z*: 356 (M^+^, <1%), 298 (97), 296 (100), 236 (27), 184 (19), 89 (32). HRMS (ESI): *m*/*z* calc for C_13_H_15_BrNO_2_ [M^+^–CO_2_CH_3_] 296.0286; found: 296.0286.

5-Butyl 1-methyl (*E*)-2-[(4-bromobenzylidene)amino]-2-methylpentanedioate (**2o**): Pale yellow oil (191 mg, 96%, 91% purity). IR (neat) V_max_: 1729, 1643, 1438, 1170, 1114, 1066, 821, 744 cm^−1^. ^1^H NMR (400 MHz) δ 8.18 (s, 1H, N*H*), 7.58 (ddd, *J* = 8.4, 5.8, 2.7 Hz, 2H, ArH), 7.49 (dt, *J* = 12.4, 4.7 Hz, 2H, ArH), 4.00 (td, *J* = 6.7, 2.9 Hz, 2H, CO_2_C*H*_2_CH_2_CH_2_CH_3_), 3.68 (s, 3H C*H*_3_), 2.41 (dqt, *J* = 9.1, 6.5, 3.2 Hz, 2H, C*H*_2_CO_2_^n^Bu), 2.36–2.23 (m, 1H, NCC*H*_2_), 2.12 (dddd, *J* = 12.0, 8.3, 6.0, 2.3 Hz, 1H, NCC*H*_2_), 1.53 (dt, *J* = 12.7, 6.8 Hz, 2H, CO_2_CH_2_C*H*_2_CH_2_CH_3_), 1.45 (s, 3H, C*H*_3_), 1.39–1.24 (m, 2H, CO_2_CH_2_CH_2_C*H*_2_CH_3_), 0.87 (tt, *J* = 6.2, 3.2 Hz, 3H, C*H*_3_).^13^C NMR (101 MHz) δ 173.86 (*C*=O), 173.38 (*C*=O), 158.61 (H*C*=N), 135.16 (*C*Ar), 131.73 (*C*HAr x2), 129.76 (*C*HAr x2), 125.37 (*C*Ar), 67.60 (C), 64.32 (*C*H_2_), 52.24 (*C*H_3_), 35.01 (*C*H_2_), 30.62 (*C*H_2_), 29.62 (*C*H_2_), 23.36 (*C*H_3_), 19.10 (*C*H_2_), 13.68 (*C*H_3_)). MS (EI) *m*/*z*: 398 (M+, >1%), 338 (100), 238 (31). HRMS (ESI): *m*/*z* calcd for C_17_H_21_BrNO_4_ [M^+^–CH_3_] 382.0654; found: 382.0647.

5-(*Tert*-butyl) 1-methyl (*E*)-2-[(4-bromobenzylidene)amino]-2-methylpentanedioate (**2p**): Pale yellow oil (104.5 mg, 77%, 88% purity). IR (neat) ν_max_: 2981, 1727, 1643, 1589, 1249, 1153, 1114, 821 cm^−1^. ^1^H NMR (400 MHz) δ 8.20 (s, 1H, *H*C=N), 7.64–7.59 (m, 2H, ArH), 7.55–7.50 (m, 2H, ArH), 3.72 (s, 3H, CH_3_), 2.45–2.34 (m, 2H, C*H*_2_), 2.32–2.22 (m, 2H, C*H*_2_), 1.48 (s, 3H, CH_3_), 1.42 [s, 9H, C(CH_3_)_3_]. ^13^C NMR (75 MHz) δ 174.0 (*C*=O), 172.6 (*C*=O), 158.5 (C=N), 135.2 (*C*Ar), 131.7 (2x*C*HAr), 129.8 (2x*C*HAr), 125.3 (Br*C*Ar), 80.3 (*C*(CH_3_)_3_), 67.6 (N*C*CO_2_Me), 52.2 (CO_2_*C*H_3_), 34.9 (*C*H_2_), 30.7 (*C*H_2_), 28.0 [C(*C*H_3_)_3_], 23.3 (*C*H_3_). MS (EI) *m*/*z*: 398 (M^+^, <1%), 284 (97), 282 (100), 268 (100), 238 (20), 236 (20), 184 (20), 160 (48), 89 (35), 57 (31), 43(24). HRMS (ESI): *m*/*z* calc for C_12_H_13_BrNO_2_ [M^+^-C_6_H_11_O_2_] 284.0102; found: 284.0102.

Dimethyl (*E*)-2-benzyl-2-[(4-bromobenzylidene)amino]pentanedioate (**2q**): Pale yellow oil (201.2 mg, 90%, 90% purity). IR (neat) ν_max_: 1731, 1438, 1172, 1083, 821, 740, 701 cm^−1^. ^1^H NMR (300 MHz) δ 7.97 (s, 1H, *H*C=N), 7.60–7.51 (m, 4H, ArH), 7.24–7.14 (m, 5H, ArH), 3.74 (s, 3H, CH_3_), 3.62 (s, 3H, CH_3_), 3.35–3.10 (m, 2H, C*H*_2_Ar), 2.45–2.23 (m, 4H, C*H*_2_-C*H*_2_). ^13^C NMR (75 MHz) δ 173.7 (*C*=O), 172.8 (*C*=O), 160.5 (*C*=N), 135.8 (*C*Ar), 135.1 (*C*ArCH_2_), 131.8 (2x*C*HAr), 130.6 (2x*C*HAr), 129.7 (2x*C*HAr), 128.1 (2x*C*HAr), 126.9 (*C*HAr), 125.4 (Br*C*Ar), 71.6 (N*C*CO_2_Me), 52.0 (*C*H_3_), 51.6 (*C*H_3_), 44.3 (Ph*C*H_2_), 32.9 (*C*H_2_), 29.5 (*C*H_2_). MS (EI) *m*/*z*: 432 (M^+^, <1%), 372 (18), 342 (98), 340 (100), 254 (25), 91 (45). HRMS (ESI): *m*/z calc for C_19_H_19_BrNO_2_ [M^+^–CO_2_CH_3_] 372.0599; found: 372.0605.

Methyl (*E*)-2-[(4-bromobenzylidene)amino]-5-(dimethylamino)-5-oxopentanoate (**2u**): Pale yellow oil (153.1 mg 90%, 86% purity). IR (neat) ν_max_: 1735, 1639, 1203, 1168, 1064, 821 cm^−1^. ^1^H NMR (300 MHz) δ 8.24 (s, 1H, *H*C=N), 7.65–7.59 (m, 2H, ArH), 7.55–7.49 (m, 2H, ArH), 4.19–4.13 (m, 1H, NC*H*CO_2_Me), 3.71 (s, 3H, CO_2_CH_3_), 2.93 (s, 3H, NCH_3_), 2.89 (s, 3H, NCH_3_), 2.40–2.29 (m, 4H, C*H*_2_-C*H*_2_). ^13^C NMR (101 MHz) δ 172.1 (*C*=O), 171.9 (*C*=O), 162.9 (*C*H=N), 134.5 (*C*Ar), 131.8 (CHAr), 129.9 (*C*HAr), 125.6 (Br*C*Ar), 71.6 (N*C*HCO_2_Me), 52.2 (CO_2_*C*H_3_), 37.1 (N*C*H_3_), 35.9 (N*C*H_3_), 28.9 (*C*H_2_), 28.7 (*C*H_2_). MS (EI) *m*/*z*: 354 (M^+^, 5%), 270 (30), 268 (32), 224 (35), 222 (31), 173 (24), 100 (26), 89 (31), 87 (100), 72 (35), 45 (25). HRMS (ESI): *m*/*z* calc for C_15_H_19_BrN_2_O_3_ [M^+^] 354.0579; found: 354.0565.

Methyl (*E*)-2-[(4-bromobenzylidene)amino]-4-cyano-2-(2-cyanoethyl)butanoate (**6v**): Pale yellow oil (117.0 mg, 90%, 84% purity). IR (neat) ν_max_: 1727, 1369, 1149, 825 cm^−1^. ^1^H NMR (300 MHz) δ 8.24 (s, 1H, *H*C=N), 7.65–7.59 (m, 2H, ArH), 7.55–7.49 (m, 2H, ArH), 4.19–4.13 (m, 1H, NC*H*CO_2_Me), 3.71 (s, 3H, CO_2_CH_3_), 2.93 (s, 3H, NCH_3_), 2.89 (s, 3H, NCH_3_), 2.40–2.29 (m, 4H, C*H*_2_-C*H*_2_). ^13^C NMR (101 MHz) δ 172.1 (*C*=O), 171.9 (*C*=O), 162.9 (*C*H=N), 134.5 (*C*Ar), 131.8 (CHAr), 129.9 (*C*HAr), 125.7 (Br*C*Ar), 71.6 (N*C*HCO_2_Me), 52.2 (CO_2_*C*H_3_), 37.1 (N*C*H_3_), 35.3 (N*C*H_3_), 28.9 (*C*H_2_), 28.7 (*C*H_2_). MS (EI) *m*/*z*: 354 (M^+^, 5%), 270 (30), 268 (32), 224 (35), 222 (31), 173 (24), 100 (26), 89 (31), 87 (100), 72 (35), 45 (25). HRMS (ESI): *m*/*z* calc for C_15_H_19_BrN_2_O_3_ [M^+^] 354.0579; found: 354.0565.

### 3.3. General Procedure for the Synthesis of Pyroglutamate Derivatives

To a solution of NaBH_4_ (0.8 mmol, 2 eq) in Methanol (4 mL) at 0 °C, a solution of corresponding adduct **2** in Methanol (2mL) was added and the reaction was refluxed at 80 °C for 2 h. After that, the solvent was removed and the crude product was redissolved in AcEOt (4 mL) followed by the addition of SiO_2_ and refluxed again for 24 h. Then, the mixture was filtered and removed from the solvent under vacuum. Finally, the crude product was purified by flash column chromatography on silica gel (Hexane/AcEOt, 3:1) to afford the corresponding cycloadducts **8**.

Methyl 5-oxo-1-(pyridin-3-ylmethyl)pyrrolidine-2-carboxylate (**8a**): Pale yellow oil (93 mg, 60%). IR (neat) ν_max_: 1689, 1411, 1211, 1025, 794, 721 cm^−1^. ^1^H NMR (300 MHz) δ 8.48 (dd, *J* = 4.7, 1.2 Hz, 1H, ArH), 8.41 (d, *J* = 1.2 Hz, 1H, ArH), 7.54 (dt, *J* = 7.8, 2.0 Hz, 1H, ArH), 7.27–7.18 (m, 1H, ArH), 4.90 (d, *J* = 15.1 Hz, 1H, C*H*_2_NC=O), 4.04 (d, *J* =15.1 Hz, 1H, C*H*_2_NC=O), 3.94 (dd, *J* = 9.0, 3.2 Hz, 1H, NC*H*CO_2_Me), 3.62 (s, 3H, CH_3_), 2.56–2.45 (m, 1H, C*H*_2_), 2.43–2.34 (m, 1H, C*H*_2_), 2.27–2.17 (m, 1H, C*H_2_*), 2.11–1.99 (m, 1H, C*H*_2_). ^13^C NMR (101 MHz) δ 175.2 (N*C*=O), 171.9 (*C*=O), 149.6 (N*C*HAr), 149.3 (N*C*HAr), 136.3 (*C*HAr), 131.6 (*C*Ar), 123.7 (*C*HAr), 58.8 (N*C*HCO_2_), 52.6 (*C*H_3_), 43.2 (*C*H_2_N), 29.3 (*C*H_2_), 22.8 (*C*H_2_). MS (EI) *m*/*z*: 234 (M^+^, 14%), 175 (98), 92 (100), 65(17). HRMS (ESI): *m*/*z* calc for C_12_H_14_N_2_O_3_ [M^+^] 234.1004; found: 234.0998.

Methyl 1-(4-bromobenzyl)-2-methyl-5-oxopyrrolidine-2-carboxylate (**8b**): Pale yellow oil (49.1 mg, 42%). IR (neat) ν_max_: 2360, 1735, 1689, 1392, 1168 cm^−1^. ^1^H NMR (400 MHz) δ 7.44–7.38 (m, 2H, ArH), 7.19–7.11 (m, 2H, ArH), 4.52 (d, *J* = 15.6 Hz, 1H, ArC*H*_2_N), 4.29 (d, *J* = 15.5 Hz, 1H, ArC*H*_2_N), 3.51 (s, 3H, CO_2_CH_3_), 2.57 (dt, *J* = 17.0, 9.8 Hz, 1H, NC=OC*H*_2_), 2.46 (ddd, *J* = 17.0, 9.7, 2.7 Hz, 1H, NC=OC*H*_2_), 2.34 (ddd, *J* = 12.9, 9.3, 2.7 Hz, 1H, NCC*H*_2_), 1.90 (dt, *J* = 13.2, 9.9 Hz, 1H, NCC*H*_2_), 1.41 (s, 3H, C*H*_3_). ^13^C NMR (101 MHz) δ 175.8 (*C*=O), 173.7 (*C*=O), 136.6 (*C*Ar), 131.8 (2x*C*HAr), 129.9 (2xCHAr), 121.2 (Br*C*Ar), 66.0 (N*C*CH_3_), 52.4 (CO_2_*C*H_3_), 43.9 (Ar*C*H_2_N), 32.1 (C=O*C*H_2_), 29.5 (C*C*H_2_), 23.2 (NC*C*H_3_). MS (EI) *m*/*z*: 325 (M^+^, 7%), 268 (72), 266 (73), 171 (95), 169 (100), 90 (28), 89 (22). HRMS (ESI): *m*/*z* calc for C_14_H_16_BrNO_3_ [M^+^] 325.0314; found: 325.0306.

Methyl 2-benzyl-1-(4-bromobenzyl)-5-oxopyrrolidine-2-carboxylate (**8c**): Colorless needles (77 mg, 63%). Mp: 78–79 ºC (n-hexane/AcOEt). IR (neat) ν_max_: 1689, 1392, 1261, 1184, 1068, 806, 732, 701 cm^−1^. ^1^H NMR (300 MHz) δ 7.46–7.40 (m, 2H, ArH), 7.30–7.24 (m, 3H, ArH), 7.23–7.17 (m, 2H, ArH), 7.10–7.04 (m, 2H, ArH), 4.81 (d, *J* = 15.5 Hz, 1H, *H*CN), 4.40 (d, *J* = 15.5 Hz, 1H, *H*CN), 3.38 (s, 3H, CH_3_), 3.27 (d, *J* = 14.0 Hz, 1H, NCC*H*Ph), 3.00 (d, *J* = 14.0 Hz, 1H, NCC*H*Ph), 2.46–2.23 (m, 2H, NCOCH_2_), 2.10–1.84 (m, 2H, NCCH_2_). ^13^C NMR (75 MHz) δ 176.0 (*C*=O), 172.2 (*C*=O), 136.5 (*C*Ar), 134.4 (*C*Ar), 131.4 (2x*C*HAr), 130.0 (2x*C*HAr), 129.9 (2x*C*HAr), 128.6 (2x*C*HAr), 127.3 (*C*HAr), 121.2 (Br*C*Ar), 69.2 (N*C*CO_2_Me), 52.2 (*C*H_3_), 44.0 (Ph*C*H_2_N), 40.4 (C*C*H_2_Ph), 29.2 (*C*H_2_C=O), 27.5 (NC*C*H_2_). MS (EI) *m*/*z*: 402 (M^+^, 1%), 312 (67), 310 (68), 171 (97), 169 (100), 91 (22), 90 (27). HRMS (ESI): *m*/*z* calc for C_20_H_20_BrNO_3_ [M^+^] 401.0627; found: 401.0619.

Methyl 2-[(1*H*-indol-3-yl)methyl]-1-(4-bromobenzyl)-5-oxopyrrolidine-2-carboxylate (**8d**): Purple oil (105.6 mg, 45%). IR (neat) v_max_: 1735, 1673, 1434, 1394, 1257, 1070, 736 cm^−1^. ^1^H NMR (400 MHz) δ 8.29 (s, 1H, NH), 7.56 (dt, *J* = 7.7, 1.0 Hz, 1H, ArH), 7.46–7.41 (m, 2H, ArH), 7.35 (dt, *J* = 8.1, 1.0 Hz, 1H, ArH), 7.27–7.11 (m, 4H, ArH), 6.77 (s, 1H, C*H*NH), 4.85 (d, *J* = 15.3 Hz, 1H, *H_2_*CN), 4.36 (d, *J* = 15.2 Hz, 1H, *H_2_*CN), 3.52 (d, *J* = 15.2 Hz, 1H, C*H*_2_CCHNH), 3.37 (s, 3H, CO_2_C*H_3_*), 3.18 (d, *J* = 15.3 Hz, 1H, C*H*_2_CCHNH), 2.44 (ddd, *J* = 16.2, 9.8, 5.5 Hz, 1H, NCOC*H*_2_), 2.30–2.20 (m, 1H, NCOCH_2_C*H*_2_), 2.13–1.97 (m, 2H, NCOC*H*_2_, NCOCH_2_C*H*_2_). ^13^C NMR (101 MHz) δ 176.7 (*C*=O), 173.1 (*C*=O), 136.4 (*C*Ar), 135.9 (*C*Ar), 131.6 (*C*HAr), 130.4 (*C*HAr), 128.4 (*C*Ar), 123.2 (*C*HAr), 122.4 (*C*HAr), 121.5 (*C*Ar), 119.9 (*C*HAr), 118.9 (*C*HAr), 111.5 (*C*HAr), 108.5 (*C*Ar), 69.7 (*C*), 52.3 (*C*H_3_), 44.2 (*C*H_2_), 29.7 (*C*H_2_), 29.6 (*C*H_2_), 27.9 (*C*H_2_). MS (EI) *m*/*z*: 441 (M+, >1%), 310 (21), 252 (26), 168 (100), 126 (20), 90 (26). HRMS (ESI): *m*/*z* calcd for C_22_H_21_BrN_2_O_3_ [M^+^] 440.0736; found: 440.0712.

Methyl 2-[(benzyloxy)methyl]-1-(4-bromobenzyl)-5-oxopyrrolidine-2-carboxylate (**8e**): Colorless liquid (107.2 mg, 46%) IR (neat) v_max_: 1739, 1693, 1432, 1392, 1166, 1070, 736, 698 cm^−1^. ^1^H NMR (400 MHz) δ 7.57–7.23 (m, 5H, ArH), 7.23–7.01 (m, 4H, ArH), 4.59 (d, *J* = 15.5 Hz, 1H, H_2_CN), 4.34 (d, *J* = 15.5 Hz, 1H, H_2_CN), 4.23 (q, *J* = 12.0 Hz, 2H, CCH_2_OC*H_2_*Ph), 3.68 (d, *J* = 10.0 Hz, 1H, CC*H*_2_OCH_2_Ph), 3.58 (s, 3H, CO_2_CH_3_), 3.46 (d, *J* = 10.0 Hz, 1H, CC*H*_2_OCH_2_Ph), 2.55–2.45 (m, 2H, C*H_2_*C=O), 2.20 (m, *J* = 13.2, 7.3, 5.8 Hz, 1H, C*H_2_*CCO_2_Me), 2.10 (m, *J* = 13.1, 9.5 Hz, 1H C*H_2_*CCO_2_Me). ^13^C NMR (101 MHz) δ 176.3 (*C*=O), 172.3 (*C*=O), 137.3 (*C*Ar), 137.1 (*C*Ar), 131.3 (*C*HAr), 129.9 (*C*HAr), 128.5 (*C*HAr), 128.0 (*C*HAr), 127.7 (*C*HAr), 121.0 (*C*), 73.4 (*C*H_2_), 71.2 (*C*H_2_), 69.6 (*C*), 52.6 (*C*H_3_), 44.7 (*C*H_2_), 29.3 (*C*H_2_), 27.1 (*C*H_2_). MS (EI) *m*/*z*: 432 (M+, 2%), 310 (66), 169 (100), 91 (73). HRMS (ESI): *m*/*z* calcd for C_21_H_22_BrNO_4_ [M^+^] 431.0732; found: 433.0723.

Methyl 1-(4-bromobenzyl)-2-[2-(methylthio)ethyl]-5-oxopyrrolidine-2-carboxylate (**8f**): Pale yellow oil (65.8 mg, 42%). IR (neat) v_max_: 1733, 1689, 1432, 1390, 1160, 1070, 732 cm^−1^. ^1^H NMR (400 MHz) δ 7.41 (d, *J* = 8.4 Hz, 2H, ArH), 7.16 (d, *J* = 8.4 Hz, 2H ArH), 4.51–4.29 (m, 2H, *H_2_*CN), 3.45 (s, 3H, CO_2_CH_3_), 2.61 (dt, *J* = 17.0, 9.6 Hz, 1H, CC*H*_2_CH_2_SCH_3_), 2.53–2.36 (m, 2H, CC*H*_2_CH_2_SCH_3_, CCH_2_C*H*_2_SCH_3_), 2.35–2.25 (m, 2H, CCH_2_C*H*_2_SCH_3_, NCC*H*_2_CH_2_CO), 2.23–2.10 (m, 1H, NCCH_2_C*H*_2_CO), 2.01 (s, 3H, SC*H*_3_), 1.98–1.87 (m, 2H, NCC*H*_2_CH_2_CO, NCCH_2_C*H*_2_CO). ^13^C NMR (101 MHz) δ 176.1 (*C*=O), 172.8 (*C*=O), 136.2 (*C*Ar), 131.6 (*C*HAr x 2), 130.3 (*C*HAr x 2), 121.6 (*C*Ar), 68.5 (*C*), 52.5 (*C*H_3_), 44.1 (*C*H_2_), 35.0 (*C*H_2_), 29.6 (*C*H_2_), 28.0 (*C*H_2_), 27.9 (*C*H_2_), 15.7 (*C*H_3_). MS (EI) *m*/*z*: 386 (M+, 13%), 328 (64), 169 (100), 90 (23), 61 (30). HRMS (ESI): *m*/*z* calcd for C_16_H_20_BrNO_3_S [M^+^] 385.0347; found: 387.0328.

### 3.4. Procedure to Obtain the Glutamic Acid 1,5-Dimethyl Ester Hydrochloride 9 [70]

To a solution of **2a** (1 eq, 0.33mmol) in Et_2_O (0.7 mL), 2M HCl/Et_2_O (0.35 mL) was added and stirred until a precipitate was observed, then the solvent was removed under vacuum. The crude product was purified, washed with Et_2_O (3 times), and the supernatant was removed. The remaining solid was characterized (62 mg, 90%). Mp 91–92 °C (89–91 °C) [70]. ^1^H NMR (300 MHz, Methanol-*d*_4_) δ 4.12 (t, *J* = 6.8 Hz, 1H), 3.84 (s, 3H), 3.70 (s, 3H), 2.58 (td, *J* = 7.3, 2.1 Hz, 2H), 2.30–2.07 (m, 2H). ^13^C NMR (101 MHz, Methanol-*d*_4_) δ 172.6, 169.2, 52.3, 51.8, 51.0, 28.7, 25.1.

## 4. Conclusions

The 1,4-addition of imino esters, derived from the condensation of amino esters and aldehydes, was a complicated task due to the formation of three secondary byproducts. The use of dimethylphenylphosphine as an organocatalyst was crucial for the control of the desired reaction, circumventing all of the undesired molecules and favoring the α-alkylated compound, which did not undergo the retro-Michael process. To the best of our knowledge, this is the first occasion that phosphines are involved in this particular transformation with the advantage of the minimization of the over-alkylation of the glycine template with alkyl acrylates. The reaction is very versatile because it tolerates many aromatic and heteroaromatic units bonded to the imino group, as well as substituents at the α-position of the imino ester. This methodology does not employ benzene as a solvent and the crude reaction materials were not impurified with variable amounts (10–25%) of the corresponding pyrrolidines (formed by 1,3-dipolar cycloadditions), such as occurred in a previous communication [16]. In addition, no sophisticated halogenated compounds were employed as electrophiles [15] and the presence of strong bases was avoided [17,18,19,20,21]. The access to different synthetic glutamates was ensured and the family of the corresponding pyroglutamate derivatives was successfully obtained by simple organic transformations.

## Figures and Tables

**Table 1:** Optimization conditions of the reaction shown in Scheme 2.

		Slow Addition		Products’ Ratio ^a^				
Entry	PR_3_ (10 mol%)	(min)	t (h)	2a	3a	4	5	1a
1	PPh_3_	---	15	0	9	0	0	91
2	dppe	---	15	37	22	0	4	37
3	dppe	---	48	22	32	0	30	16
4	dppe	---	72	56	39	0	5	0
5	dppe	**1a** (60)	72	80	0	20	0	0
6	dppe	MA (60)	72	88	0	0	12	0
7	dppe	MA (60)	12	71	0	0	9	20
8	Bu_3_P	---	72	0	0	0	100	0
9	Bu_3_P	MA (60)	72	0	0	100	0	0
10	Bu^t^_3_P	---	72	0	0	100	0	0
11	Me_2_PhP	---	12	23	0	38	38	0
12	Me_2_PhP	---	2	70	0	15	15	0
13	Me_2_PhP	MA (60)	2	70	5	0	5	20
14	Me_2_PhP	MA (60)	24	80	10	0	5	5
15	Me_2_PhP	MA (30)	24	97	0	0	0	3
16	Me_2_PhP	MA (5)	24	64	0	29	0	7
17	Me_2_PhP ^b^	MA (5)	24	50	0	0	0	50

^a^ The ratio was estimated according to integral values in crude product. ^1^H NMR and also using dimethyl terephthalate as the internal standard. ^b^ A 5 mol% of Me_2_PhP was added.

## Data Availability

Data are contained within the article and Supplementary Materials.

## Supplementary Materials

The following supporting information can be downloaded at: https://www.mdpi.com/article/10.3390/molecules29020342/s1, The NMR and representative FTIR copies.
