# Supplementary material for: Phosphine Catalyzed Michael-Type Additions: The Synthesis of Glutamic Acid Derivatives from Arylidene-α-amino Esters
Source: Molecules. 2024 Jan 10;29(2):342. doi: 10.3390/molecules29020342 (PMC10820836; doi:10.3390/molecules29020342)
Supplement: Supplementary file 1 [file molecules-29-00342-s001.zip › molecules-2795418-supplementary.pdf]

Phosphine Catalyzed Michael-Type Additions: The Synthesis of Glutamic Acid Derivatives from Arylidene-α-amino Esters †

Lesly V. Rodríguez-Flórez [1], María González-Marcos [1], Eduardo García-Mingüens [2], María de Gracia Retamosa [1], Misa Kawase [1], Elisabet Selva [2] and José M. Sansano [1,*]

[1] Departamento de Química Orgánica, Centro de Innovación en Química Avanzada (ORFEO-CINQA) and Instituto de Síntesis Orgánica, Universidad de Alicante, Ctra. Alicante-San Vicente s/n, 03080 Alicante, Spain

[2] Medalchemy, S. L. Ancha de Castelar, 46-48, entlo. A. San Vicente del Raspeig, 03690 Alicante, Spain

* Correspondence: jmsansano@ua.es

† Dedicated to the memory of Prof. David A. Evans.

TABLE OF CONTENTS

1. NMR COPIES………………………………………………………………. S1

2. REPRESENTATIVE FTIR COPIES ………………………………………….. S29

1. NMR COPIES

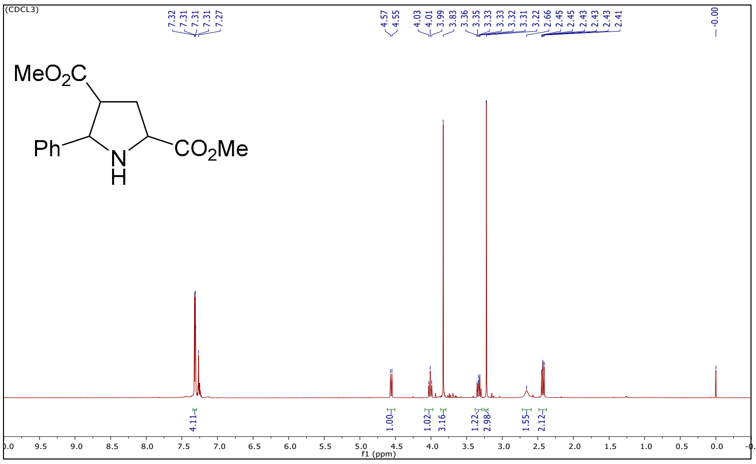

Figure S1, ^{1}H NMR of compound **3a**

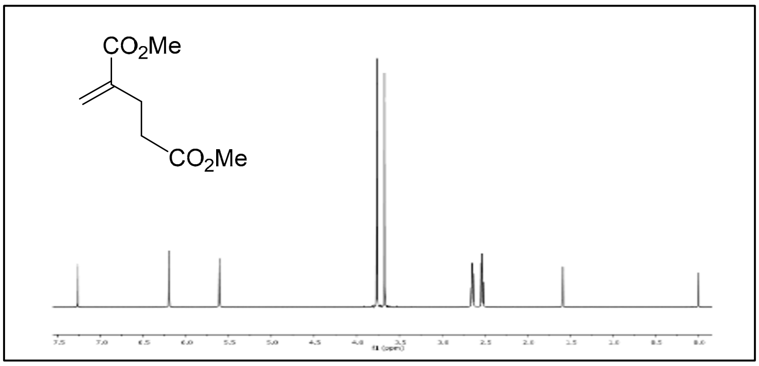

Figure S2, ^{1}H NMR of compound **4**

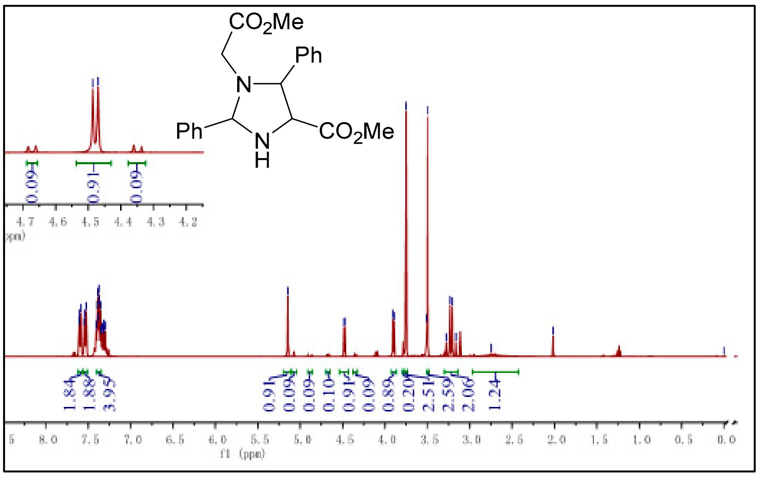

Figure S3, ^{1}H NMR of compound **5**

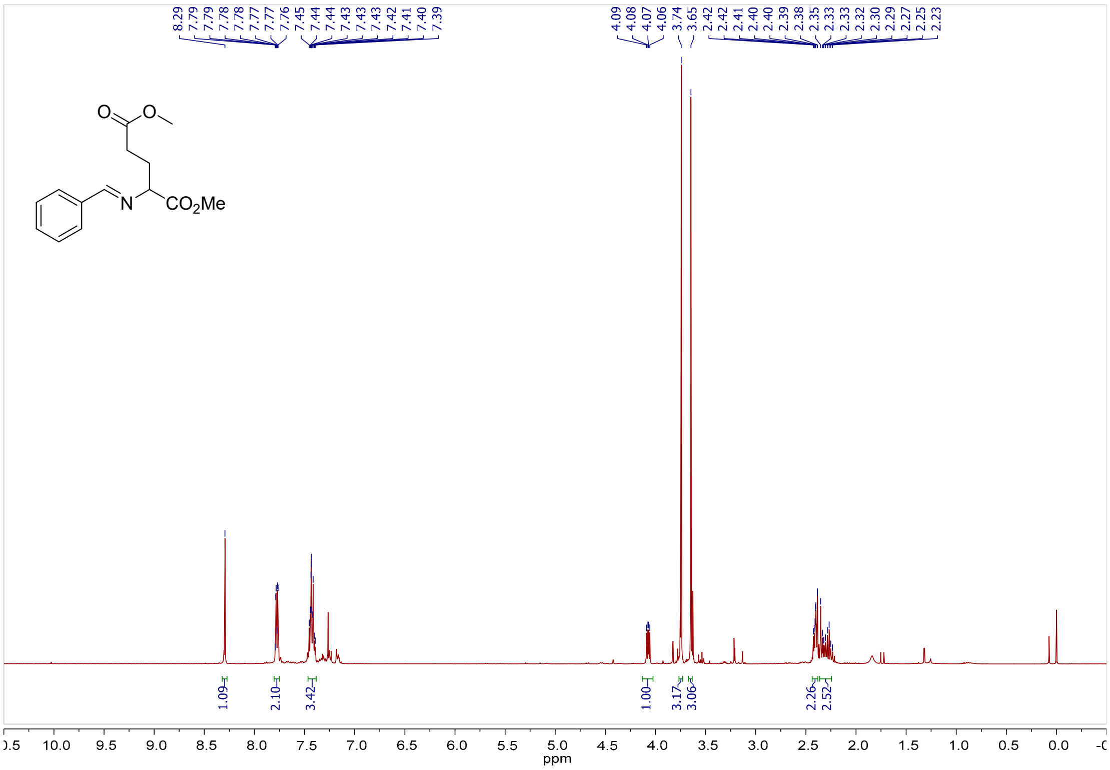

Figure S4, ^{1}H NMR of compound **2a**

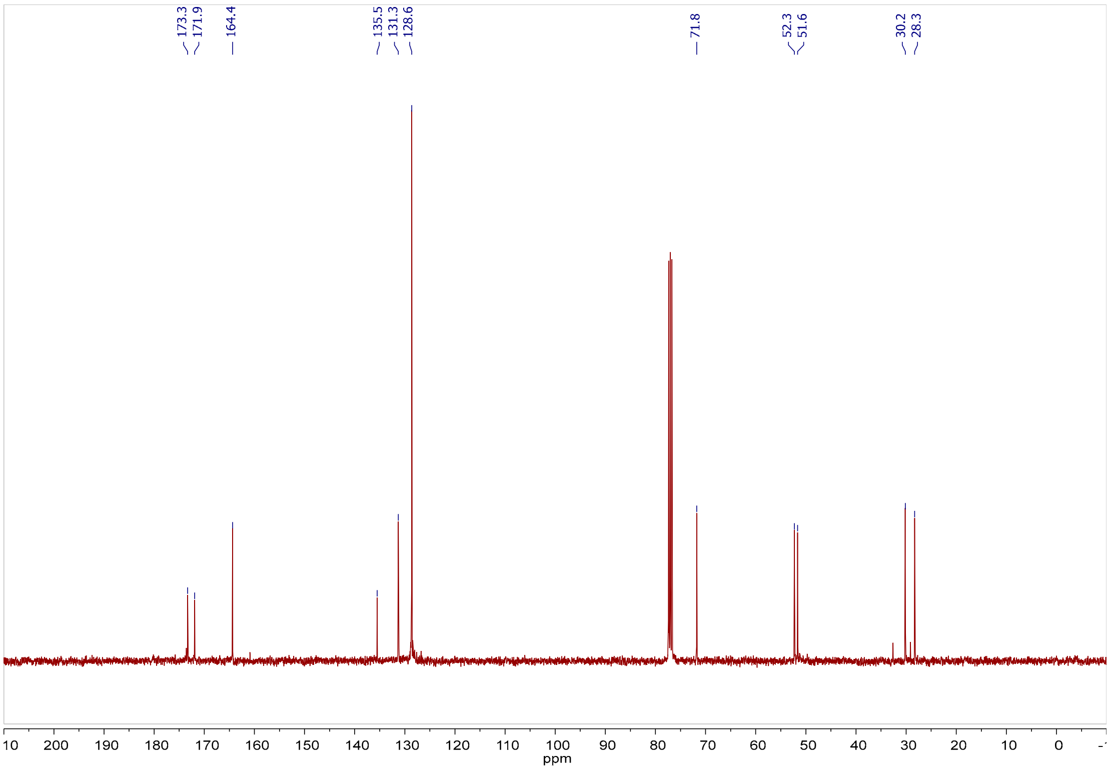

Figure S5, ^{13}C NMR of compound **2a**

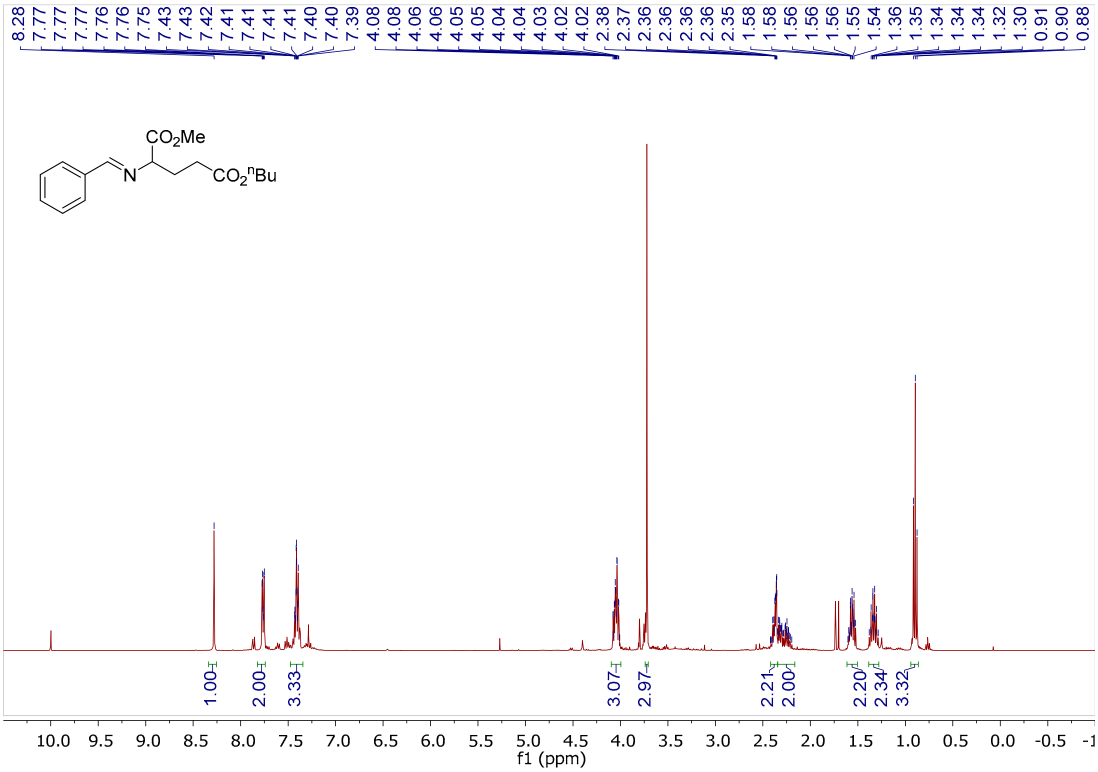

Figure S6, ^{1}H NMR of compound **2b**

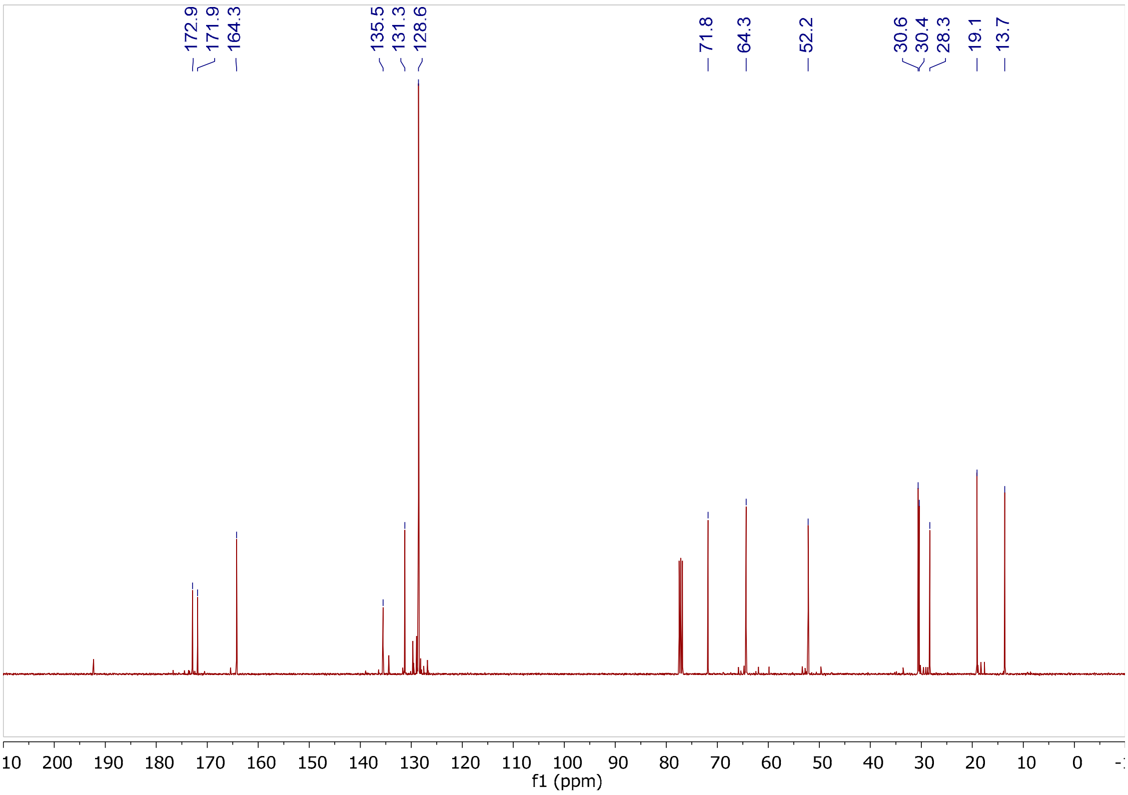

Figure S7, ^{13}C NMR of compound **2b**

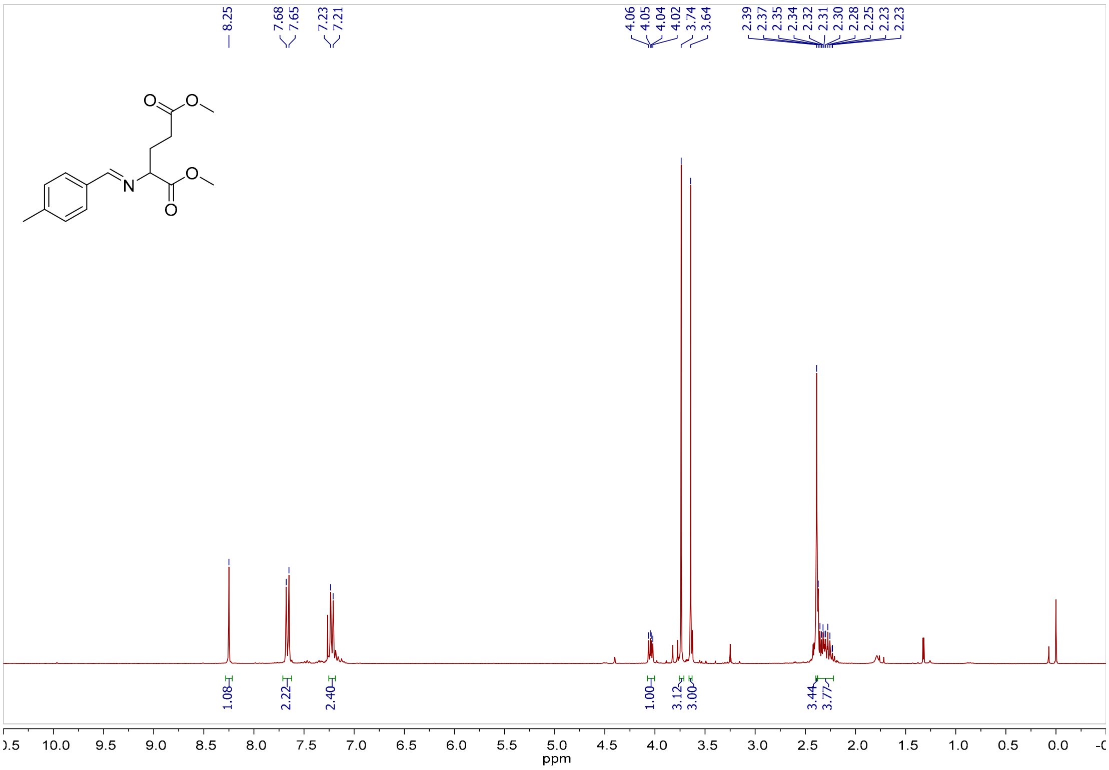

Figure S8, ^{1}H NMR of compound **2c**

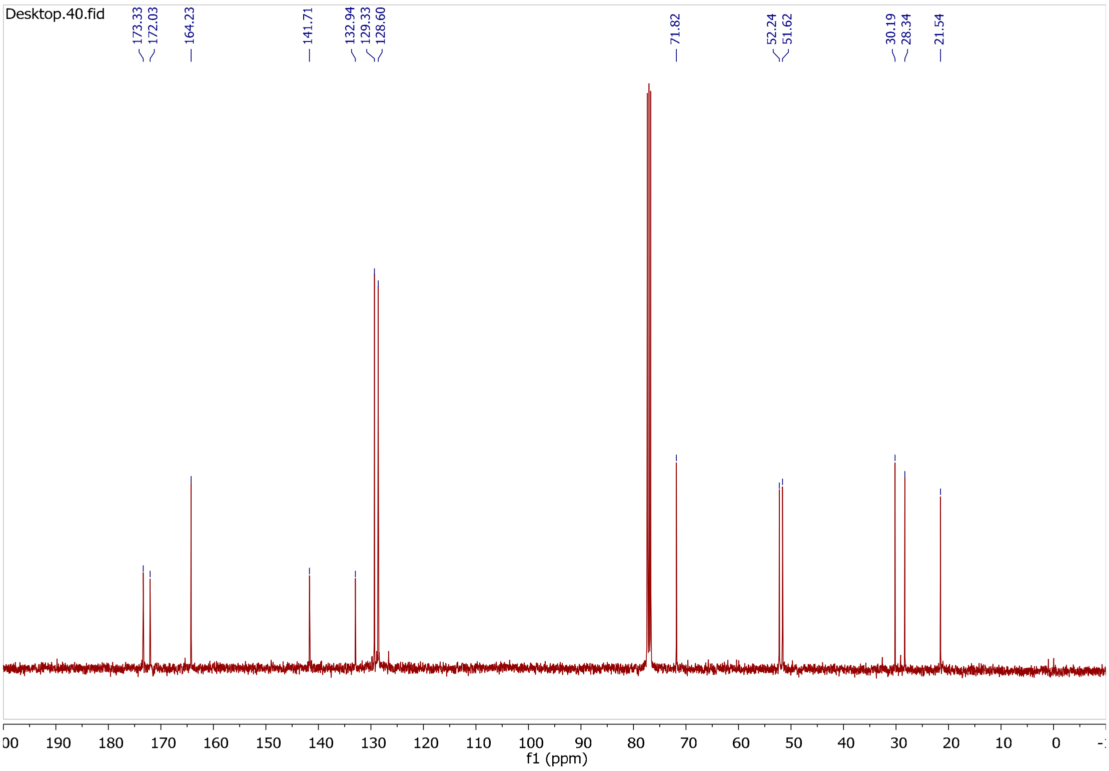

Figure S9, ^{13}C NMR of compound **2c**

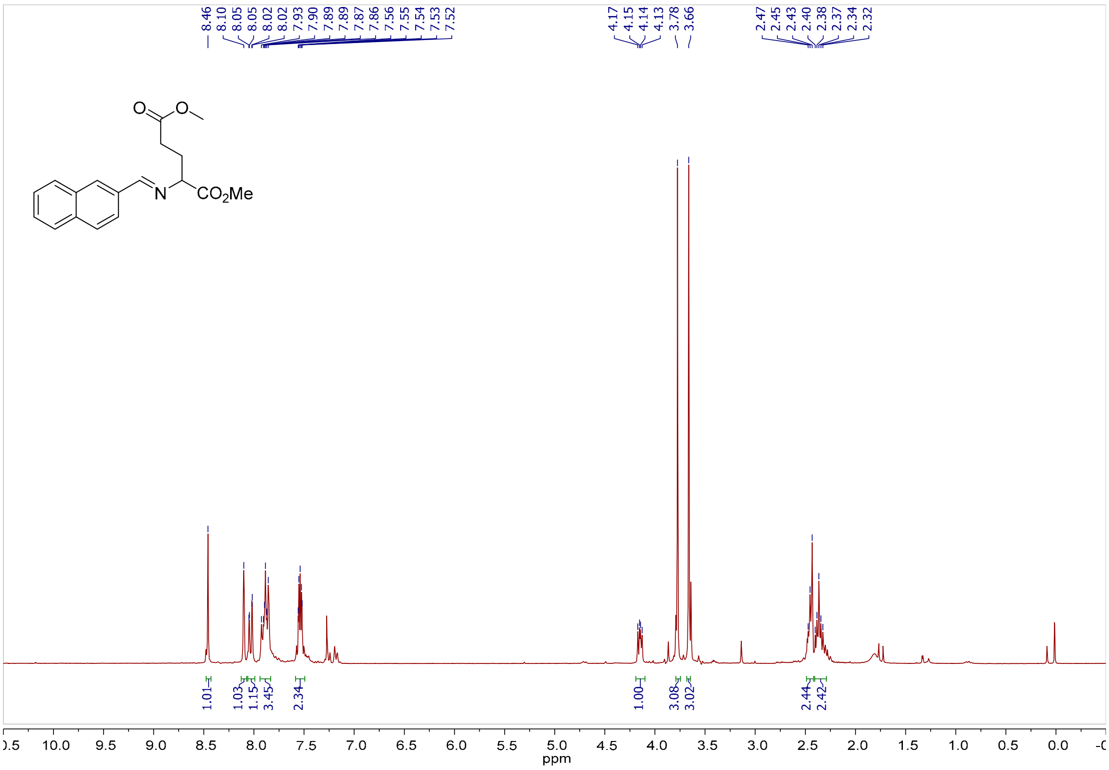

Figure S10, ^{1}H NMR of compound **2d**

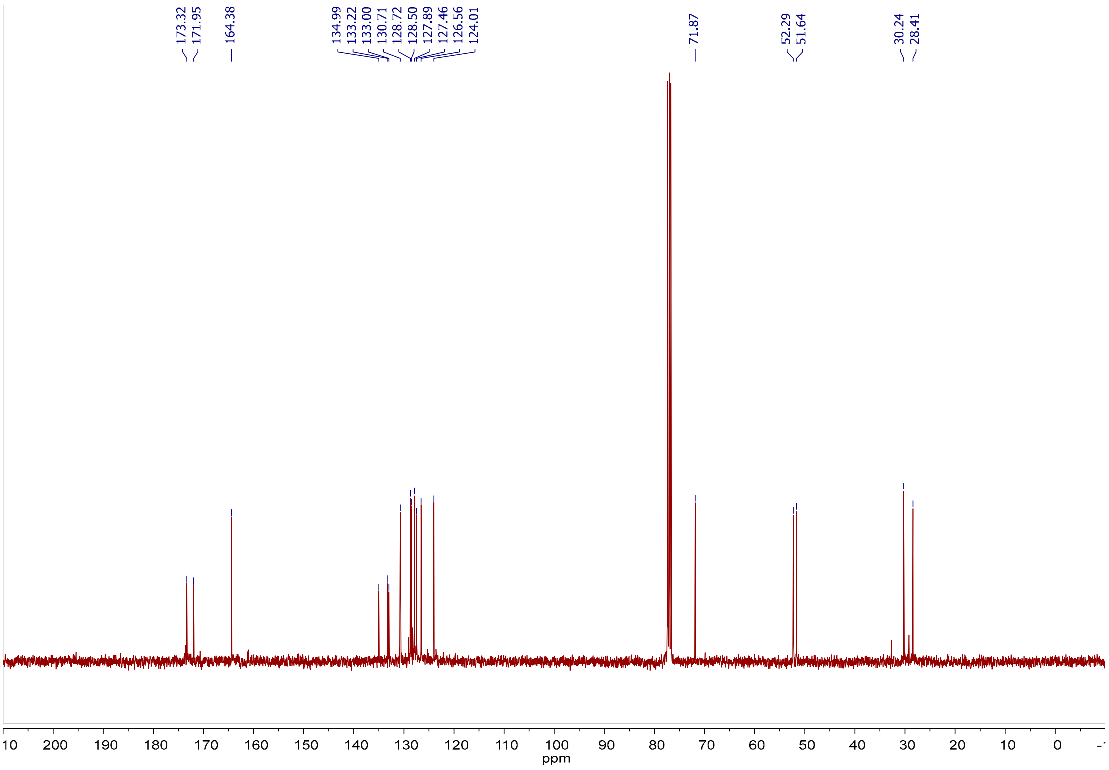

Figure S11, ^{13}C NMR of compound **2d**

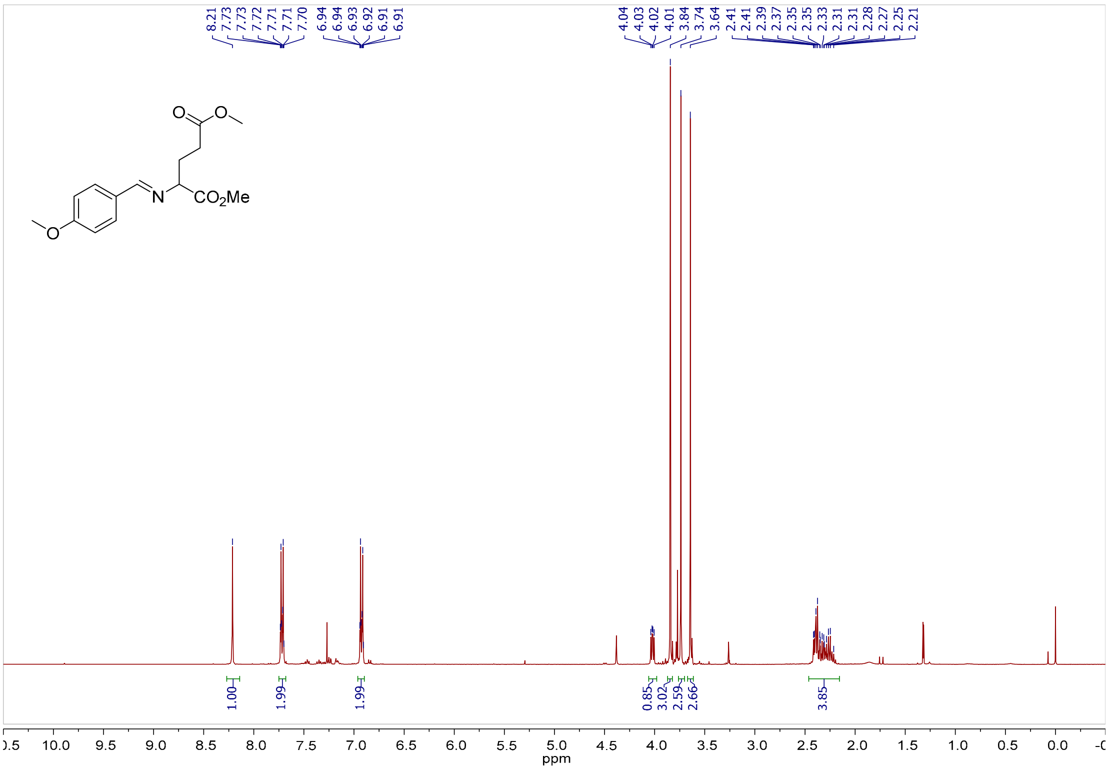

Figure S12, ^{1}H NMR of compound **2e**

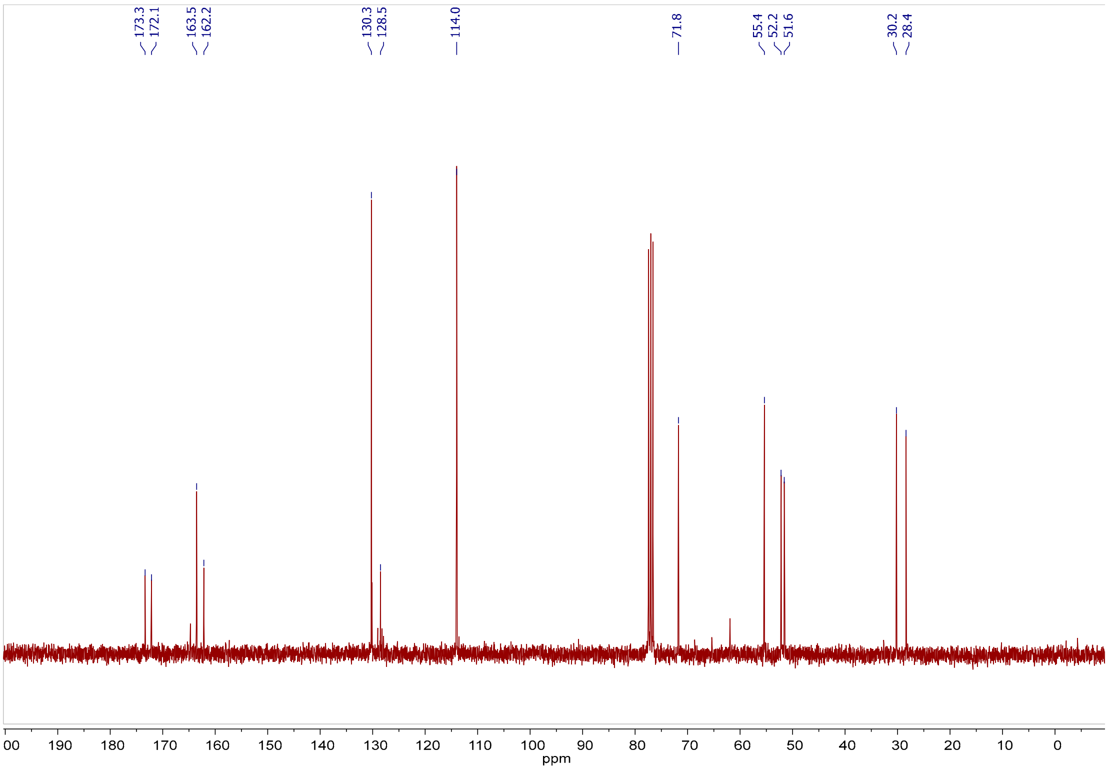

Figure S13, ^{13}C NMR of compound **2e**

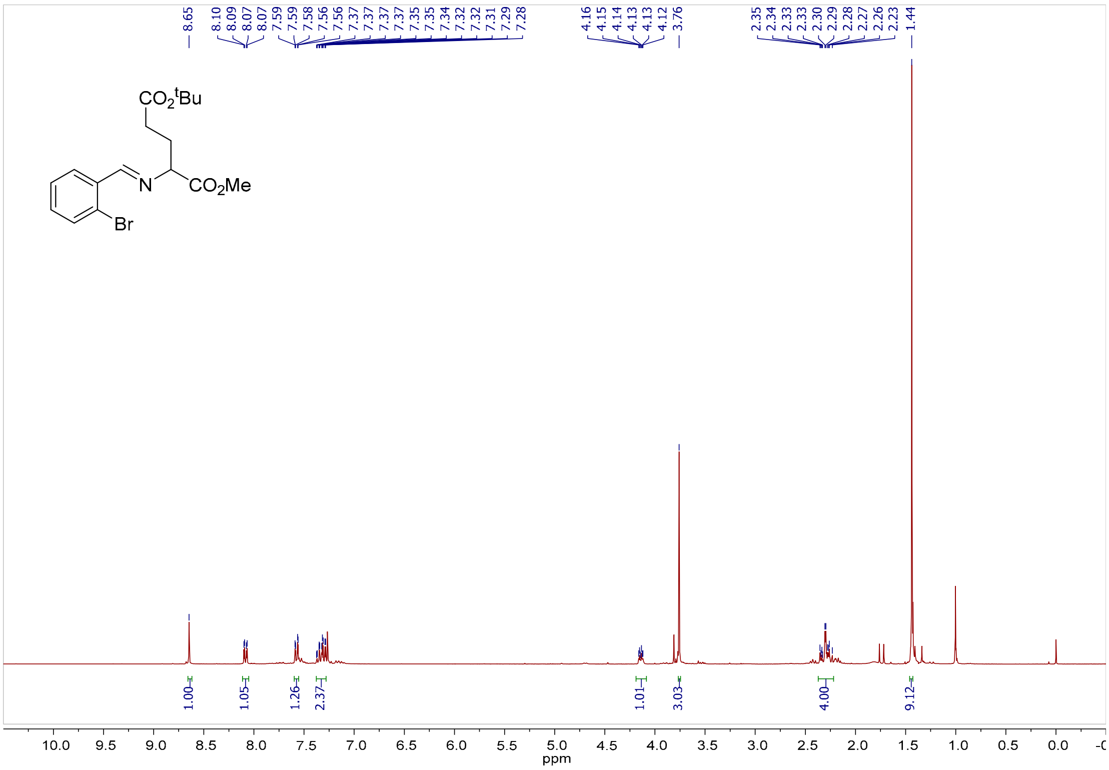

Figure S14, ^{1}H NMR of compound **2f**

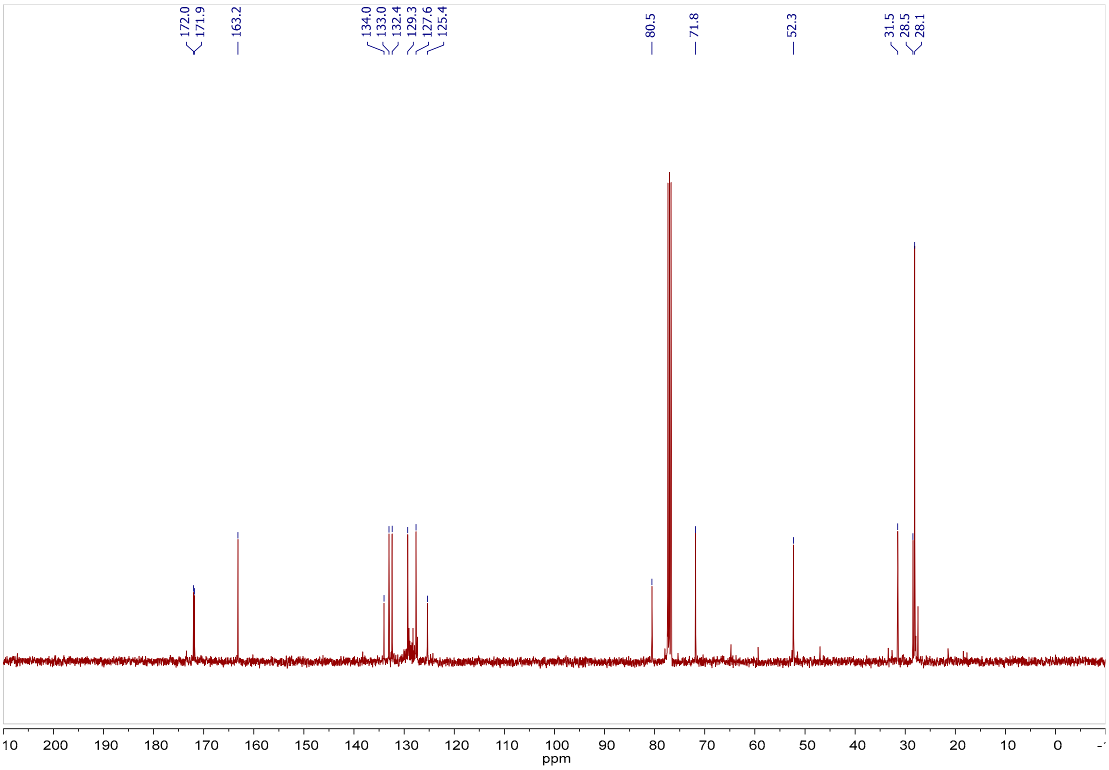

Figure S15, ^{13}C NMR of compound **2f**

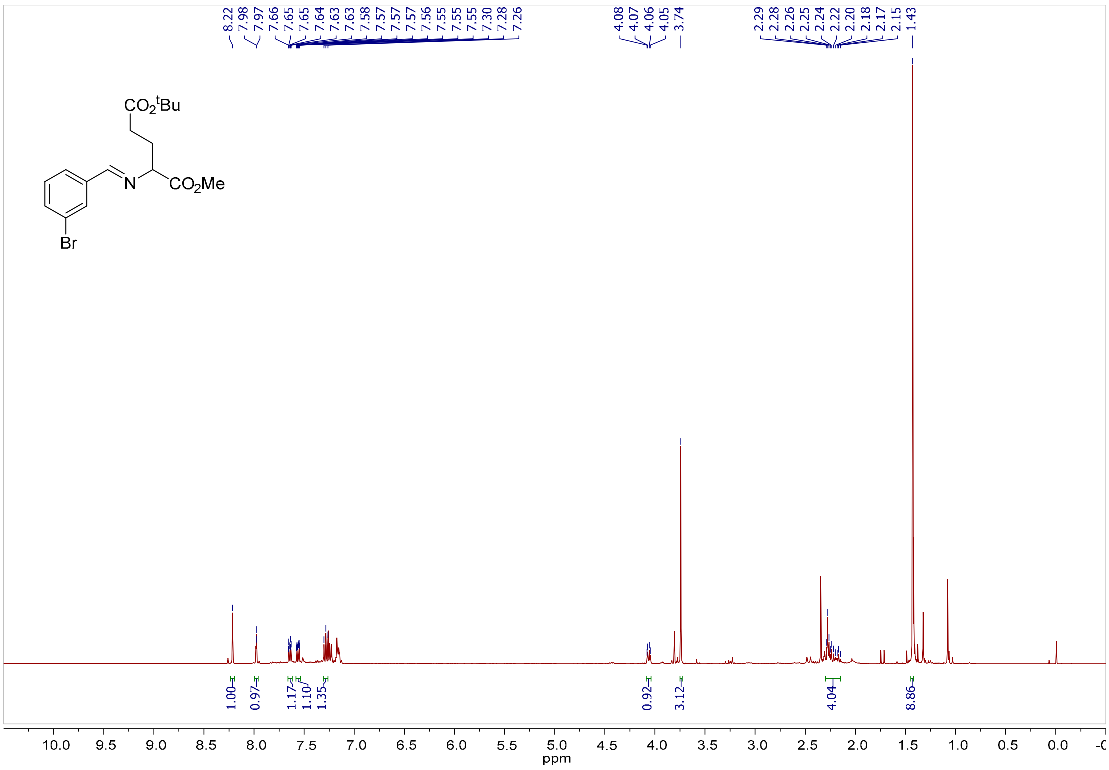

Figure S16, ^{1}H NMR of compound **2g**

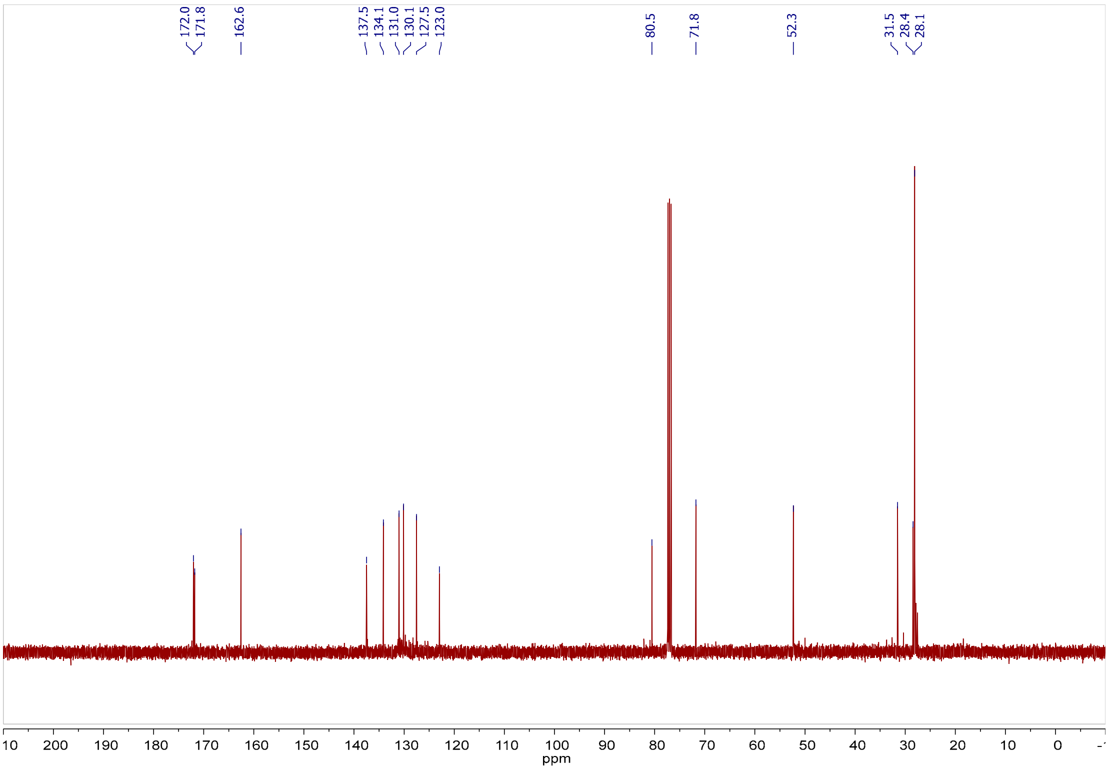

Figure S17, ^{13}C NMR of compound **2g**

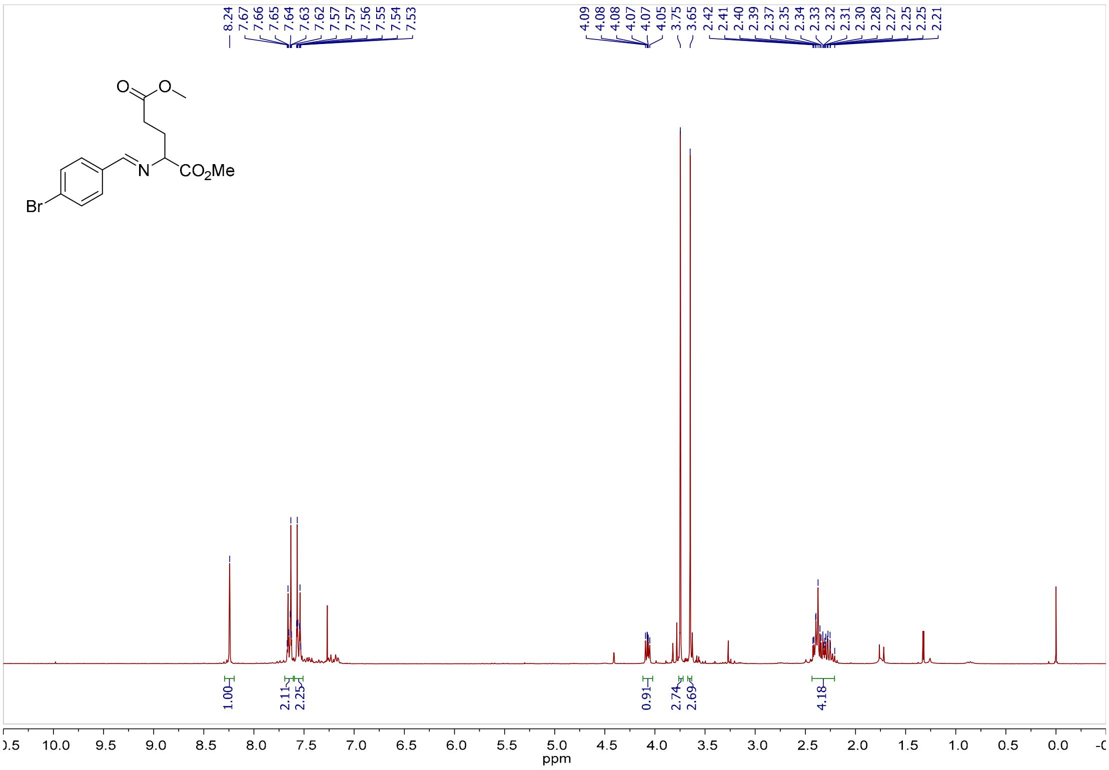

Figure S18, ^{1}H NMR of compound **2h**

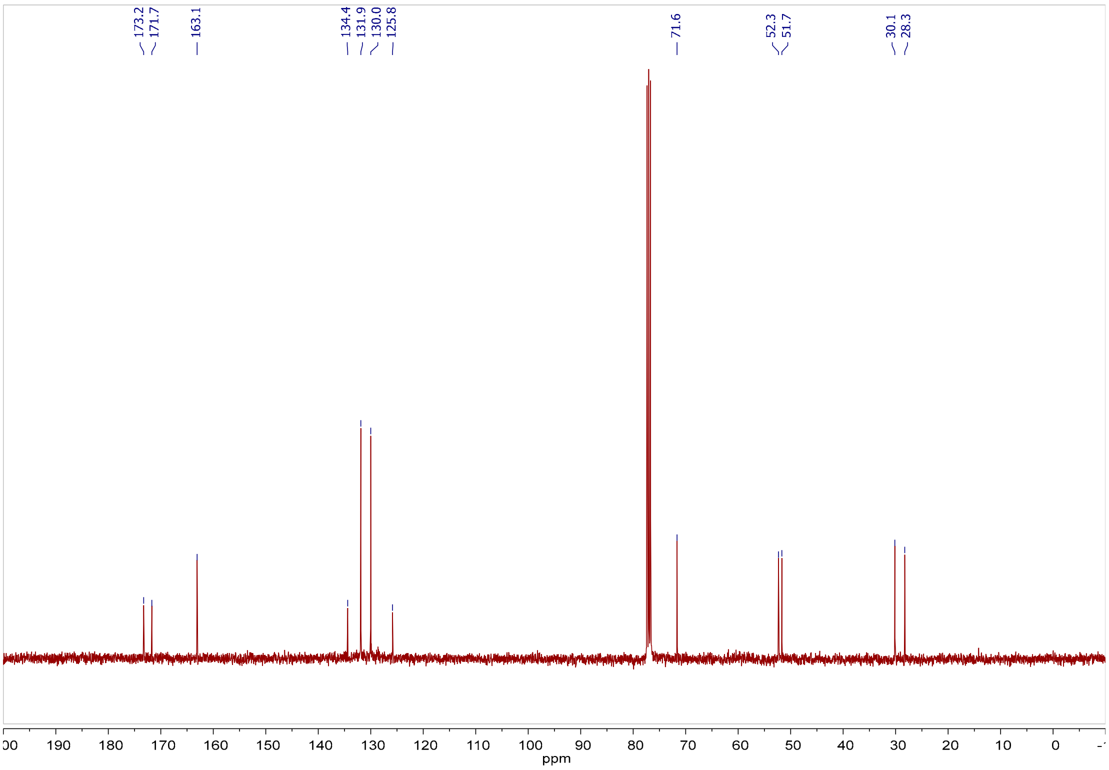

Figure S19, ^{13}C NMR of compound **2h**

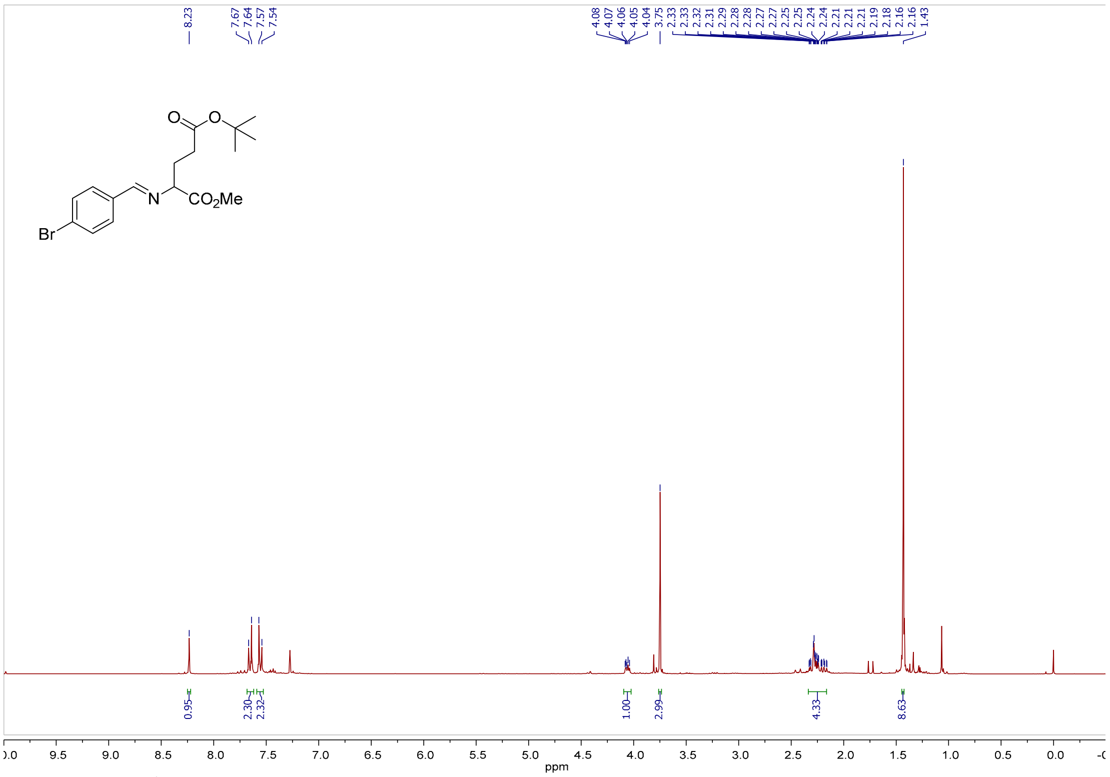

Figure S20, ^{1}H NMR of compound **2i**

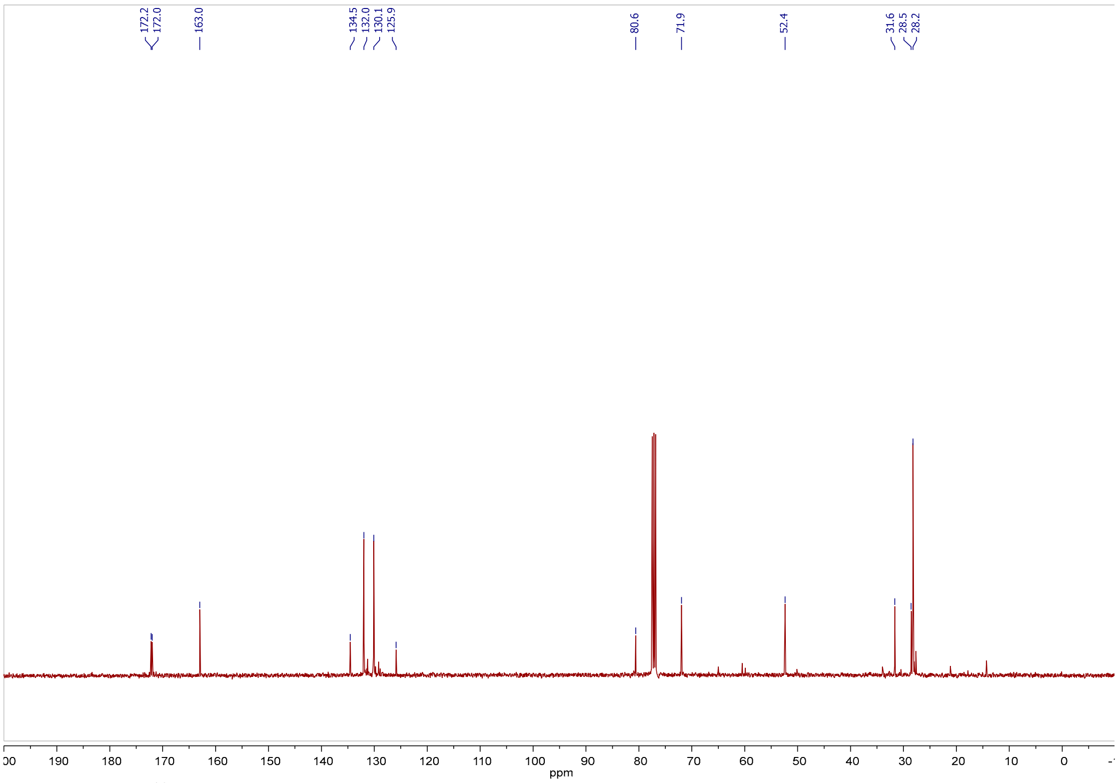

Figure S21, ^{13}C NMR of compound **2i**

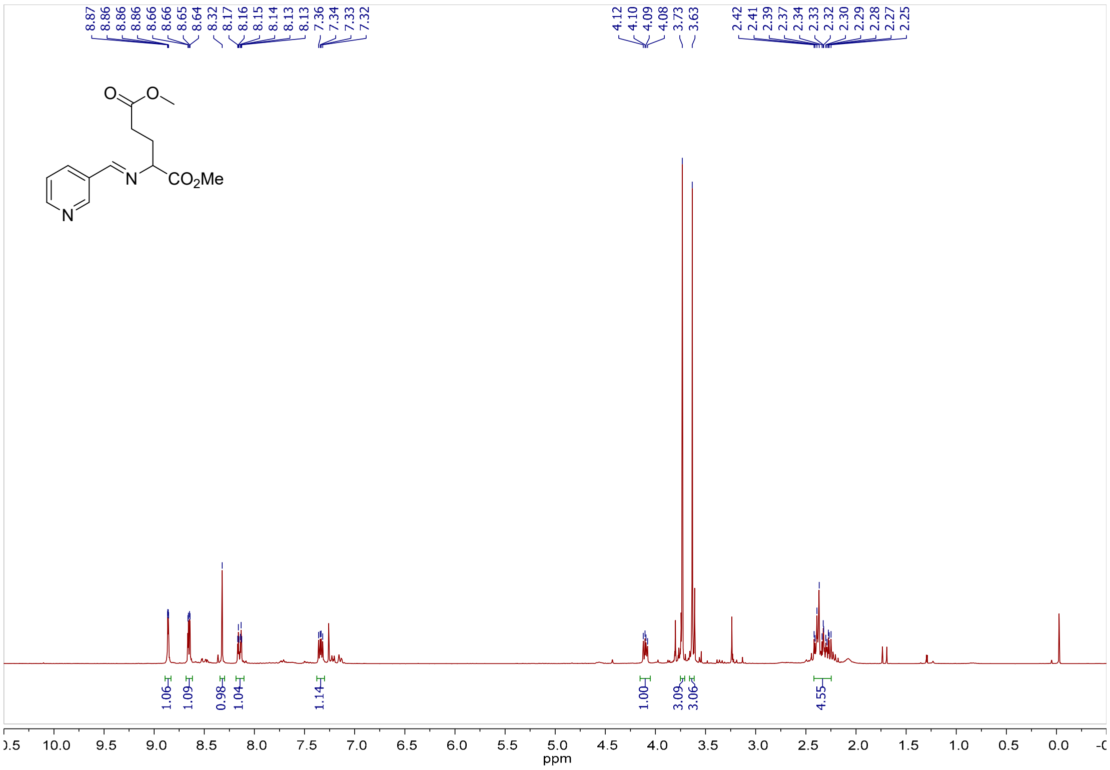

Figure S22, ^{1}H NMR of compound **2j**

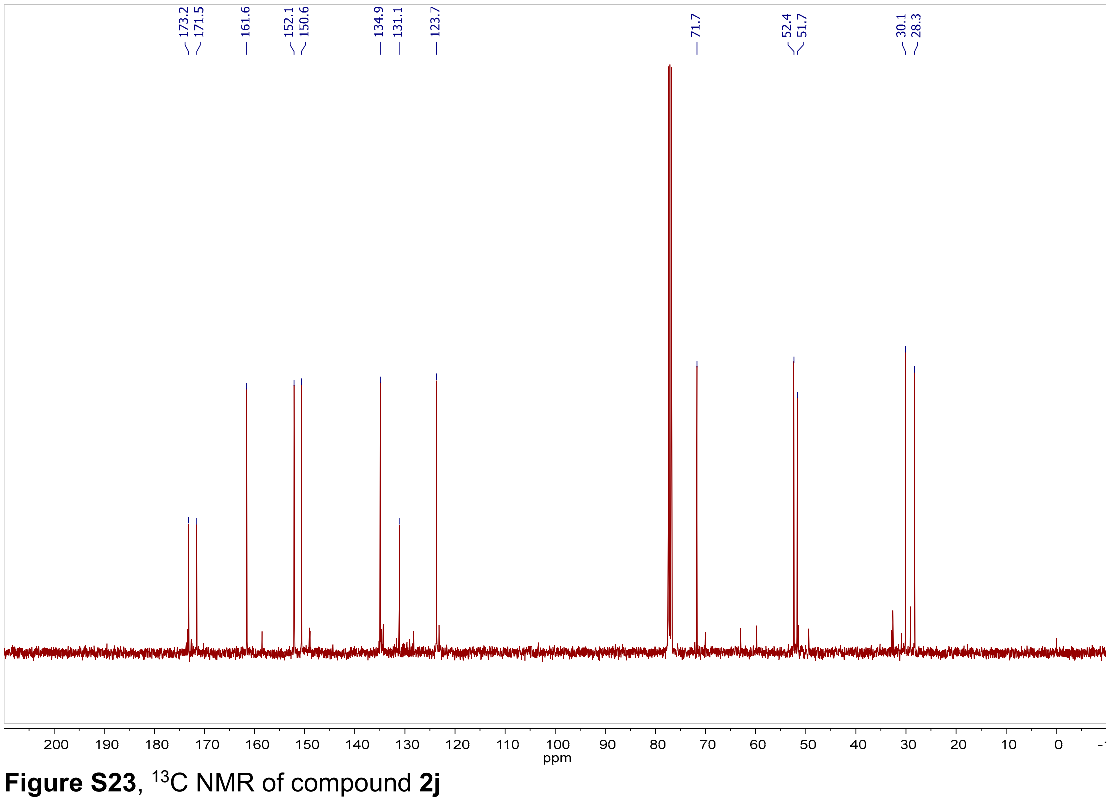

Figure S23, ^{13}C NMR of compound **2j**

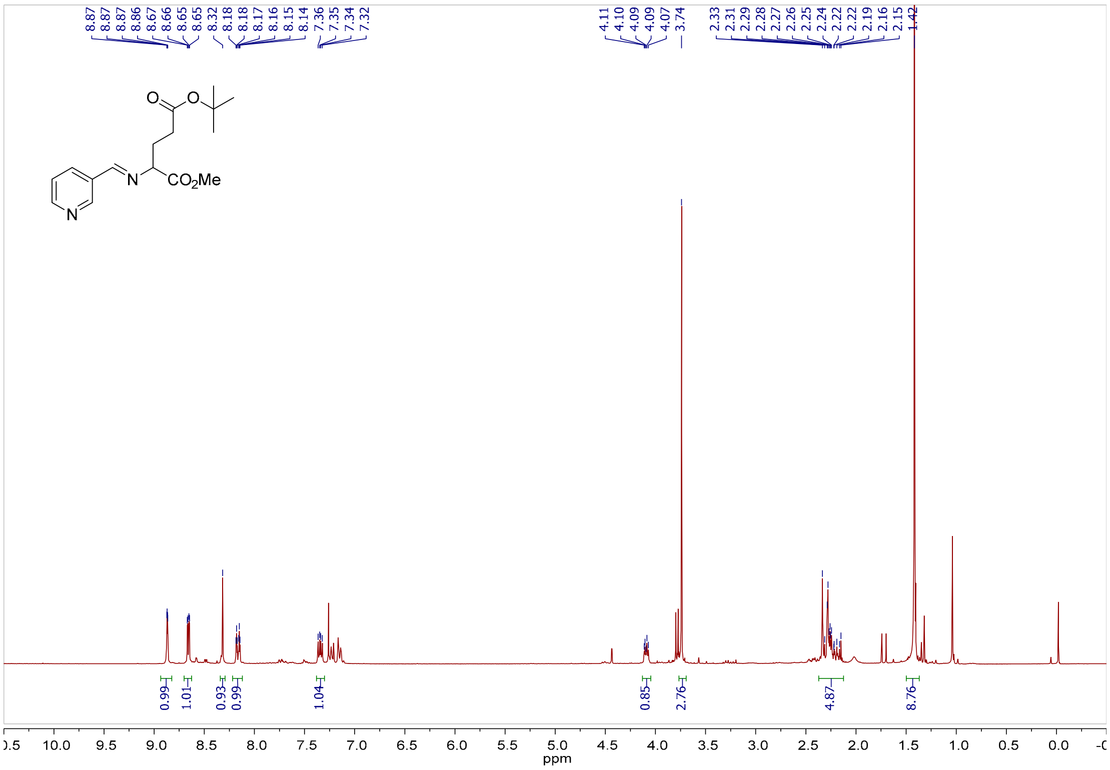

Figure S24, ^{1}H NMR of compound **2k**

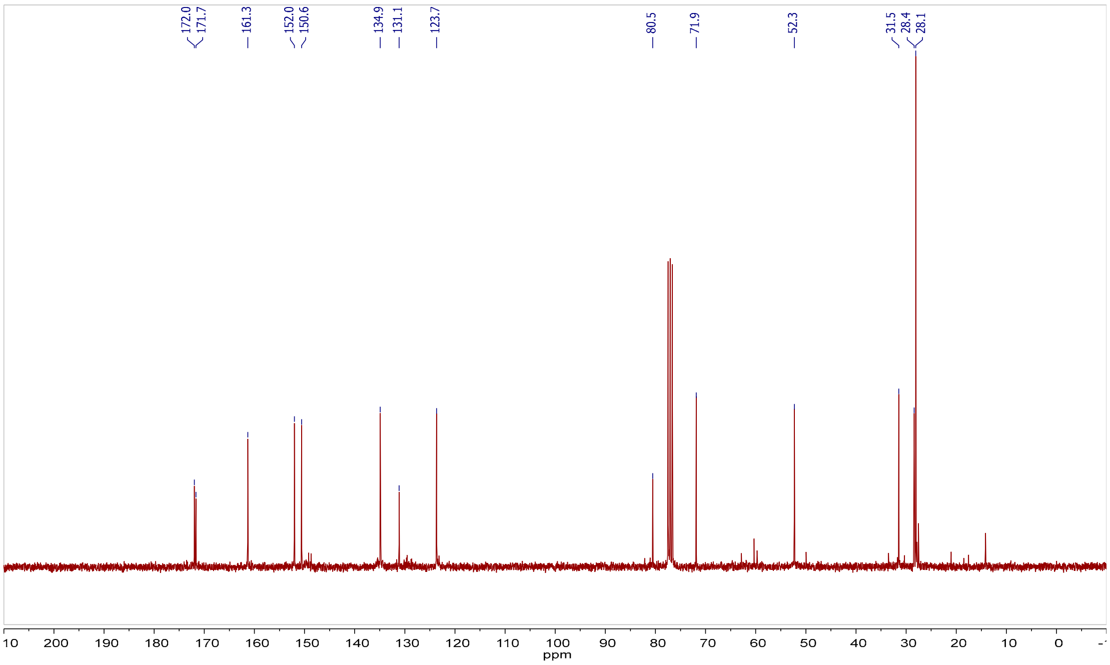

Figure S25, ^{13}C NMR of compound **2k**

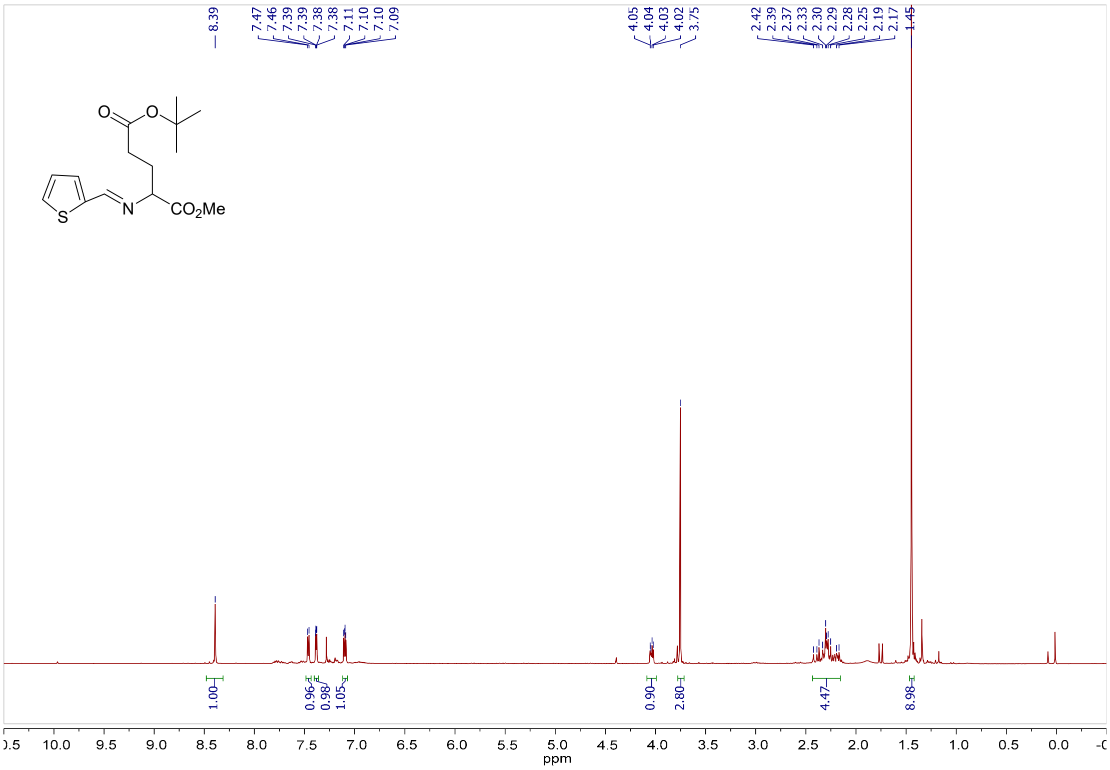

Figure S26, ^{1}H NMR of compound **2l**

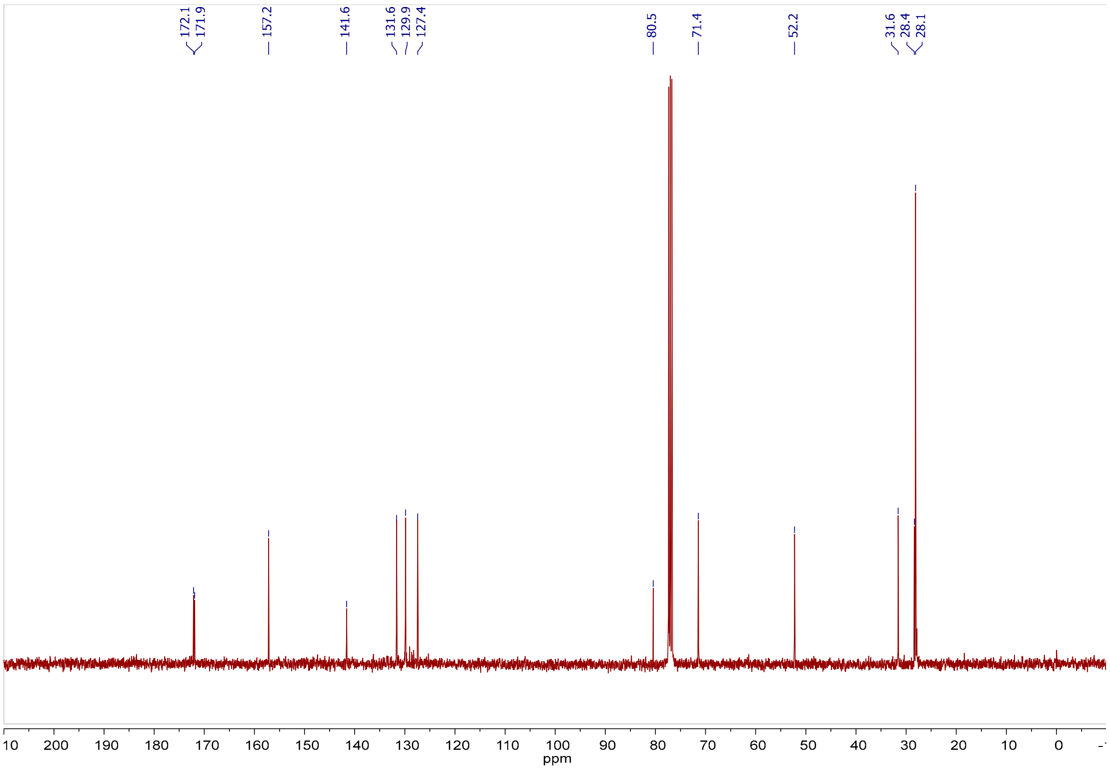

Figure S27, ^{13}C NMR of compound **2l**

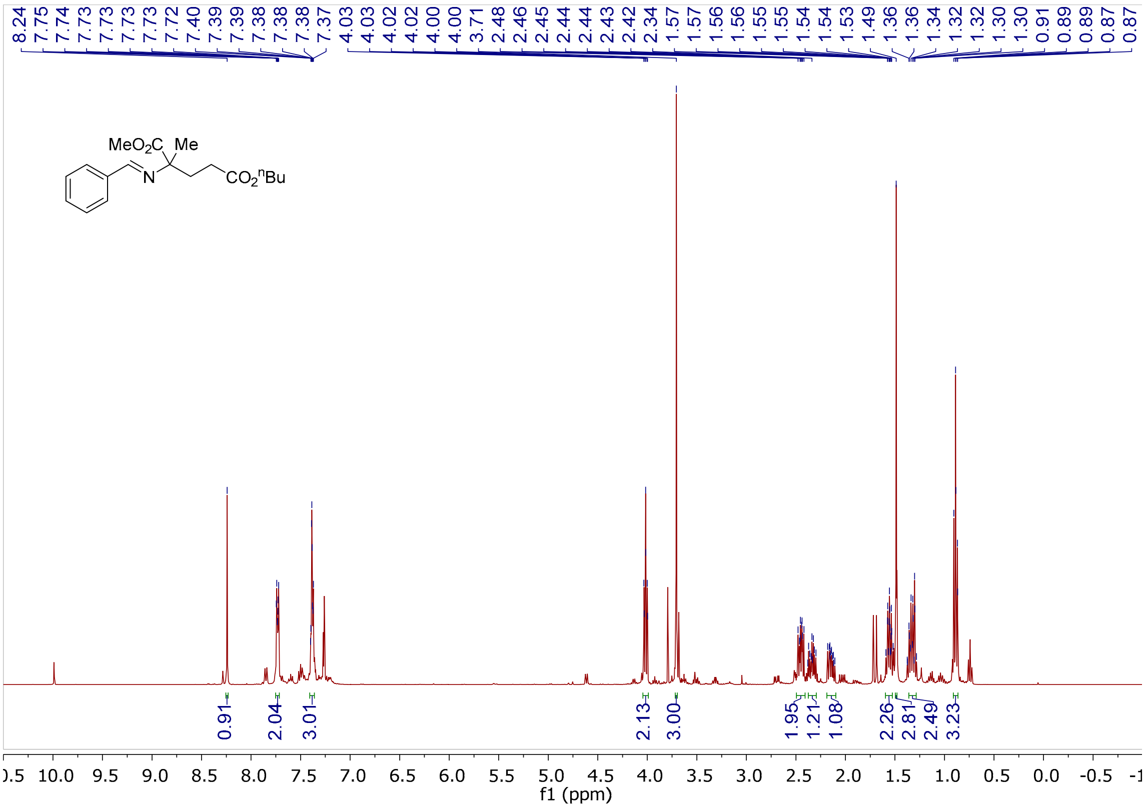

Figure S28, ^{1}H NMR of compound **2m**

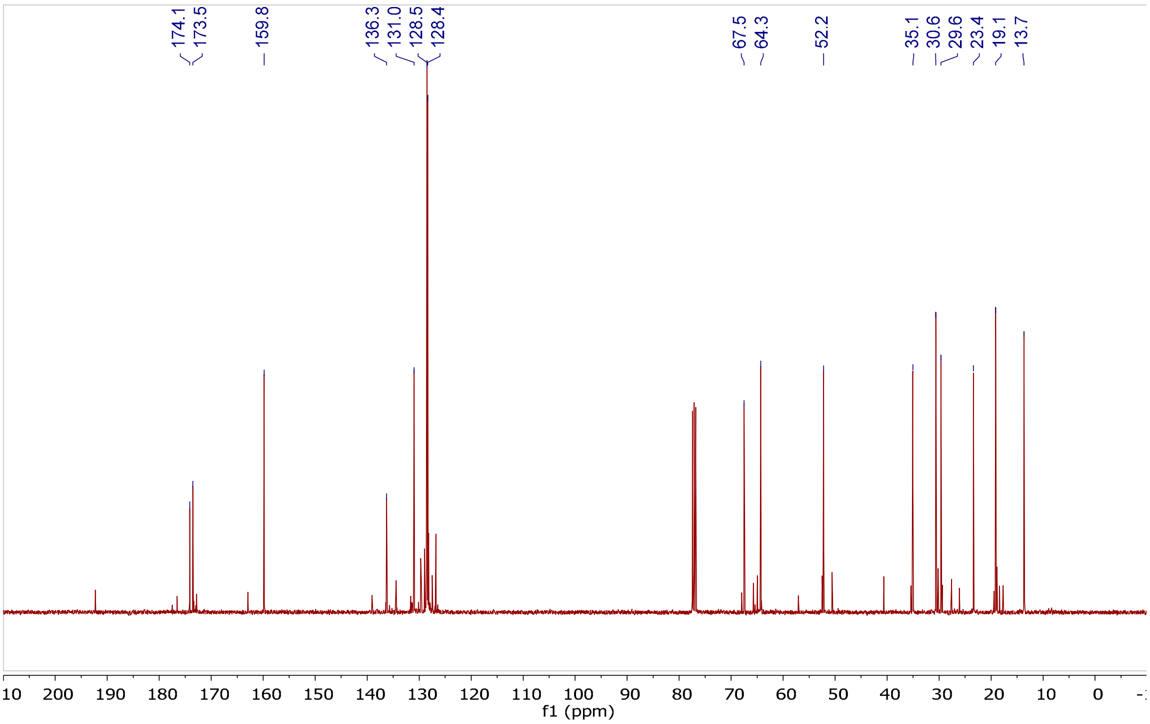

Figure S29, ^{13}C NMR of compound **2m**

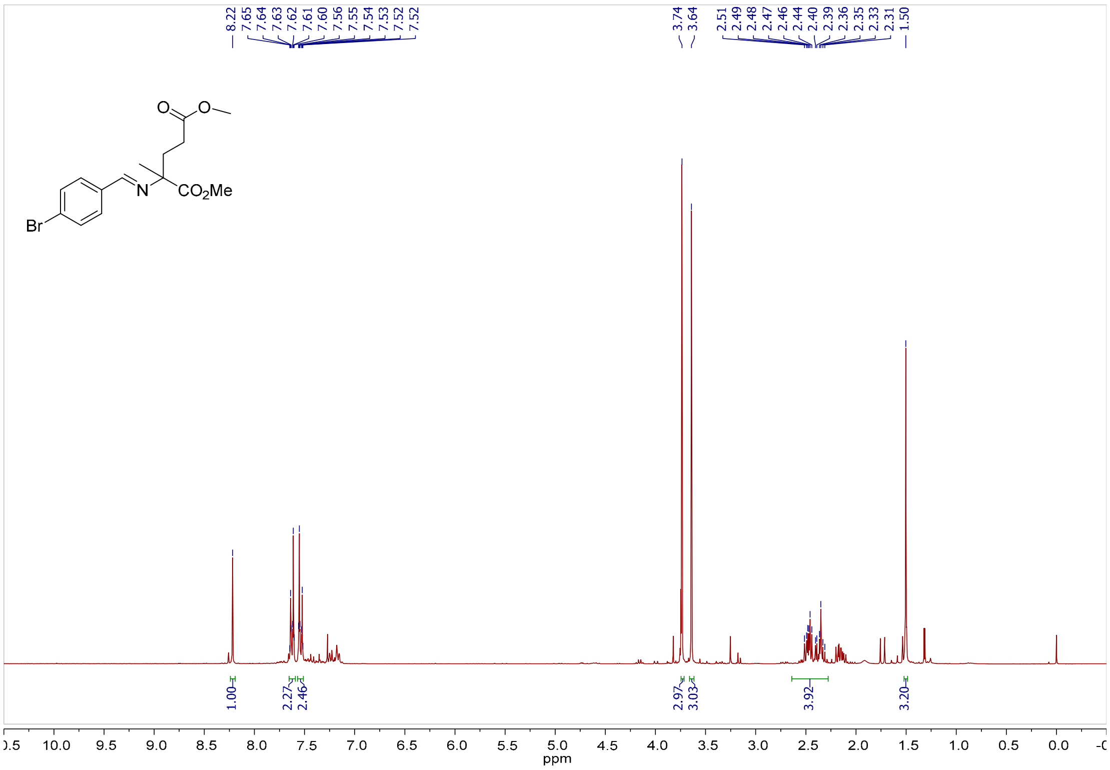

Figure S30, ^{1}H NMR of compound **2n**

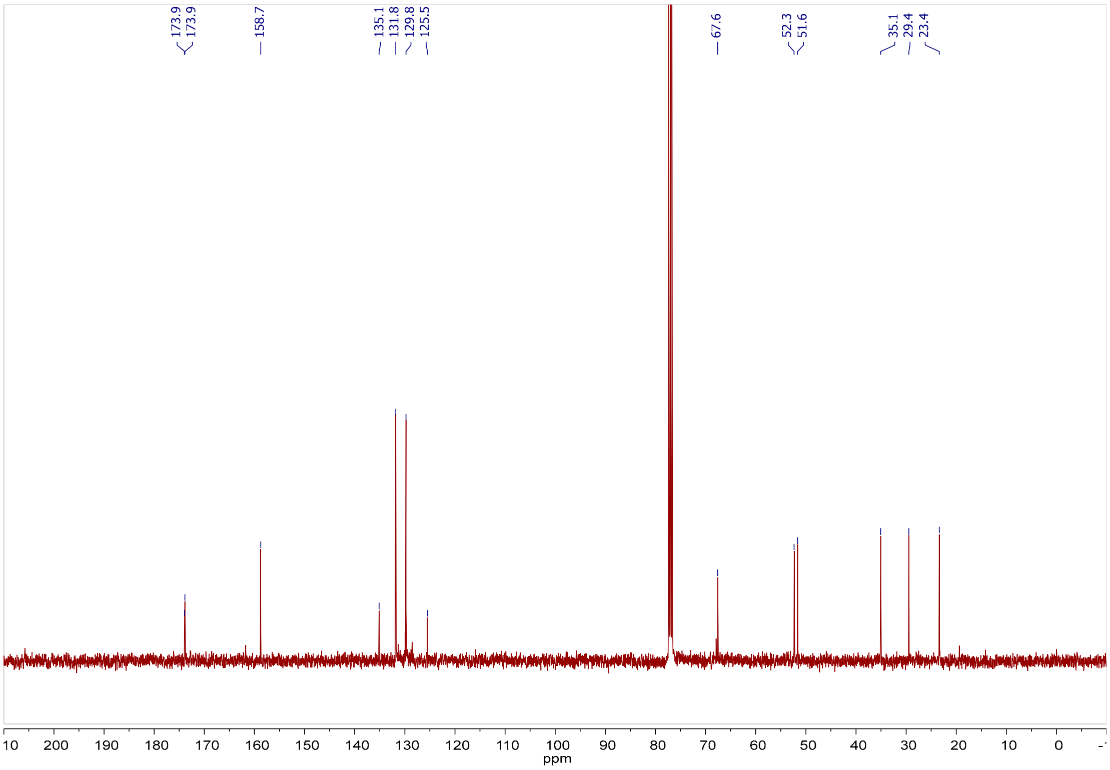

Figure S31, ^{13}C NMR of compound **2n**

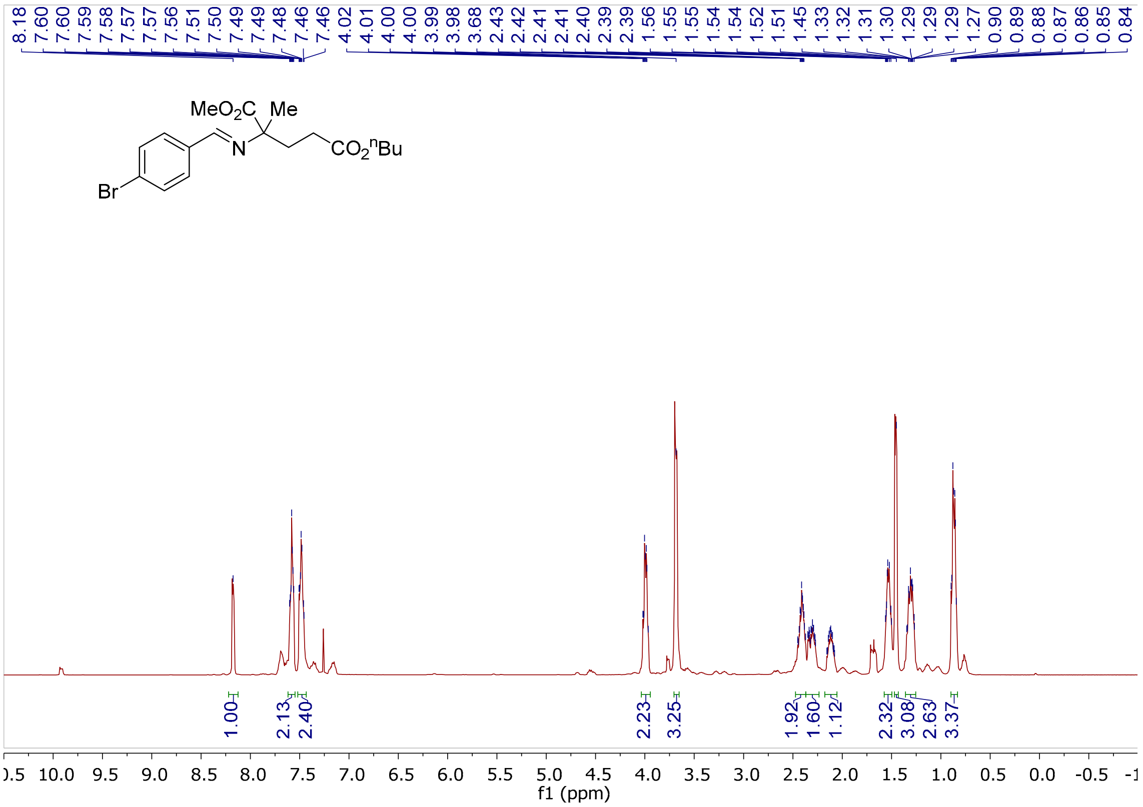

Figure S32, ^{1}H NMR of compound **2o**

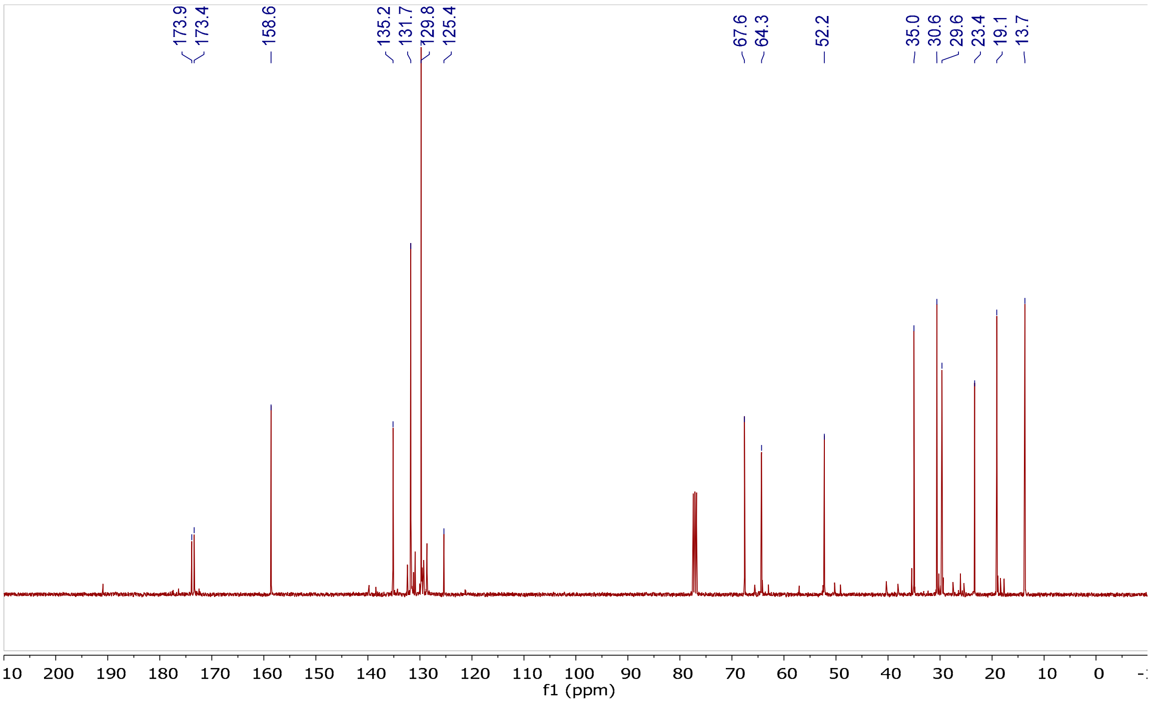

Figure S33, ^{13}C NMR of compound **2o**

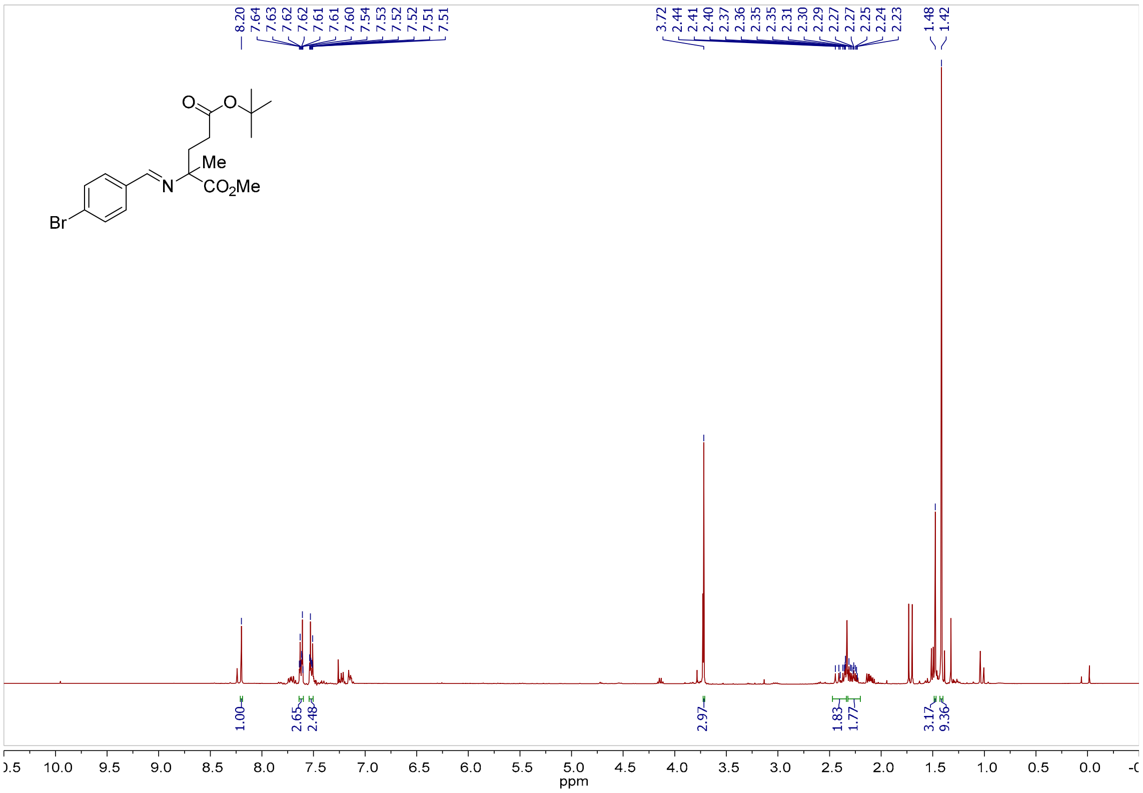

Figure S34, ^{1}H NMR of compound **2p**

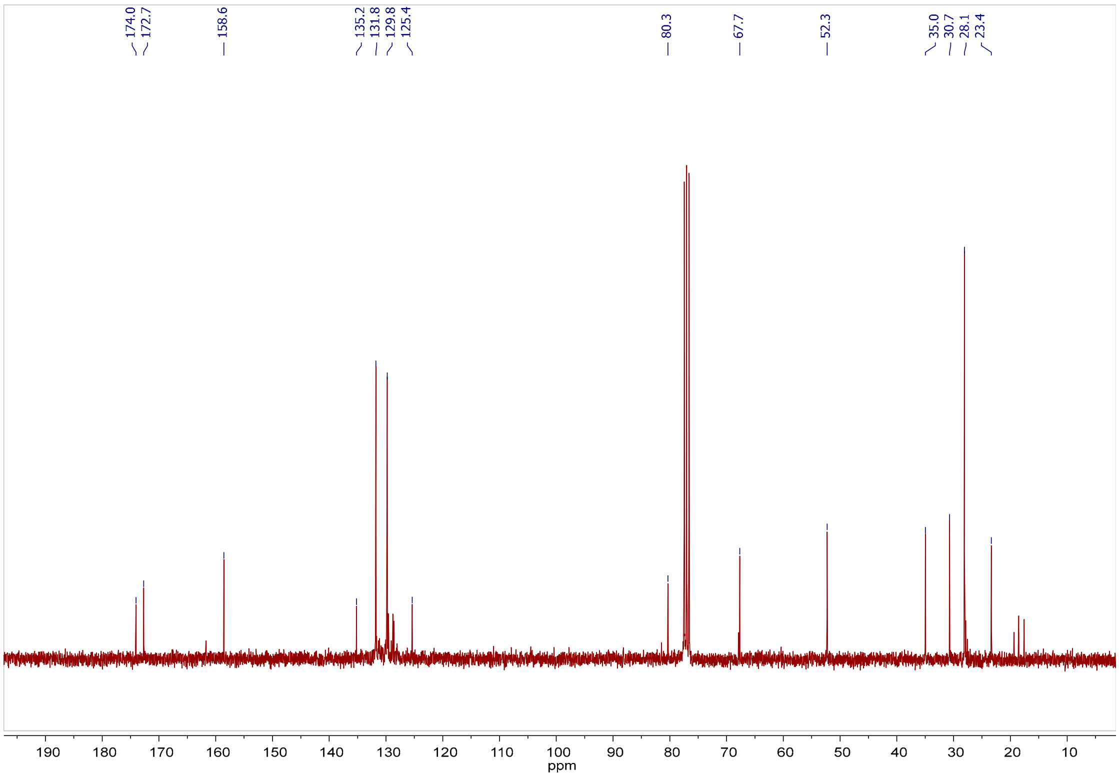

Figure S35, ^{13}C NMR of compound **2p**

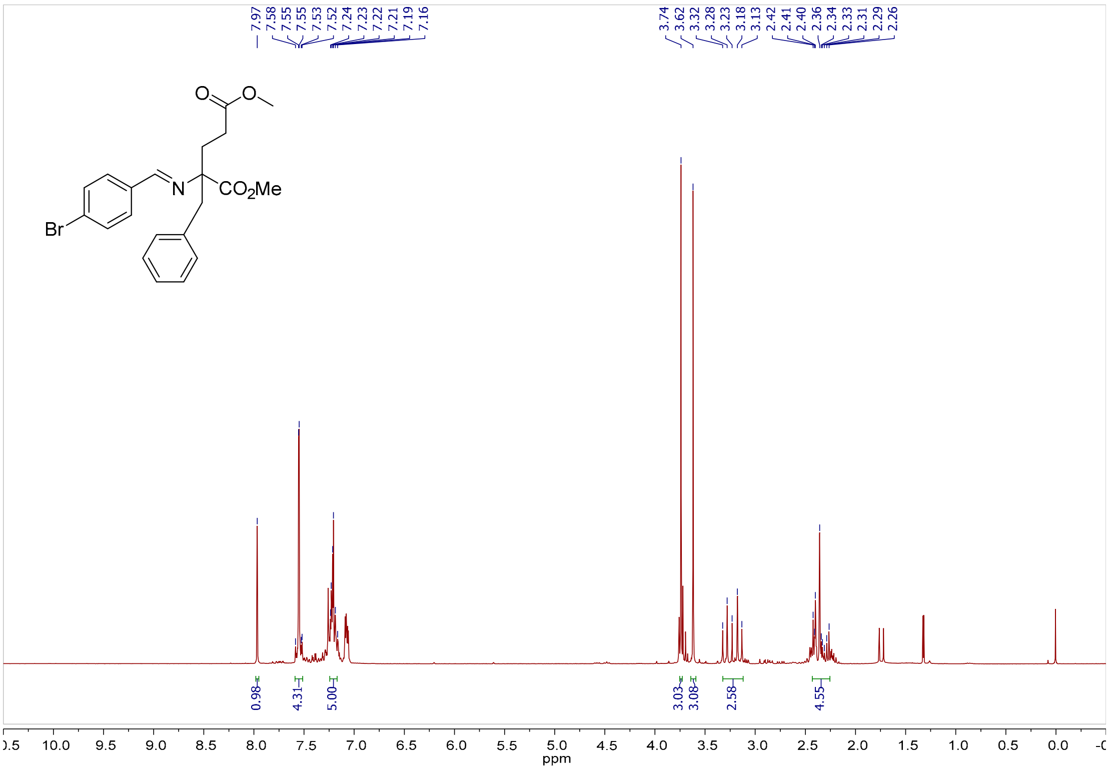

Figure S36, ^{1}H NMR of compound **2q**

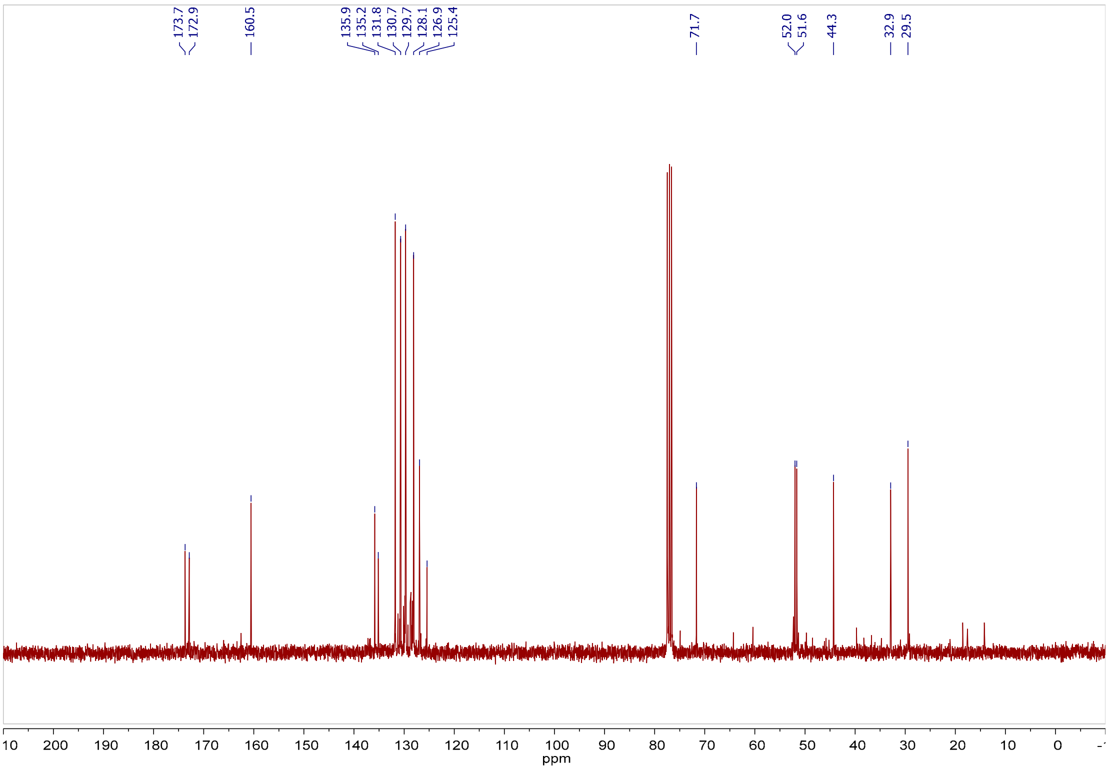

Figure S37, ^{13}C NMR of compound **2q**

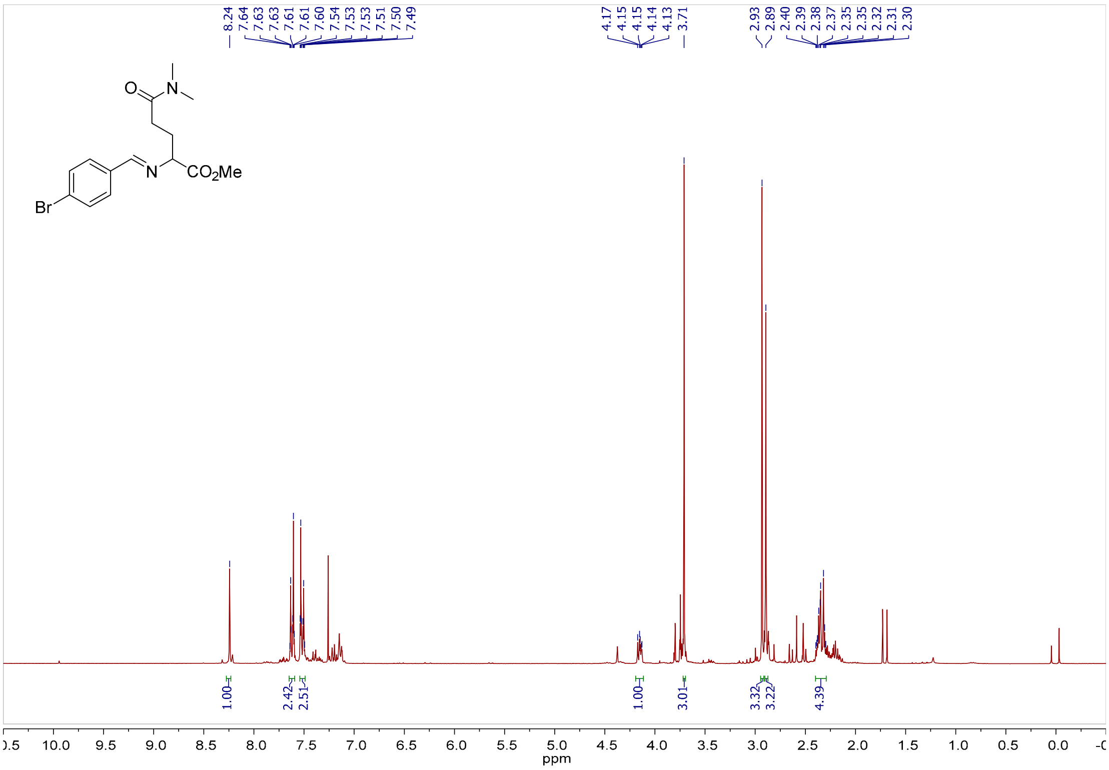

Figure S38, ^{1}H NMR of compound **2u**

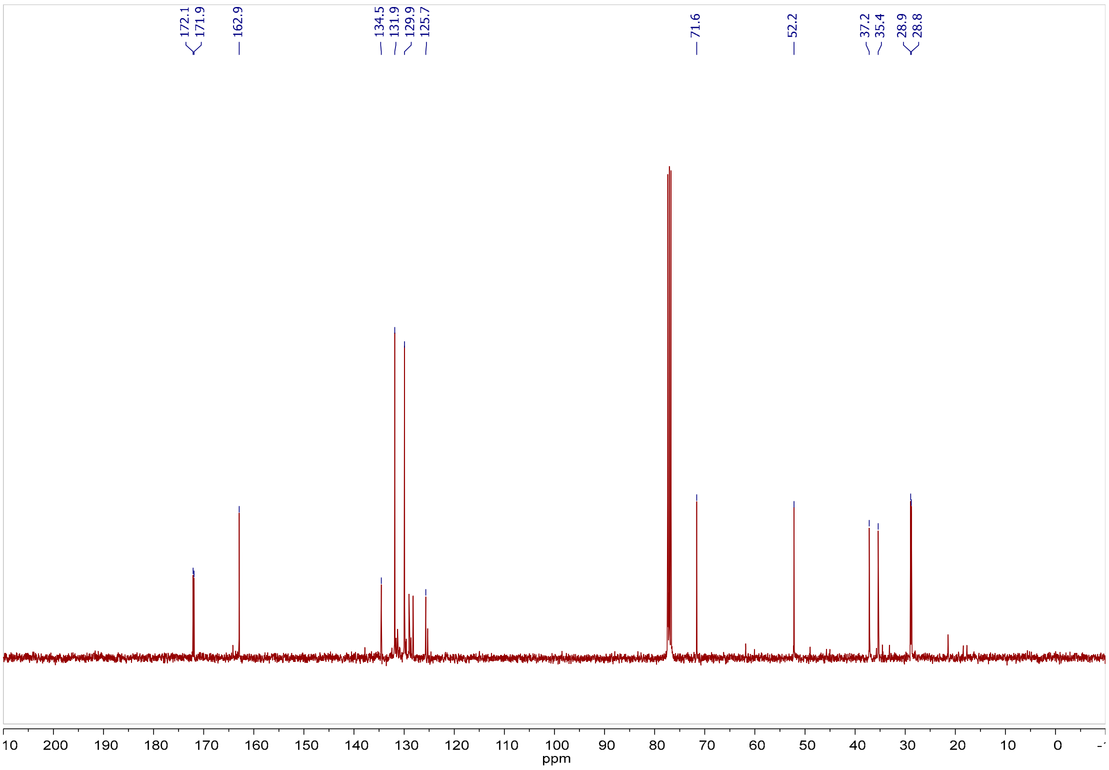

Figure S39, ^{13}C NMR of compound **2u**

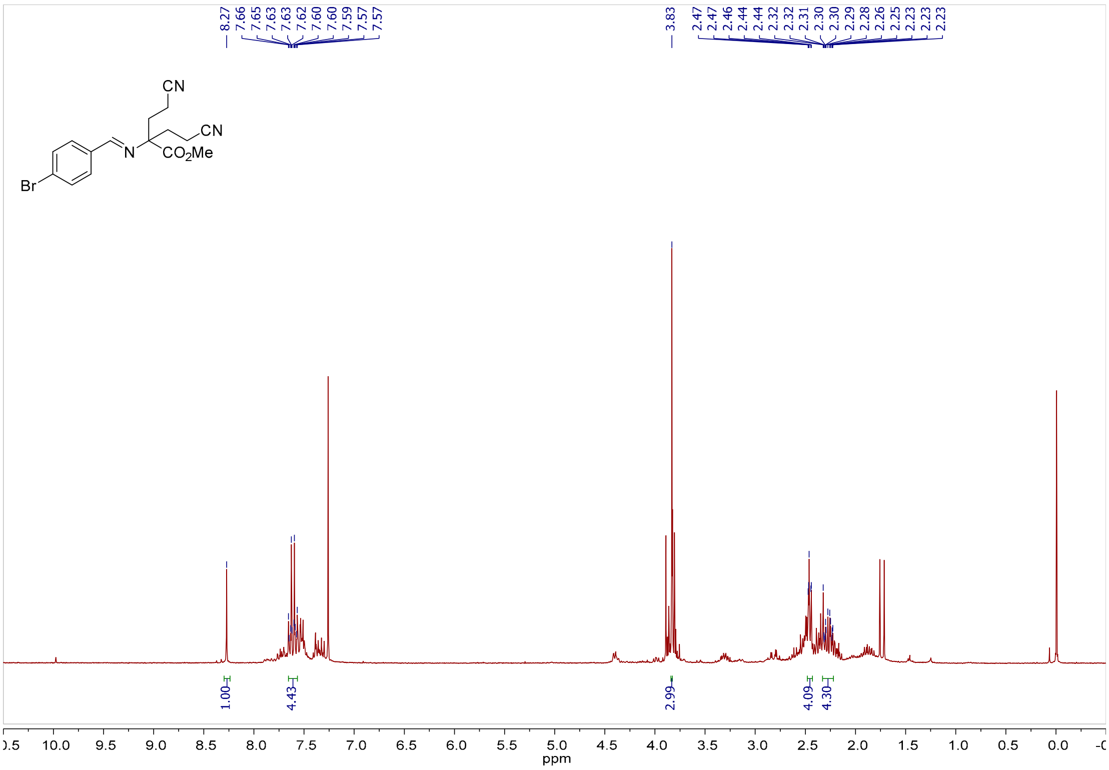

Figure S40, ^{1}H NMR of compound **6v**

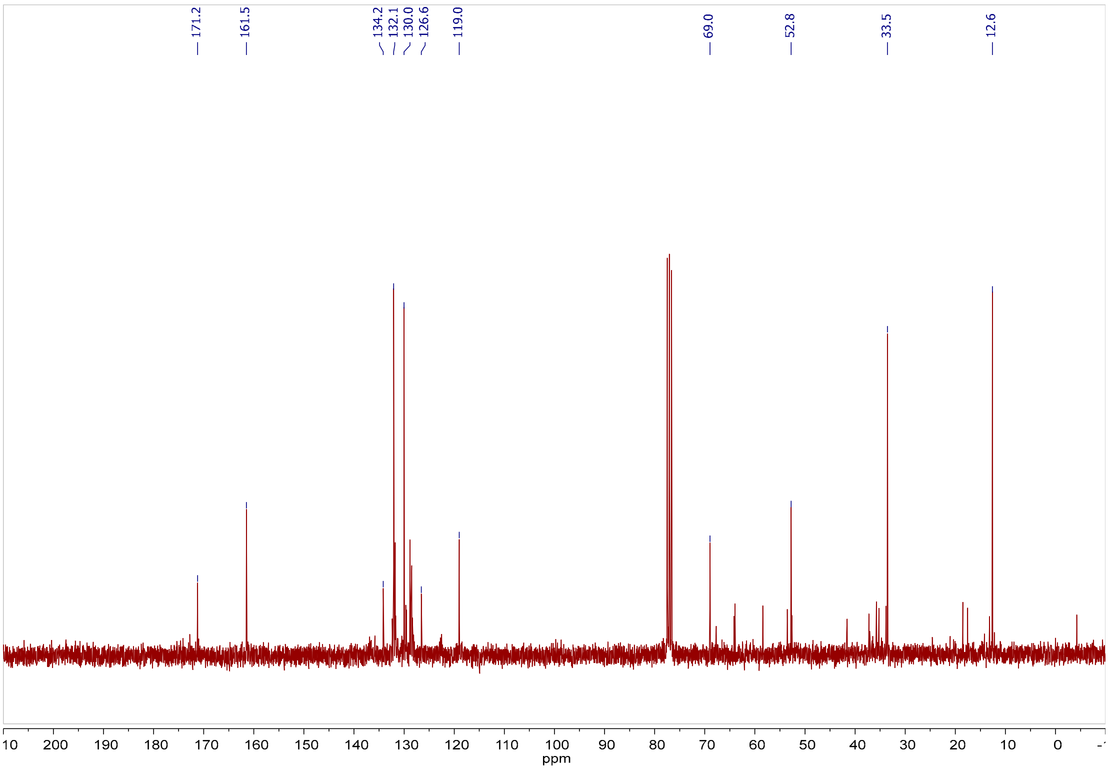

Figure S41, ^{13}C NMR of compound **6v**

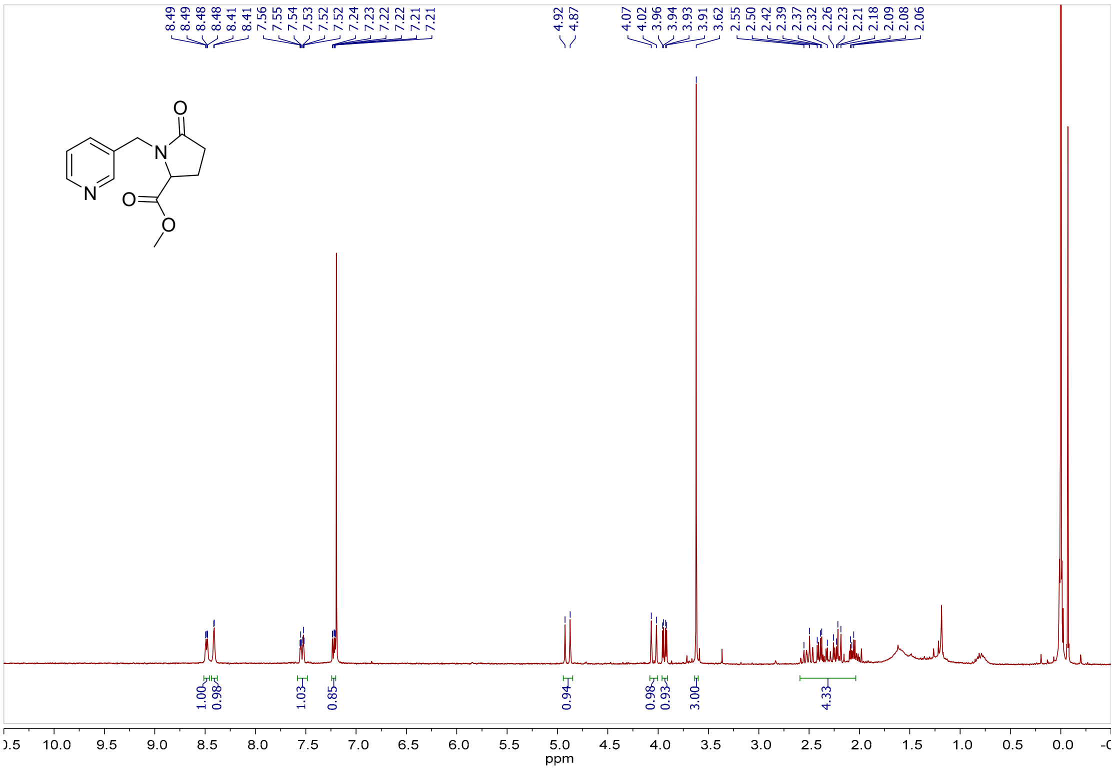

Figure S42, ^{1}H NMR of compound **8a**

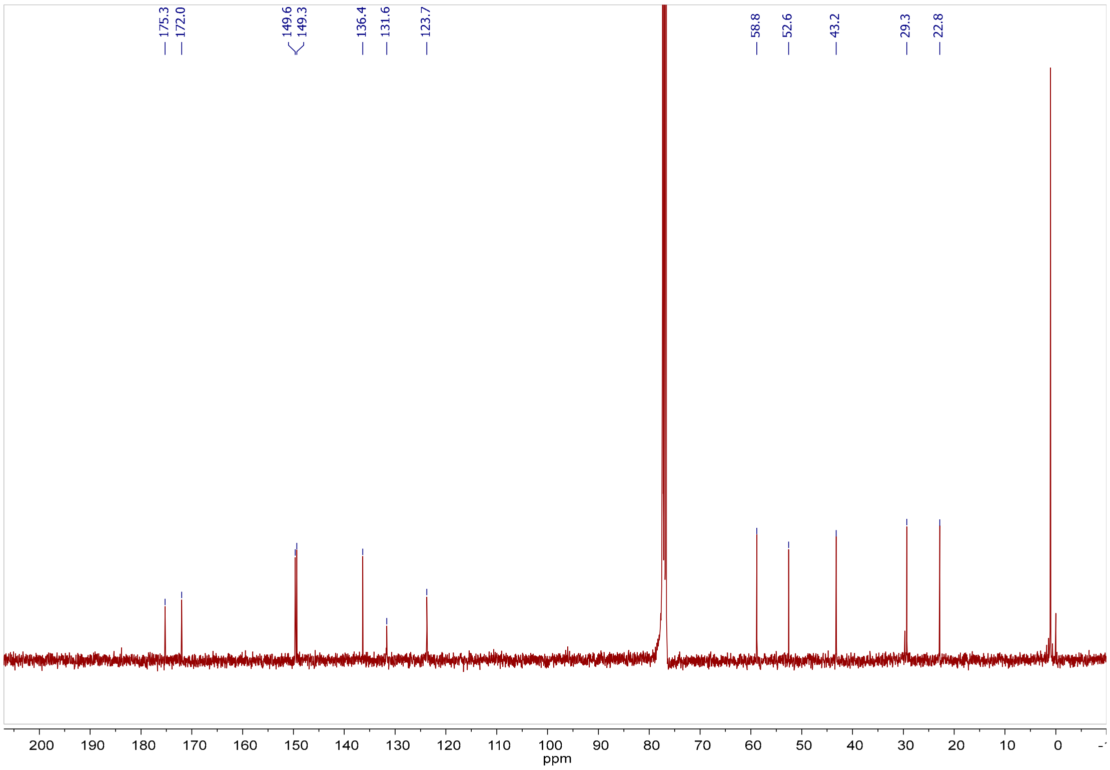

Figure S43, ^{13}C NMR of compound **8a**

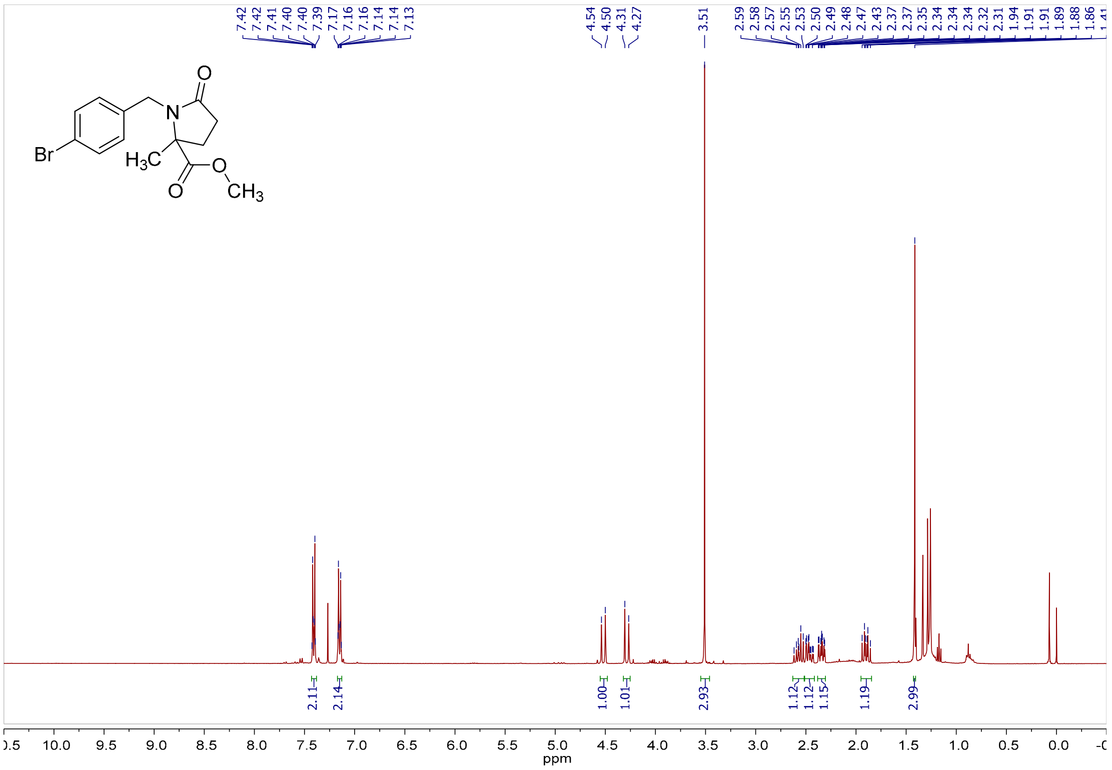

Figure S44, ^{1}H NMR of compound **8b**

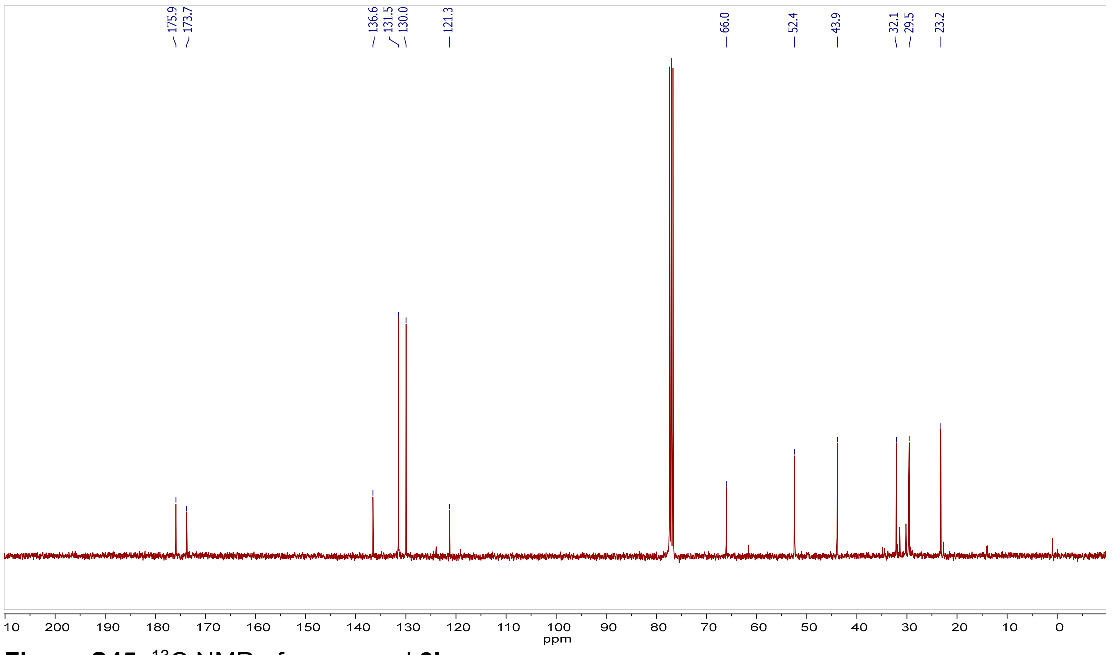

Figure S45, ^{13}C NMR of compound **8b**

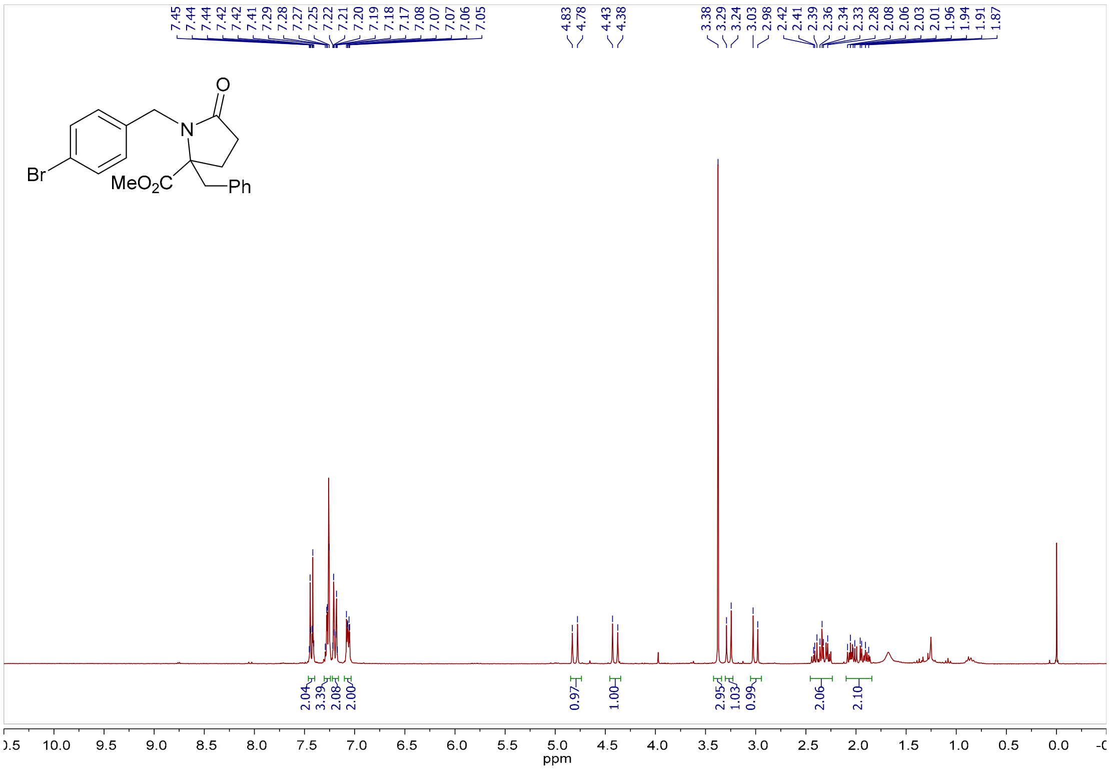

Figure S46, ^{1}H NMR of compound **8c**

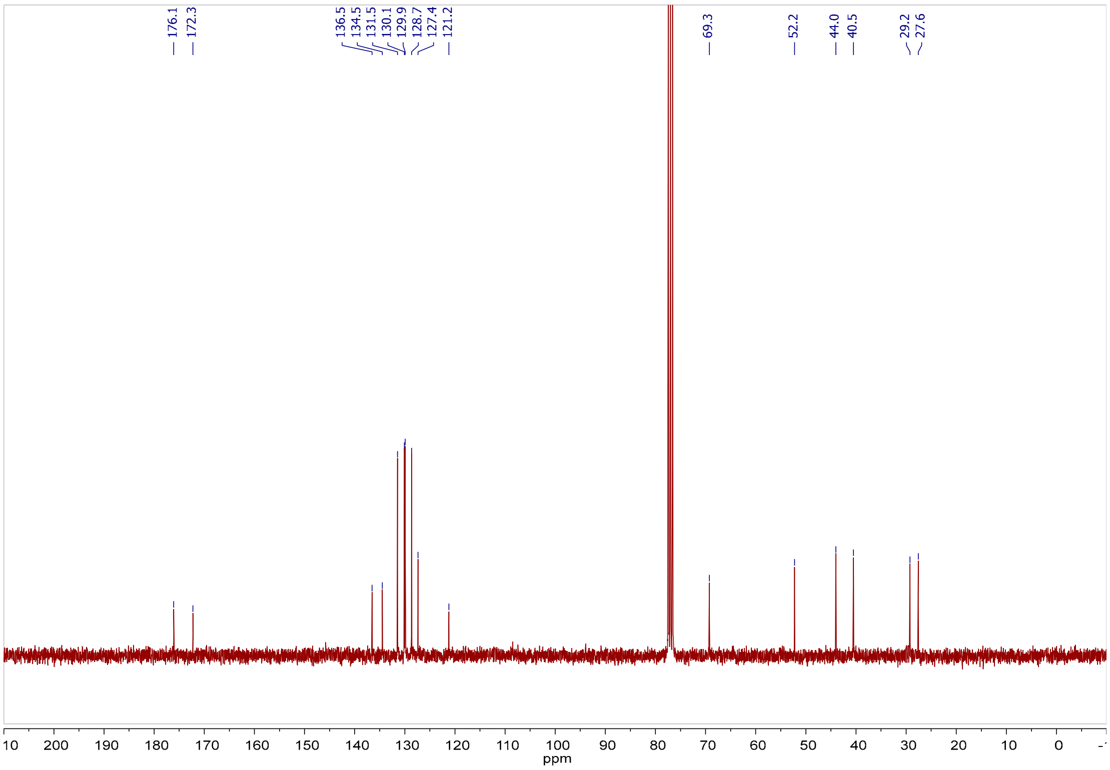

Figure S47, ^{13}C NMR of compound **8c**

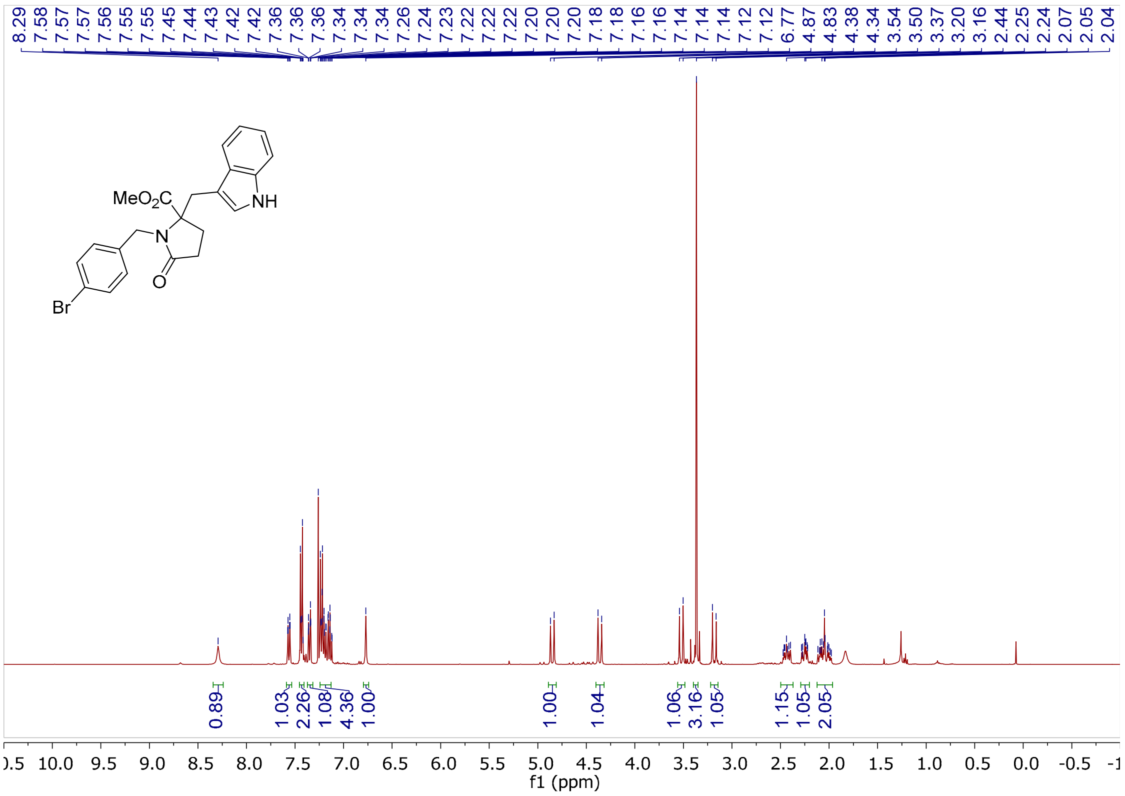

Figure S48, ^{1}H NMR of compound **8d**

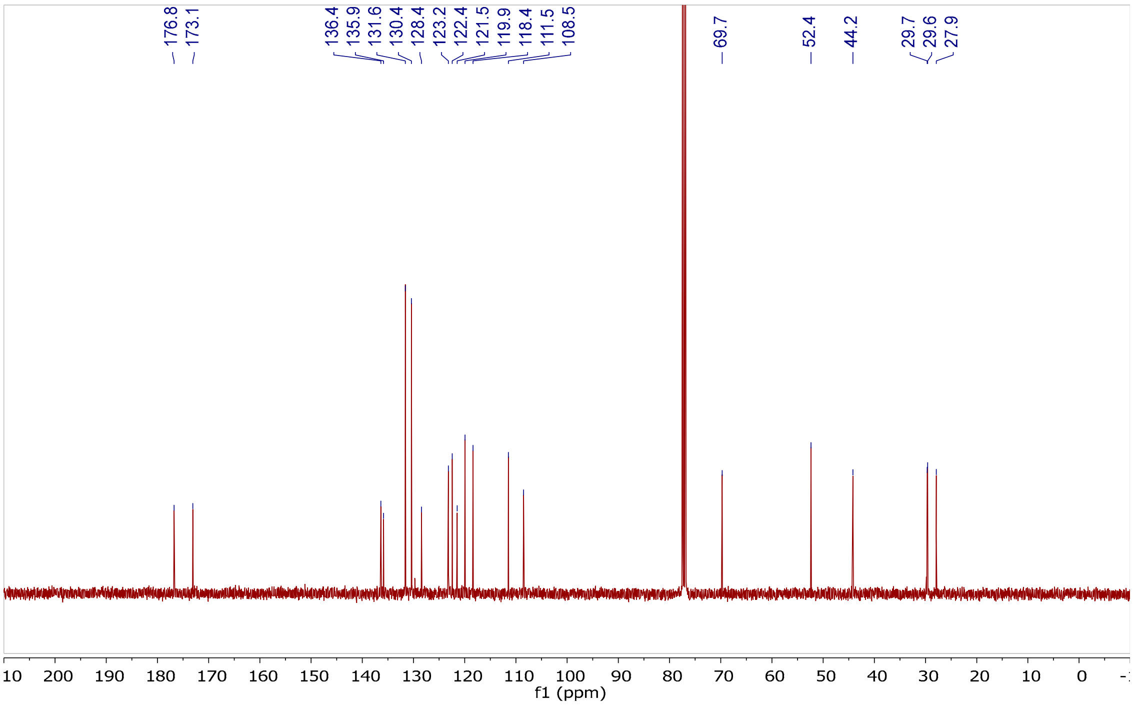

Figure S49, ^{13}C NMR of compound **8d**

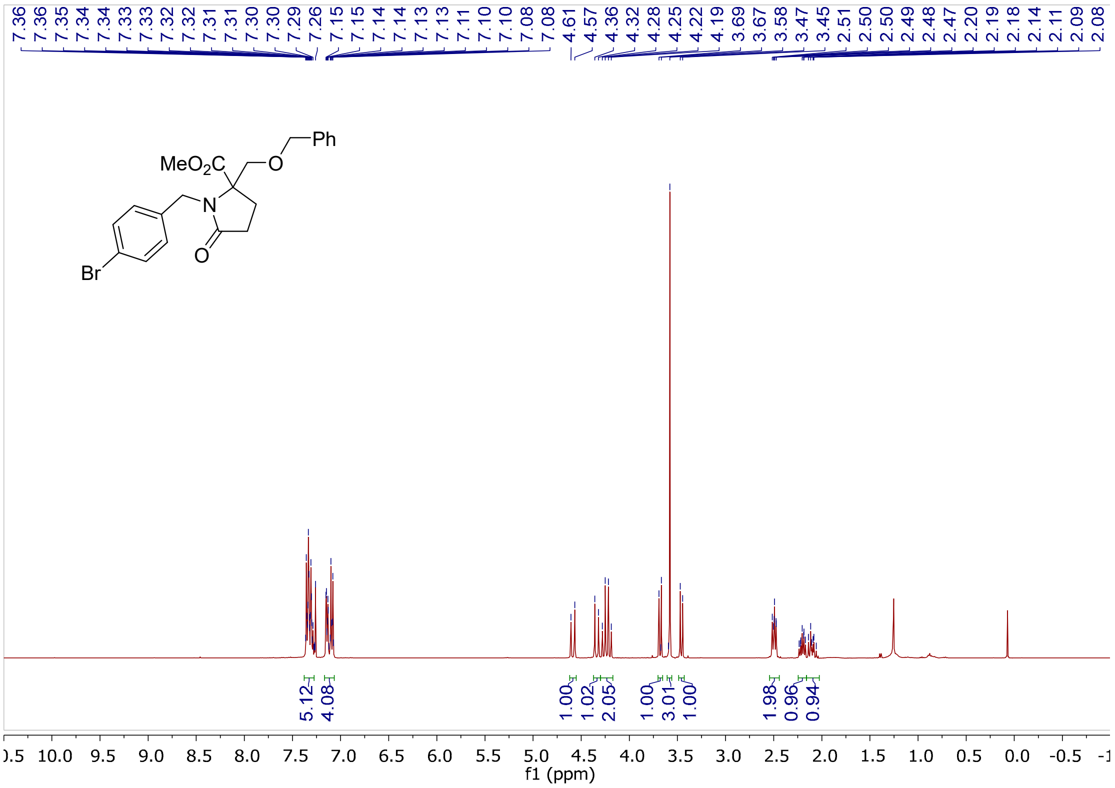

Figure S50, ^{1}H NMR of compound **8e**

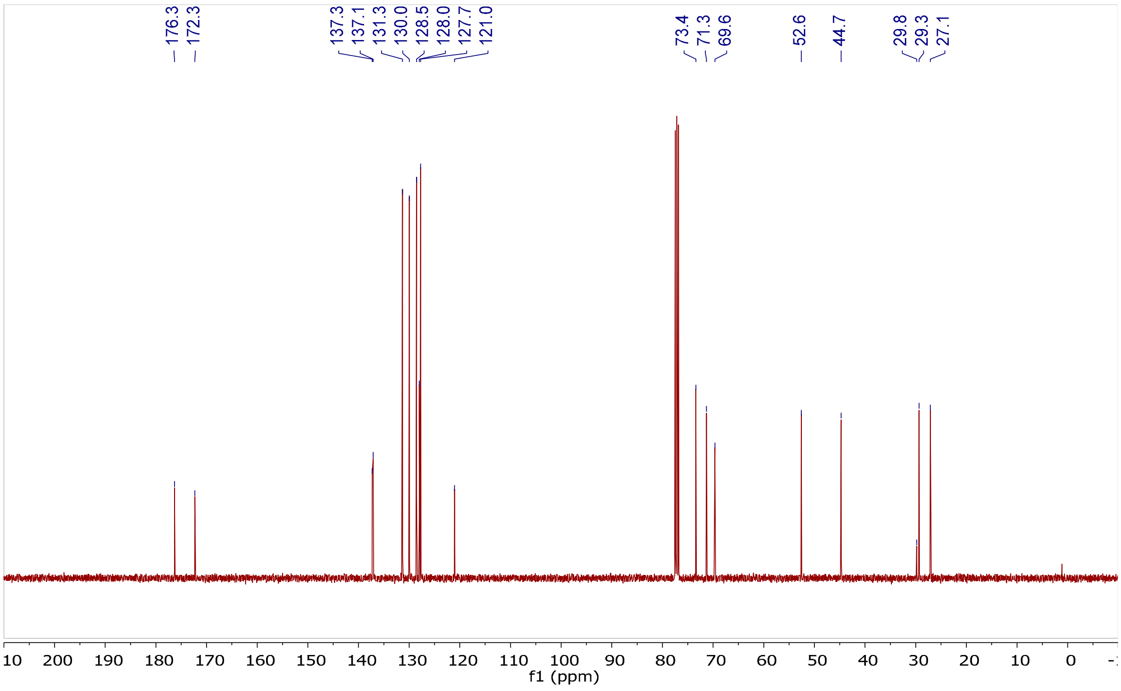

Figure S51, ^{13}C NMR of compound **8e**

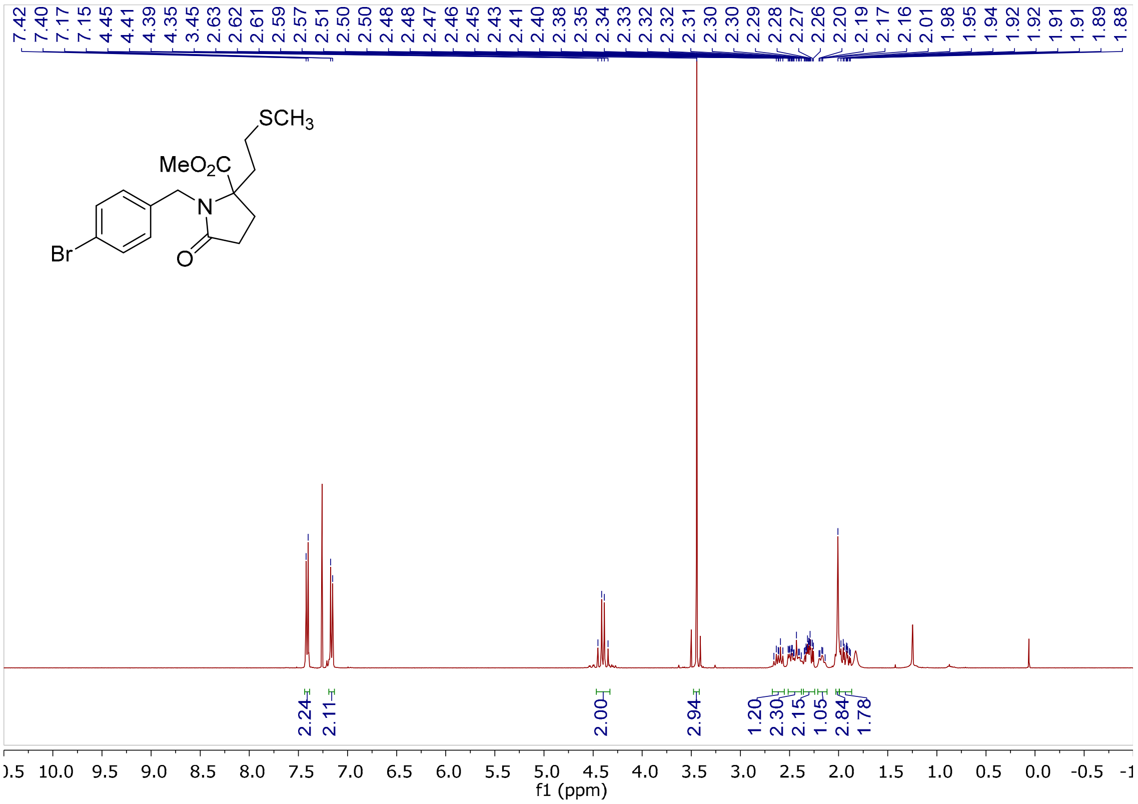

Figure S52, ^{1}H NMR of compound **8f**

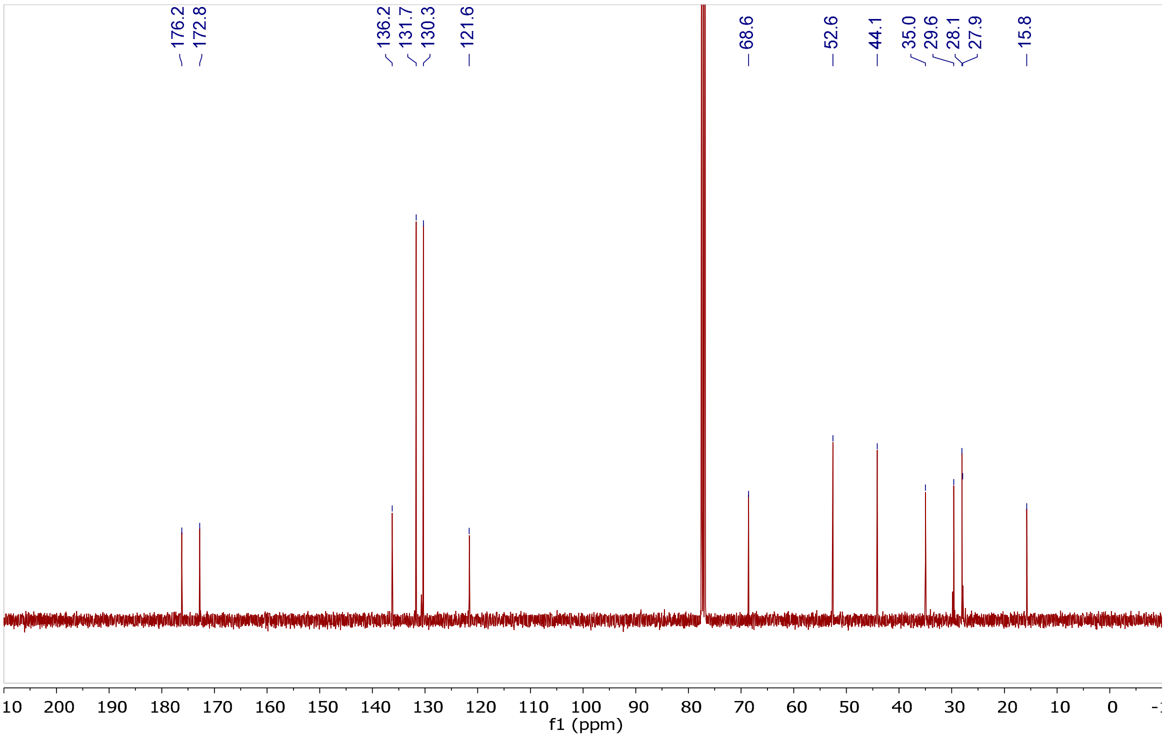

Figure S53, ^{13}C NMR of compound **8f**

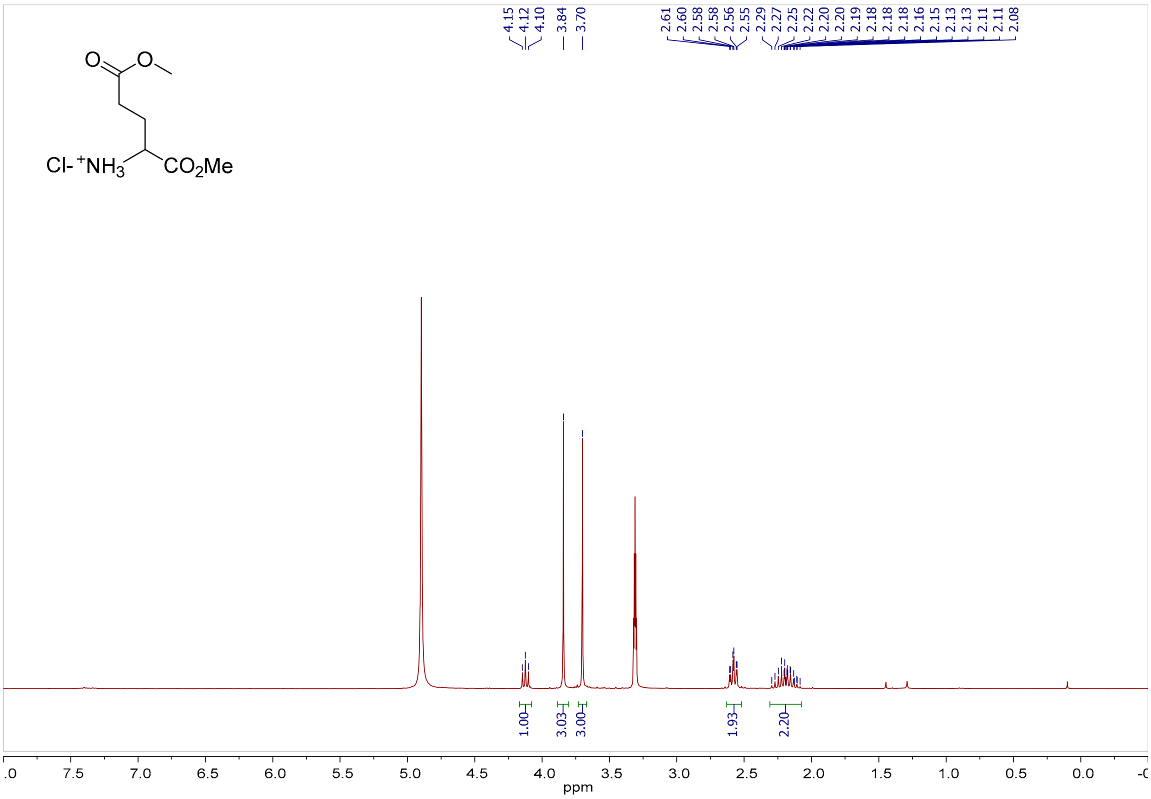

Figure S54, ^{1}H NMR of compound **9**

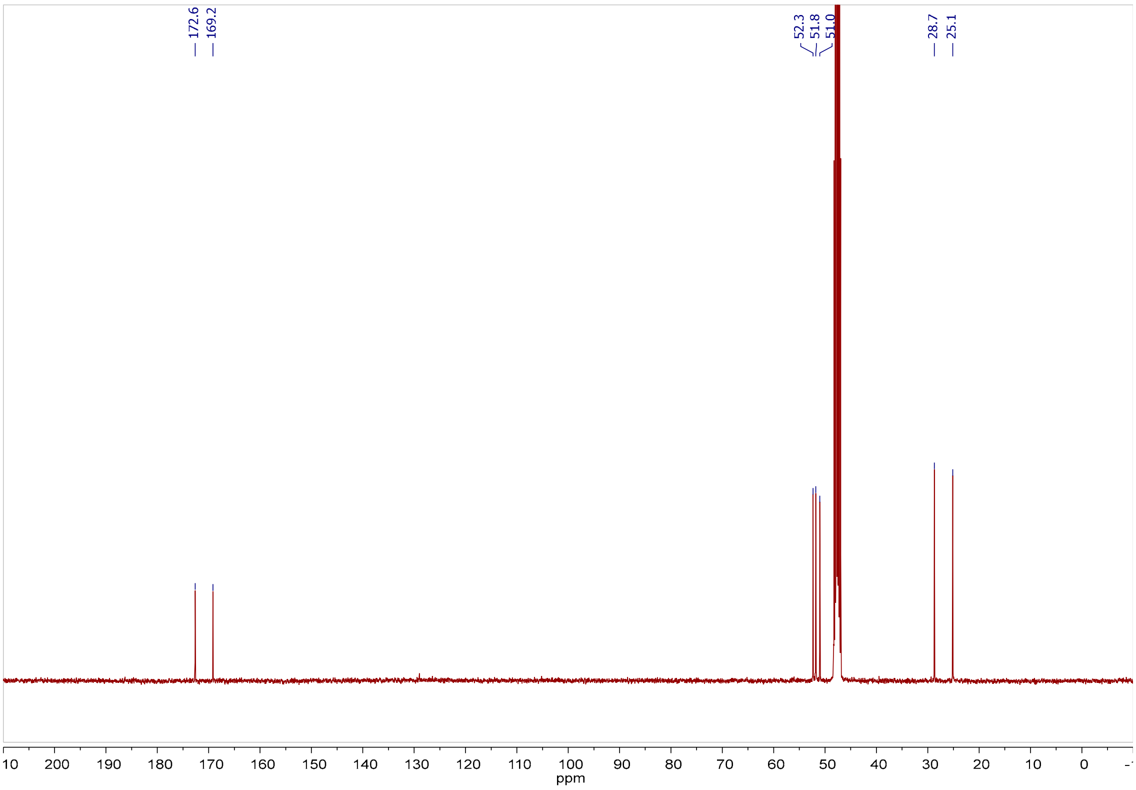

Figure S55, ^{13}C NMR of compound **9**

2. REPRESENTATIVE FTIR COPIES

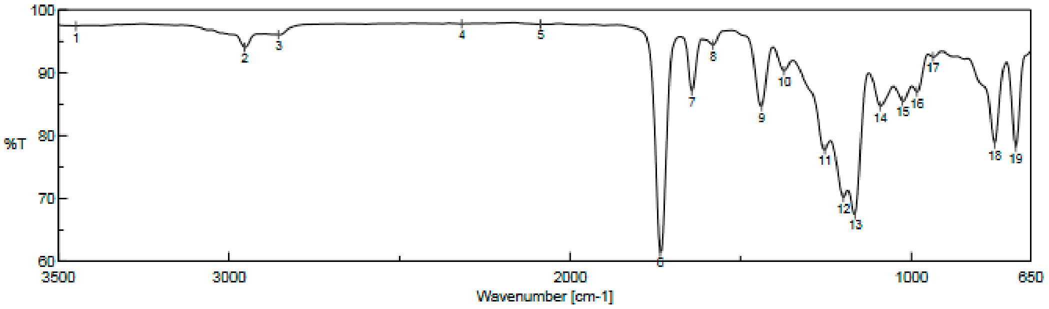

[Comments]
Sample name
Comment
User
Division
Company Universidad de Alicante

[Result of Peak Picking]

No.	Position	Intensity	No.	Position	Intensity	No.	Position	Intensity	No.	Position	Intensity
1	3448.1	97.42	2	2954.41	94.01	3	2854.13	96.03	4	2318.02	97.75
5	2086.6	97.69	6	1735.62	61.49	7	1643.05	87.07	8	1581.34	94.40
9	1438.64	84.66	10	1373.07	90.29	11	1253.5	77.73	12	1199.51	70.17
13	1164.79	67.48	14	1091.51	84.69	15	1025.94	85.47	16	983.518	86.92
17	937.235	92.48	18	755.959	78.72	19	694.248	78.19			

Figure S56, FTIR of compound **2a**

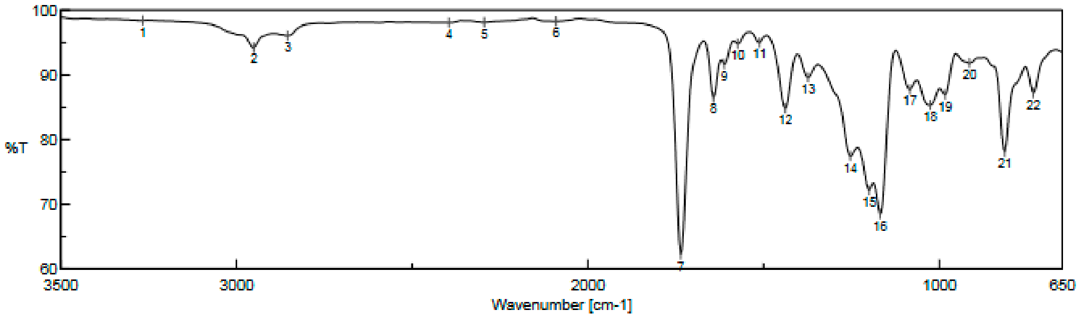

[Comments]
Sample name
Comment
User
Division
Company Universidad de Alicante

[Result of Peak Picking]

No.	Position	Intensity	No.	Position	Intensity	No.	Position	Intensity	No.	Position	Intensity
1	3266.82	98.37	2	2950.55	94.22	3	2854.13	96.04	4	2395.16	98.06
5	2294.88	98.10	6	2090.46	98.24	7	1735.62	62.22	8	1643.05	86.50
9	1612.2	91.66	10	1573.63	94.80	11	1511.92	94.98	12	1438.64	84.80
13	1373.07	89.60	14	1253.5	77.45	15	1199.51	72.14	16	1168.65	68.48
17	1083.8	87.71	18	1025.94	85.30	19	983.518	86.92	20	914.093	91.80
21	813.813	78.08	22	732.817	87.17						

Figure S57, FTIR of compound **2b**

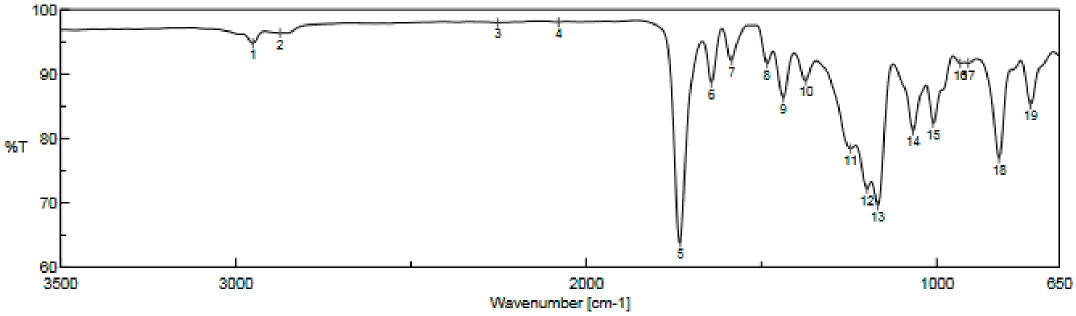

[Comments]
Sample name
Comment
User
Division
Company Universidad de Alicante

[Result of Peak Picking]

No.	Position	Intensity	No.	Position	Intensity	No.	Position	Intensity	No.	Position	Intensity
1	2950.55	94.75	2	2873.42	96.37	3	2252.45	97.95	4	2078.89	98.06
5	1731.76	63.81	6	1643.05	88.61	7	1585.2	92.10	8	1484.92	91.61
9	1438.64	86.27	10	1373.07	88.94	11	1245.79	78.41	12	1199.51	72.15
13	1168.65	69.56	14	1068.37	81.21	15	1010.52	82.33	16	933.378	91.66
17	910.236	91.67	18	821.527	76.84	19	732.817	85.28			

Figure S58, FTIR of compound **2g**

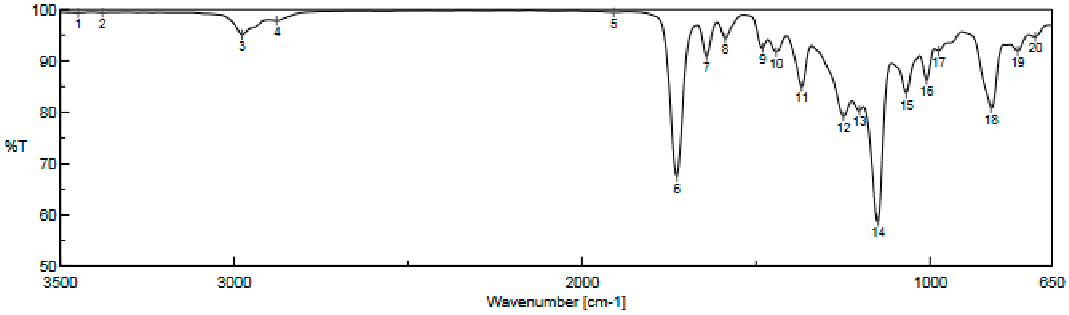

[Comments]
Sample name
Comment
User
Division
Company Universidad de Alicante

[Result of Peak Picking]

No.	Position	Intensity	No.	Position	Intensity	No.	Position	Intensity	No.	Position	Intensity
1	3448.1	99.34	2	3378.67	99.37	3	2977.55	95.08	4	2877.27	97.84
5	1909.18	99.45	6	1727.91	67.45	7	1643.05	90.86	8	1589.06	94.40
9	1481.06	92.54	10	1442.49	91.71	11	1369.21	84.95	12	1249.65	79.17
13	1203.36	80.34	14	1149.37	58.75	15	1066.37	83.63	16	1010.52	86.26
17	975.804	91.91	18	825.384	80.77	19	748.245	91.88	20	698.105	94.65

Figure S59, FTIR of compound **2h**

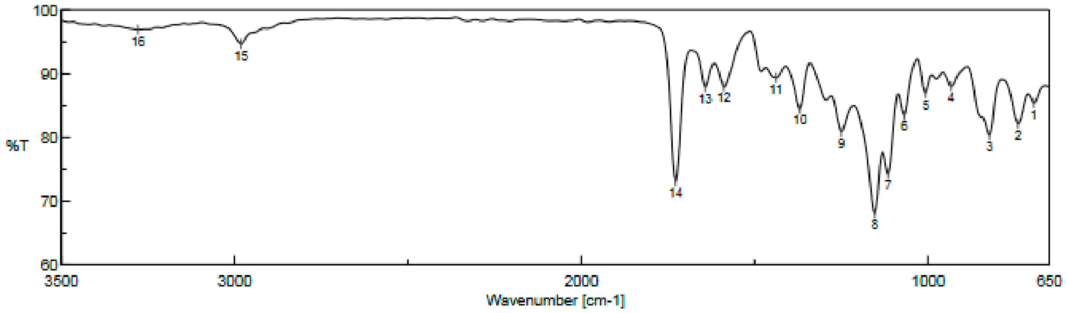

[Comments]
Sample name
Comment
User
Division
Company Universidad de Alicante

[Result of Peak Picking]

No.	Position	Intensity	No.	Position	Intensity	No.	Position	Intensity	No.	Position	Intensity
1	694.248	85.38	2	740.531	82.18	3	821.527	80.33	4	933.378	88.01
5	1006.66	86.94	6	1068.37	83.48	7	1114.65	74.25	8	1153.22	67.99
9	1249.65	80.89	10	1369.21	84.47	11	1438.64	89.39	12	1589.06	87.96
13	1643.05	87.90	14	1727.91	73.06	15	2981.41	94.69	16	3278.39	96.92

Figure S60, FTIR of compound **2m**

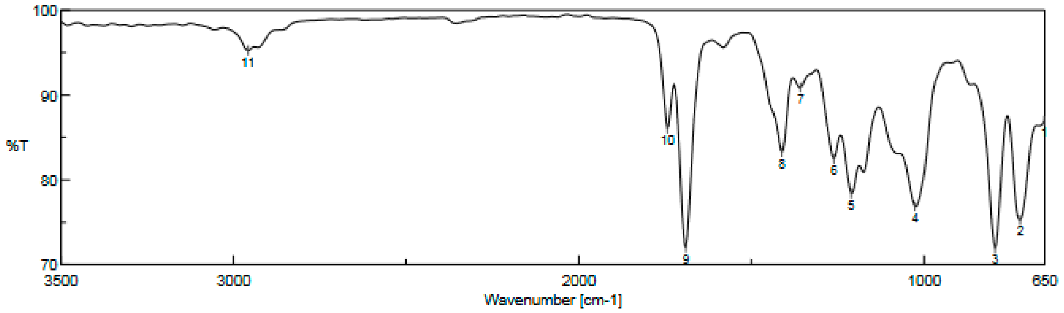

[Comments]
Sample name
Comment
User
Division
Company Universidad de Alicante

[Result of Peak Picking]

No.	Position	Intensity	No.	Position	Intensity	No.	Position	Intensity	No.	Position	Intensity
1	651.822	86.92	2	721.247	75.26	3	794.528	71.92	4	1025.94	76.85
5	1211.08	78.40	6	1261.22	82.48	7	1357.64	90.87	8	1411.64	83.23
9	1689.34	72.03	10	1743.33	86.04	11	2958.27	95.24			

Figure S61, FTIR of compound **8a**

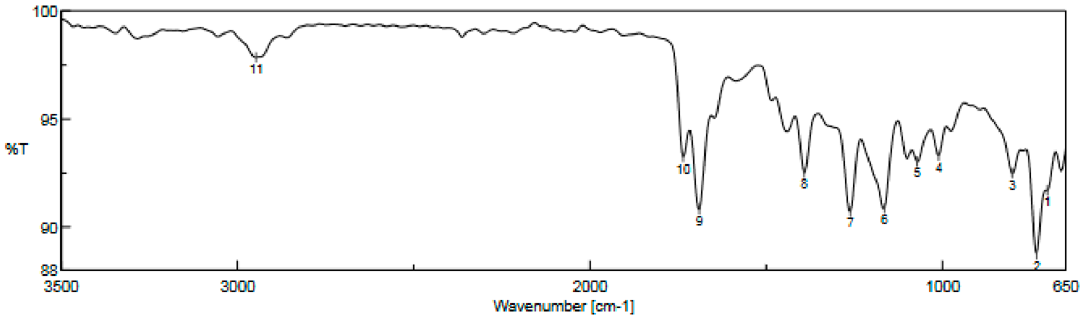

[Comments]
Sample name
Comment
User
Division
Company Universidad de Alicante

[Result of Peak Picking]

No.	Position	Intensity	No.	Position	Intensity	No.	Position	Intensity	No.	Position	Intensity
1	701.962	91.68	2	732.817	88.73	3	802.242	92.49	4	1010.52	93.30
5	1072.23	93.02	6	1164.79	90.86	7	1261.22	90.69	8	1392.35	92.50
9	1689.34	90.81	10	1735.62	93.24	11	2946.7	97.86			

Figure S62, FTIR of compound **8b**

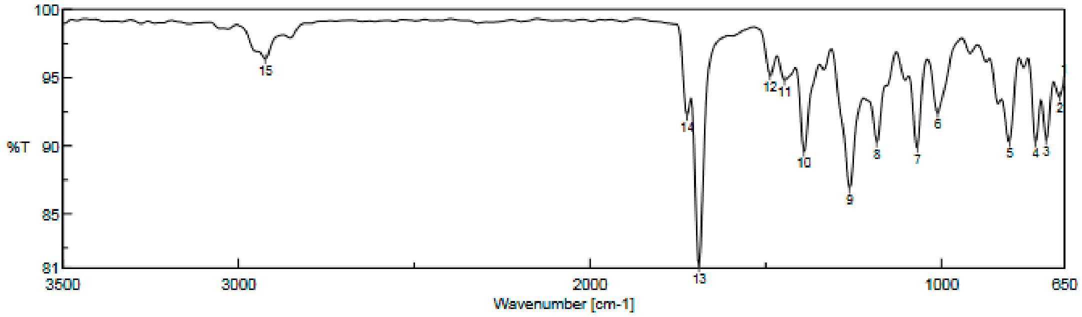

[Comments]
Sample name
Comment
User
Division
Company Universidad de Alicante

[Result of Peak Picking]

No.	Position	Intensity	No.	Position	Intensity	No.	Position	Intensity	No.	Position	Intensity
1	651.822	94.70	2	663.393	93.59	3	701.962	90.38	4	732.817	90.25
5	806.099	90.26	6	1010.52	92.39	7	1068.37	89.81	8	1184.08	90.22
9	1261.22	86.81	10	1392.35	89.54	11	1446.35	94.78	12	1488.78	95.16
13	1689.34	81.06	14	1724.05	92.19	15	2923.56	96.35			

Figure S63, FTIR of compound **8c**

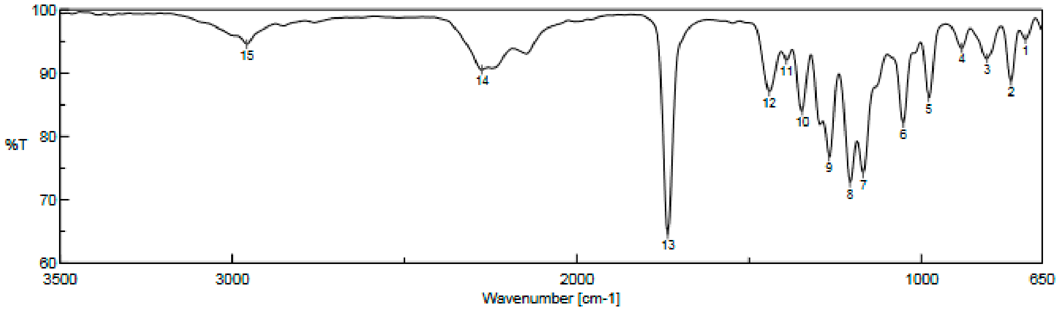

[Comments]
Sample name
Comment
User
Division
Company Universidad de Alicante

[Result of Peak Picking]

No.	Position	Intensity	No.	Position	Intensity	No.	Position	Intensity	No.	Position	Intensity
1	696.105	95.34	2	740.531	88.76	3	809.956	92.30	4	883.238	93.94
5	979.661	86.19	6	1052.94	82.14	7	1168.65	74.17	8	1207.22	72.73
9	1268.93	76.80	10	1346.07	84.00	11	1392.35	92.06	12	1442.49	87.11
13	1735.62	64.55	14	2275.59	90.50	15	2958.27	94.55			

Figure S64, FTIR of compound **9**
